# Alteration of tumor suppressor BMP5 in sporadic colorectal cancer: a genomic and transcriptomic profiling based study

**DOI:** 10.1186/s12943-018-0925-7

**Published:** 2018-12-20

**Authors:** Erfei Chen, Fangfang Yang, Hongjuan He, Qiqi Li, Wei Zhang, Jinliang Xing, Ziqing Zhu, Jingjing Jiang, Hua Wang, Xiaojuan Zhao, Ruitao Liu, Lei Lei, Jing Dong, Yuchen Pei, Ying Yang, Junqiang Pan, Pan Zhang, Shuzhen Liu, Le Du, Yuan Zeng, Jin Yang

**Affiliations:** 1 Institute of Preventive Genomic Medicine, School of Life Sciences, Northwest University, Xian, 710069 China; 2 Key Laboratory of Resource Biology and Biotechnology in Western China, Ministry of Education, School of Life Sciences, Northwest University, Xian, 710069 China; 3 Department of Pathology, The Helmholtz Sino-German Laboratory for Cancer Research, Tangdu Hospital, the Fourth Military Medical University, Xian, 710038 China; 4 State Key Laboratory of Cancer Biology and Experimental Teaching Center of Basic Medicine, Fourth Military Medical University, Xian, China; 5 Laboratory of Systems Biology, Shanghai Advanced Research Institute, Chinese Academy of Sciences, No. 100 Haike Road, Zhangjiang Hi-Tech Park, Pudong, Shanghai, 201210 China

**Keywords:** BMP5, Tumor suppressor, Loss of function mutation, Driver gene, Sporadic colorectal cancer

## Abstract

**Background:**

Although the genetic spectrum of human colorectal cancer (CRC) is mainly characterized by *APC*, *KRAS* and *TP53* mutations, driver genes in tumor initiation have not been conclusively demonstrated. In this study, we aimed to identify novel markers for CRC.

**Methods:**

We performed exome analysis of sporadic colorectal cancer (sCRC) coding regions to screen loss of function (LoF) mutation genes, and carried out systems-level approaches to confirm top rank gene in this study.

**Results:**

We identified loss of BMP5 is an early event in CRC**.** Deep sequencing identified BMP5 was mutated in 7.7% (8/104) of sCRC samples, with 37.5% truncating mutation frequency. Notably, BMP5 negative expression and its prognostic value is uniquely significant in sCRC but not in other tumor types. Furthermore, BMP5 expression was positively correlated with E-cadherin in CRC patients and its dysregulation play a vital role in epithelial-mesenchymal transition (EMT), thus triggering tumor initiation and development. RNA sequencing identified, independent of BMP/Smads pathway, BMP5 signaled though Jak-Stat pathways to inhibit the activation of oncogene *EPSTI1*.

**Conclusions:**

Our result support a novel concept that the importance of BMP5 in sCRC. The tumor suppressor role of BMP5 highlights its crucial role in CRC initiation and development.

**Electronic supplementary material:**

The online version of this article (10.1186/s12943-018-0925-7) contains supplementary material, which is available to authorized users.

## Background

Sporadic colorectal cancer (sCRC) accounts for about 80% of colorectal cancer and is one of the most common cancer types worldwide. The prevalence of sCRC increased over the past years. One of the fundamental processes driving the initiation and progression of sCRC is the accumulation of a variety of genetic and epigenetic changes in colonic epithelial cells. Presently, genetic prediction for sCRC is typically dependent upon a limited number of assays that analyze a small number of biomarkers of distinct types, including *APC, KRAS,* and *TP53* gene mutations, as well as a subset of DNA methylation and non-coding RNA regulation [1–3]. Recent study uncovered the number of coding region mutations in the entire tumor (3.5 cm in diameter) was estimated to be greater than 100 million under the non-Darwinian mode [4]. The genetic diversity of tumor is more than expected, and the prevailing database of mutation spectrum is challenged. With the advancement of new omics technologies including whole genome, exome and transcriptome sequencing, it is now possible for us to screen for new causative genes for sCRC at omics scale, and to further study the main mechanisms of gene alterations and related signaling pathways [2, 3, 5]. Here, we reported that by exome sequencing and public data analysis, bone morphogenetic protein 5 (*BMP5*) was identified as a novel tumor suppressor gene in sCRC, and its downstream genes was characterized by RNA sequencing.

BMP5 has been previously studied in myeloma, adrenocortical carcinoma, and breast cancer. However, in digestive tract tumors, especially CRC, the role of BMP5 is unclear. In this investigation, we aimed to determine the difference of BMP5 expression and genetic alteration in seven tumor types. Based on public databases and our clinical samples, the consistency of loss of BMP5 at DNA, RNA and protein level confirmed its importance and tumor suppressor role in CRC. We further validated the tumor inhibition impact of BMP5 in CRC both in vitro and in vivo. RNA sequencing revealed that BMP5 was involved in Jak-Stat signaling pathway. Our results suggest that loss of BMP5 plays a vital role in CRC initiation and progression.

## Material and methods

### Human sporadic colorectal cancer tissues and cell lines

All patients included in this study had written informed consent. DNA samples used for whole exome sequencing were extracted from 3 cases of fresh frozen tumor tissues. For cases used in expanded deep sequencing, tumor DNA and matched normal tissue DNA were extracted using QIAamp DNA FFPE Tissue Kit (QIAGEN). 28 cases of validation samples were obtained from preclinical medical teaching experiment center of Fourth Military Medical University. All samples were confirmed with no family history of colorectal cancer and the pathological information were listed in Additional file 1: Table S1.

Human colorectal cancer cells (HT-29, LoVo, HCT 116, and SW480) were purchased from ATCC (American Type Culture Collection), and immortal normal epithelial cells NCM460 were purchased from INCELL (San Antonio).

### Exome sequencing and variant analysis

Exome capture was performed using the TruSeq Exome Enrichment Kit (Illumina) according to the manufacturer’s instructions. Sequencing was performed on the Illumina Hiseq2000 platform. The variant calling was analyzed using GATK tools as previously described [6]. We performed loss of function mutation screening strategy (Additional file 2: Figure S1). The remaining candidates are subsequently filtered out with the following criteria: 1. Reported SNVs in dbSNP130 and 1000 genomes were removed. 2. The SNVs quality score in tumor must be ≥100. 3. The sequencing depth of every SNV must be between 10 and 200. 4. The SNVs in the last 5% of gene coding region were removed. High confidence novel SNVs were resequenced in original samples and matched blood samples to confirm somatic SNVs. Primers used for expanded sequencing of BMP5 coding regions were shown in Additional file 1: Table S2.

### RNA extraction and real-time quantitative PCR analysis

Total RNA Miniprep Kit (Sigma, St Louis, MO) was used to extract RNA from cell lines according to the manuals. The isolated RNA was then reverse transcribed to cDNA (Takara, Japan). Quantitative real-time PCR amplifications were performed using the SYBR Premix Ex Taq™ II kit (TAKARA, Japan) by CFX96™ real-time PCR detection system. Primers used in this study can be found in Additional file 1: Table S3.

### BMP5 immunohistochemistry

BMP5 immunohistochemistry was performed on tumor microarrays (TMAs) from Fanpu Biotech, Inc., and the pathological information can be found in Additional file 1: Table S4. For antigen retrieval, tissue sections were boiled in 0.01 M citrate solution (pH 6.0) and incubated with primary antibody to BMP5 (1:200, Proteintech, China). Tissue sections were observed with a standard light microscope (Leica). The intensity of staining ranged from negative (0) to weak positive (1–2) or strong positive (3).

### Western blotting

Protein extraction and blotting was performed as described previously [7]. Western blots were probed with the following antibodies anti-BMP5 (Abcam, ab88064, 1:500), anti-PCNA (Immunoway, 1:5000), anti-MMP2 (Proteintech, 1:500), anti-MMP9 (Proteintech, 1:1000), anti-E-cadherin (BD Biosciences, 1:1000), anti-EPSTI1 (Proteintech, 1:500) and anti-GAPDH (Immunoway, 1:5000).

### Immunofluorescence staining

5 × 10^3^ SW480 or NCM460 cells were seeded into 24-well plates for cell climbing. Cells were fixed with 4% paraformaldehyde for 15 min, blocked with 1% bovine serum albumin at ambient temperature for 1 h and then incubated with rabbit anti-BMP5 primary antibody (1:100) at 4 °C overnight. Cells were incubated with goat anti-rabbit IgG secondary antibody (Abbine, USA, 1:800) at 37 °C, and with nuclear stained by DAPI (SouthernBiotech, USA) subsequently. Images were filmed and analyzed by Fluview FV10i.

### Luciferase reporter assay

The entire 3’-UTR of human BMP5 was amplified and then inserted into the pmiRGLO vector (Promega, Madison, WI, USA). To evaluate binding specificity, the sequences that interact with the seed region of miR-25, miR-32, miR-92a, and miR-655 were mutated respectively using KOD-Plus Mutagenesis Kit (Toyobo, Japan). For luciferase reporter assays, we performed as described previously [8].

### Construction of wild type and mutant BMP5 expression vector

The full-length cDNA of BMP5 was obtained from 293 T cDNA by PCR using the primers described in Additional file 1: Table S5. PCR products were then cloned into the BamHI/SalI sites of pEF-BOS-EX. The p. D183G mutation was introduced using KOD -Plus- Mutagenesis Kit (Toyobo, Japan). Transfection was carried out using X-tremeGENE HP DNA Transfection Reagent (Roche Applied Sciences).

### Lentiviral infection

For BMP5 overexpression, full length CDS of BMP5 was inserted in pLV5 vector. Lentiviral stocks were prepared in HEK-293 T cells. Viral supernatant mixed with 5 μg/ml polybrene was used to infect HT-29 or SW480 cells for 24 h. Cells were then selected by puromycin for stable BMP5 overexpression.

### siRNA transfection

The sequences of the two siRNAs used are listed in Additional file 1: Table S6. SW480 cells were transfected using HiPerFect transfection reagent (Qiagen, Valencia, CA, USA) at a confluency of 50%. The final concentration of siRNAs in SW480 cells was 20 nM.

### Proliferation assay

For proliferation assay, Cell Counting Kit-8 (Dojindo Laboratories, Japan) was used according to the manufacturer’s instructions. The number of viable cells was measured at a wavelength of 450 nm.

### Flow cytometry analyses

Cells were collected and washed twice with PBS after 48 h post transfection. For cell cycle analysis, the cells were fixed with 70% ethanol overnight at 4 °C, and then washed with PBS, resuspended with 500 μl buffer and then incubated with 100 μg/ml RNaseA and 50 μg/ml propidium iodide (PI) (7sea biotech) for 30 min at 37 °C. After incubation, the cells were subjected to DNA content analysis using a FACSCalibur (Beckman Coulter, Fullerton, CA) and the results were analyzed with the Summit v4.3 software. For apoptosis assay, the method was described previously [7].

### Migration and invasion assay

Cell migration or invasion ability was evaluated using Transwell chambers (Corning, MA, USA) without or with Matrigel (BD Biosciences, Bedford, MA, USA). Cells were treated with Mitomycin C (10 μg/ml) to inhibit cell proliferation before plating. 10^5^ SW480 cells were suspended in 100 μl serum-free medium and then plated in the upper chamber of the 24-Well, with 700 μl medium containing 10% FBS in the lower chamber. The cells were incubated at 37 °C for 48 h. Detail method was described previously [7].

### Tumor formation in nude mice

We adhered to standards articulated in the Animal Research: Reporting of In Vivo Experiments (ARRIVE) guidelines [9]. Male balb/c nude mice (3–5 weeks old) were purchased from Animal Center at Medical College, Xi’an Jiaotong University. All mice were housed and maintained under specific pathogen-free conditions. 2 × 10^6^ cells (BMP5 wild or mutant type stable expressing HT-29 cells) were resuspended in 200 μl serum-free medium and then injected to the subcutaneous of the right axilla of nude mice (*n* = 6 mice/group). The tumors’ dimensions were monitored with vernier calipers for a total period of 25 days, and the tumor volume was calculated using the following formula: 0.5 × a × b^2^, where a represents the longer diameter and b represents the corresponding perpendicular shorter diameter.

### Transcriptome sequencing and analysis

After transfection of BMP5 and control vector, total RNA was extracted using TRIzol reagent (Invitrogen, Carlsbad, CA, USA). RNA-seq and bioinformatic data analysis were performed by Shanghai Novelbio Ltd. We applied DEseq algorithm to filter the differentially expressed genes, after the significant analysis and following criteria that fold change > 1.5 or < 0.667. A Fisher exact test was used to find the significant enrichment pathway. The resulting *P* values were adjusted using the Benjamini and Hochberg (BH) FDR algorithm. Gene coexpression Networks were built according to the normalized signal intensity of specific expression genes. For each pair of genes, we calculate the pearson correlation and choose the significant correlation pairs to constructed the network.

### Statistical analysis

The data were shown as Mean ± S.E.M or Mean ± SD. Paired or unpaired *t*-test, Wilcoxon test, Chi-square test, Pearson’s correlation and Kaplan-Meier survival analysis were used to compare the differences among groups. *P* value < 0.05 was considered as statistically significant. *P < 0.05, **P < 0.01, ***P < 0.001. ****P < 0.0001.

## Results

### Whole exome sequencing identifies somatic loss of function mutations in sCRC

**Fig. 1:**
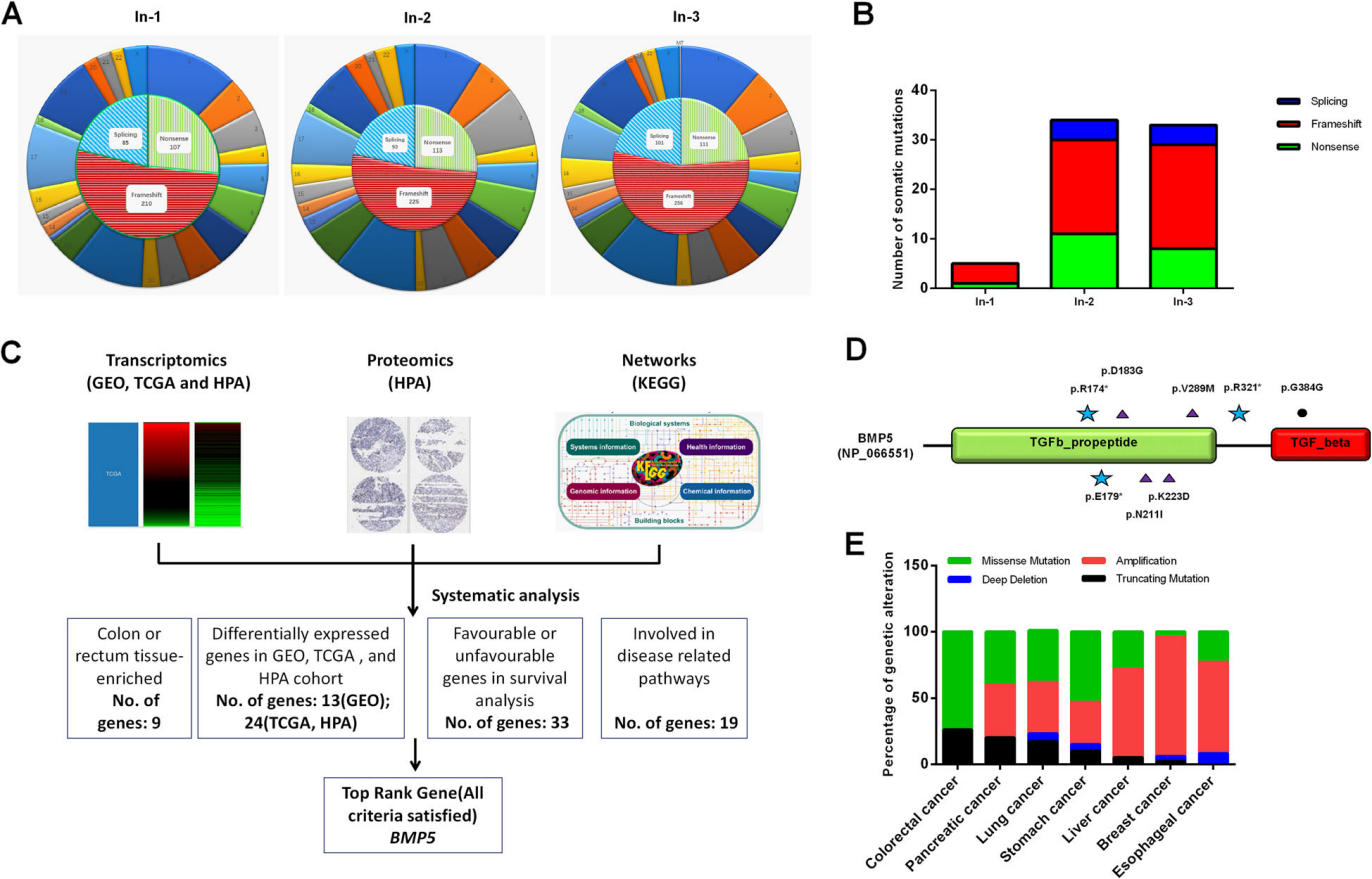
Exome sequencing and transcriptomic analysis identified BMP5 as novel tumor suppressor gene in sCRC. **a** Sketch map of LoF mutations in 3 sCRC samples. Outer circle represents the distribution of mutation on each chromosome, and inner circle represents the quantity and proportion of three types of mutations. **b** Distribution of 72 somatic mutations in 3 samples. **c** Systematic analysis of 71 genes at system-level and selection of top rank gene *BMP5* in this study. **d** Deep sequencing of BMP5 coding regions identified novel somatic mutations in sCRC samples. Relative positions of mutations are indicated by symbols. Stars, nonsense; Triangles, missense; Dots, synonymous. **e** Genetic alteration analysis of BMP5 in seven solid tumors

Exome capture and sequencing was performed on three cases of sCRC tumor DNAs. We focused on the loss of function (LoF) mutations that may greatly disrupt protein translation (Additional file 2: Figure S1). A total of 1301 LoF mutations were observed which was in line with the results previously reported in Asian samples (Fig. 1a) [10]. To determine which of these alterations were somatic (sCRC-specific), we resequenced the high confidence novel SNVs using Sanger sequencing, filtered the mutations that observed in the matched normal blood samples. In total 72 somatic mutations were identified, of which 20 were nonsense, 18 at canonical splice site, and 40 frameshift (Fig. 1b and Additional file 1: Table S7).

### Systems-level analysis confirms *BMP5* as top rank gene in sCRC

To further narrow the genes screened, we used data from GEO, TCGA, HPA, and KEGG to perform a systems-level analysis of 71 mutated genes (Fig. 1c), aimed to identify novel markers that can affect CRC initiation, progression and patients’ survival. Our hypothesis is normal tissue-enriched genes may be tissue-specific, and loss of which could promote CRC tumorigenesis. In order to find colorectal-enriched genes that may be colorectal tissue-specific, we analyzed gene expression level in normal tissues using HPA and TCGA database. The gene expression in normal colon or rectum ranked top 7 in 37 tissues was identified as colorectal-enriched in this study. 11 genes are not detected in colon or rectum, while *BMP5*, *REP15*, *ATP8B1*, *ELF3*, and *RASSF6* are relative high in colorectal tissue, and significantly downregulated in colon or rectum adenocarcinomas (Additional file 1: Table S8). We further analyzed differentially expressed genes in early events of CRC (normal tissue – adenoma or polyps) using GEO datasets (GSE8671, GSE71187, and GSE41258). We found 13 genes were differentially expressed in at least 2 series, of which 7 were downregulated (*BMP5, PDE2A, ZNF175, CTSA, SVEP1, ATP6V0D2,* and *AHNAK*) (Additional file 1: Table S9). Kaplan Meier survival analysis identified 33 genes may affect patients’ survival, including two favourable prognostic markers (*REP15*, *ATP8B1*) and one unfavourable prognostic marker (*GPSM1*) reported in HPA database [11]. In addition, high expression of *BMP5* and *RASSF6* were correlated with a longer patients’ survival outcome (Additional file 1: Table S10). To find disease-related genes, we further used KEGG database together with literature queries, 19 genes were involved in pathway networks, including *APC, TCF7L2* (Wnt signaling), *BMP5* (TGFβ signaling), and *RASSF6* (Hippo signaling) (Additional file 1: Table S11). Taken together, *BMP5* satisfied all criteria we tested in multi-omics. We confirmed *BMP5,* a novel gene that has never been investigated in sCRC, was top rank gene in our study. From the cBioPotal database, we found 30.4% of *BMP5* mutated samples without *APC* mutation, and still 17.4% of *BMP5* mutated samples without *APC, KRAS, or TP53* mutation. As is well known, most sCRCs occur through chromosomal instability pathway, which is characterized by *APC* mutations. This result may indicate that in addition to well-studied diver genes, the alteration of *BMP5* may play a role in the oncogenesis of sCRC.

Subsequent Sanger sequencing analysis in expanded individuals showed *BMP5* was mutated in 7.7% of patients and 37.5% of these mutations were LoF. The distribution of 8 mutations identified in BMP5 is shown in Fig. 1d, Table 1, and Additional file 2: Figure S2. Notably, we also found a missense mutation p. D183G in In-2 patient. All missense mutations found were possibly damaging and pathogenic analyzed by PolyPhen-2 and FATHMM-MKL algorithm [12, 13]. The affected residues of BMP5 are highly conserved evolutionarily (Additional file 2: Figure S3), thus these mutations are rare in normal but of high penetrance in sCRC. Examination of publicly available databases revealed that *BMP5* mutation is also found in several other tumor types (Fig. 1e). Truncating mutation frequency of BMP5 is highest in CRC, while copy number amplification could be found in all tumors but not in CRC. These results showed the characteristic of BMP5 alteration is different from that of other type of tumors.

**Table 1:** *BMP5* somatic mutations identified in exome sequencing and expanded deep sequencing cases

Sample	Mutation type	Exon	Position	Base change	Amino acid change
In-1	NONSENSE	2	Chr6:55684616	C > T	p. R174*
In-2	MISSENSE	2	Chr6:55684588	A > G	p. D183G
Ex-50	NONSENSE	2	Chr6:55684601	G > T	p. E179*
Ex-36	MISSENSE	2	Chr6:55684504	A > T	p. N211I
Ex-92	MISSENSE	2	Chr6: 55684467	G > T	p. K223D
Ex-93	NONSENSE	4	Chr6:55638913	C > T	p. R321*
Ex-70	MISSENSE	4	Chr6:55639009	G > A	p. V289 M
Ex-2	SYNONYMOUS	6	Chr6:55623866	A > G	p. G384G

### BMP5 is downregulated in sCRC and its low expression correlates with recurrence and poor prognosis

**Fig. 2:**
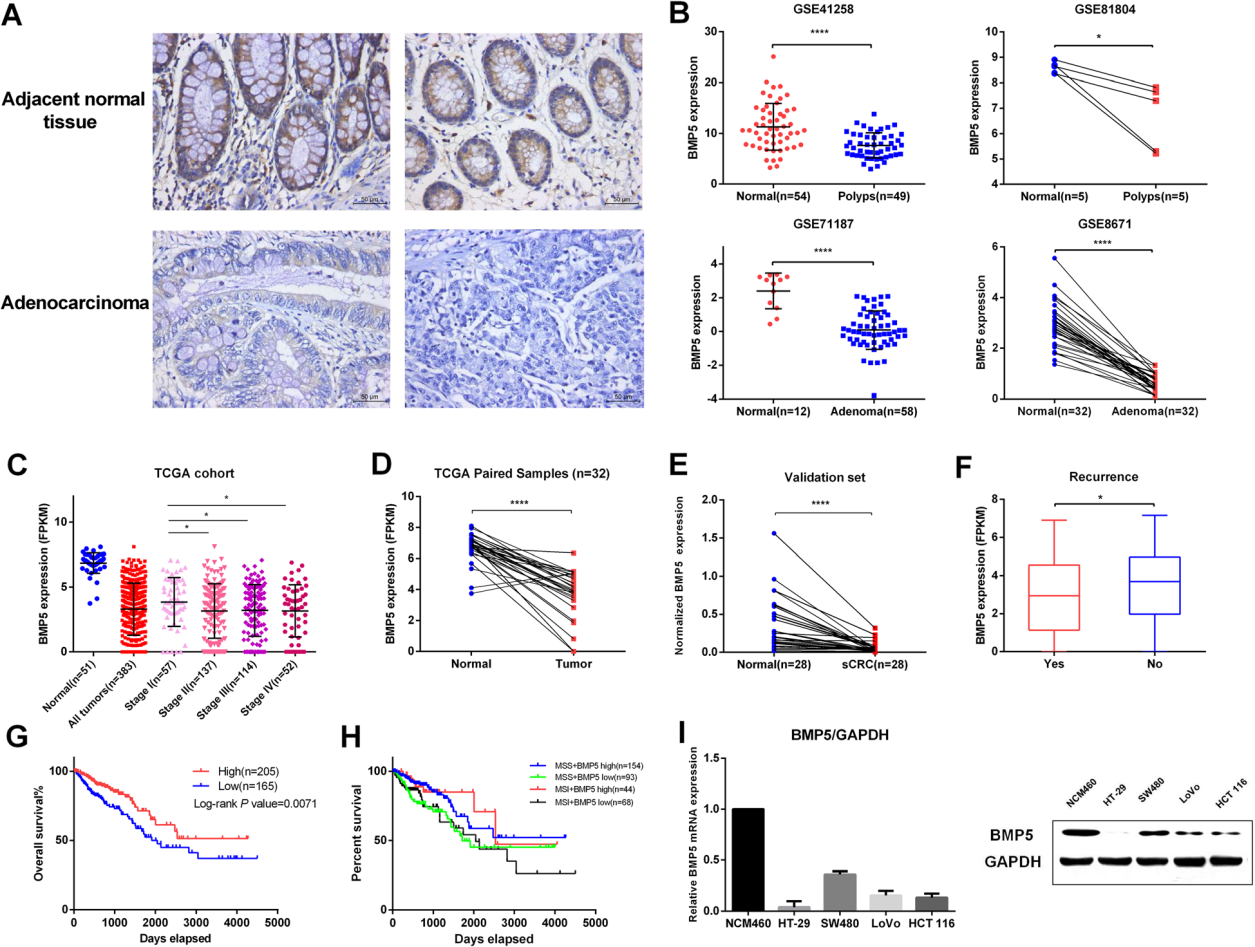
BMP5 is downregulated in sCRC and its low expression correlates with recurrence and poor prognosis. **a** Representative images of Immunohistochemical staining of BMP5 expression in sCRC (lower) and adjacent normal tissue (upper). BMP5-positive cells mainly located in adjacent benign glands. Four tissue microarrays containing 129 paired samples of CRC and normal colon tissue was assessed by immunohistochemistry. Scale bars represent 50 μm. Sample information: (Left) female, 57, high differentiation; (Right), male, 73, low differentiation. **b** BMP5 expression analysis in normal colorectal tissue and adenomas (polyps) of GEO datasets (GSE41258, GES81804, GSE71187, and GSE8671), *P < 0.05, ****P < 0.0001. **c** BMP5 mRNA levels were assessed using TCGA CRC data,*P < 0.05. **d** Paired samples were selected and analyzed using paired t test, **** *P* < 0.0001. **e** Validation group of 28 matched sCRC samples identified BMP5 was significantly decreased in tumor samples (****P < 0.0001, paired t-test). **f** Correlation analysis of BMP5 expression and patients’ recurrence, *P < 0.05. **g** Kaplan–Meier analysis of overall survival according to high or low BMP5 expression in adenocarcinomas (Log rank test). **h** Kaplan–Meier analysis of overall survival according to BMP5 expression and MSI status. (**i**) mRNA and protein level of BMP5 in colon cell lines. All four cancer cell lines showed downregulated of BMP5 as compared to normal NCM460 cells

To test whether loss of BMP5 is common in cancer, we firstly verified the protein level of BMP5 in seven tumor types using immunohistochemistry (IHC). Our results showed that BMP5 expression was negative in 31.0% (40 of 129) of the sCRC tumor cases while 10.1% (13 of 129) of the normal tissues (Chi-Square tests *P* < 0.0001, Fig. 2a and Table 2), and BMP5 expression showed no correlation to age, sex and clinical grade of 129 colorectal adenocarcinomas (Additional file 1: Table S12). However, BMP5 expression showed no significant difference between tumor and normal tissues in hepatic, esophagus, gastric, pancreatic, and lung, while in breast cancer, BMP5 positive was significantly higher in Invasive ductal carcinoma than normal control (52.5% vs 12.5%, *P* = 0.0003, Table 2). This may demonstrate loss of BMP5 may be a unique event in sCRC.

**Table 2:** Expression of BMP5 in different type of tumors (tissue microarrays)

Organ	Tissue type	Total no. of samples	No. of BMP5 positive samples	%positive (No./total)	*P* value
Colorectal	Normal	129	116	89.9	**< 0.0001**
	Adenocarcinoma	129	89	69.0	
Liver	Normal	50	50	100	1
	Hepatocellular carcinoma	50	50	100	
Esophagus	Normal	50	40	80	1
	Squamous cell carcinoma	50	41	82	
Stomach	Normal	50	46	92	1
	Adenocarcinoma	50	46	92	
Pancreas	Normal	50	25	50	0.6889
	Adenocarcinoma	50	28	56	
Lung	Normal	50	0	0	1
	Adenocarcinoma	50	0	0	
Breast	Normal	40	5	12.5	**0.0003**
	Invasive ductal carcinoma	40	21	52.5	

We further explored the correlation of BMP5 expression with clinicopathological features using data from GEO and The Cancer Genome Atlas (TCGA). We analyzed GEO datasets that recruit normal tissue and adenomas (polyps) and found BMP5 downregulation is an early event in CRC (Fig. 2b). TCGA data showed BMP5 was significantly downregulated in CRC (Wilcoxon test, *P* = 7.797e^− 28^) (Fig. 2c), and early stage (Stage I) patients showed higher expression than other stage patients (unpaired t test, *P* < 0.05). Furthermore, paired samples analysis revealed that 93.8% (30 of 32) cases showed downregulatiton of BMP5 (Fig. 2d, paired t test, *P* < 0.0001), which was also confirmed in our validation group (Fig. 2e, paired t test, *P* < 0.0001). Low expression of BMP5 was correlated with patients’ recurrence (Fig. 2f and Additional file 1: Table S13) as well as poor survival (Fig. 2g, Log rank *P* = 0.0071). Notably, survival analysis in other 6 tumors we tested did not show this significance (Additional file 2: Figure. S4). Microsatellite instability (MSI) is a favourable prognostic marker for CRC, and we found five-year survival is higher in MSI patients with high BMP5 expression than other groups (Fig. 2h). Moreover, low BMP5 expression is correlated with poor survival independent of MSI status.

For the endogenous expression analysis in cell lines, BMP5 showed the highest level in normal NCM460 cells. Among CRC cell lines, BMP5 was relatively high in SW480 cells and barely expressed in HT-29 cells (Fig. 2i). In NCM460 and SW480 cells, BMP5 is mainly localized to vesicles, which is similar to its homolog BMP7 (Additional file 2: Figure S5).

### Upstream oncogenic miRNAs partially contribute to the decreased level of BMP5

**Fig. 3:**
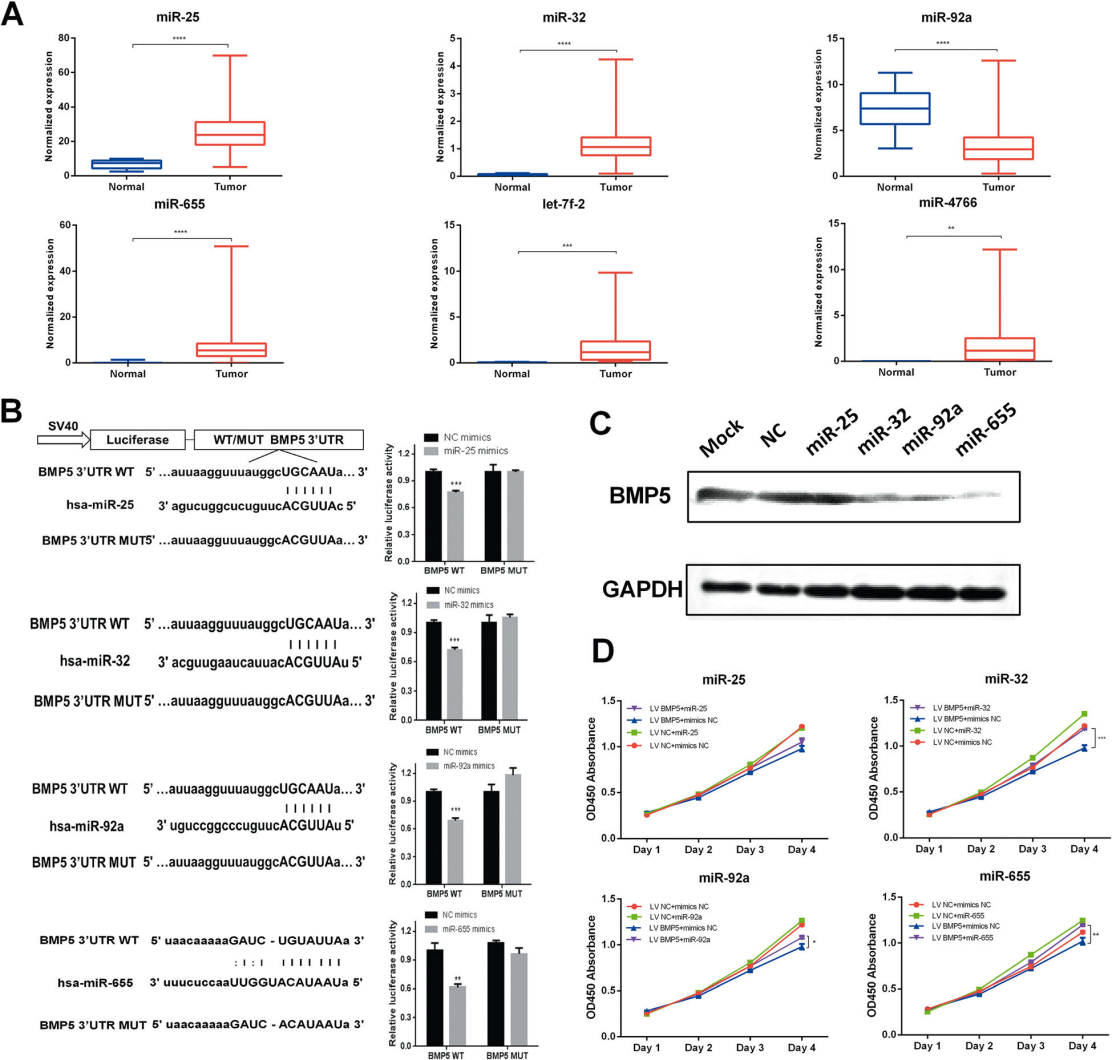
Oncogenic miRNAs contributed decreased level of BMP5. **a** Expression analysis of miRNAs in CRC samples from TCGA database. The bar represents min-max value. **b** Schematic description of wild type (WT) and mutated 3’ UTRs of BMP5. The WT and MUT 3’ UTR sequences were cloned into the pmiRGLO vector. Luciferase analysis was used to detect the reporter activity in SW480 cells after 24 h of transfection according to manuals. **(c)** miR-32, miR-92a, and miR-655 inhibit the protein levels of BMP5 in SW480 cells. SW480 cells were transfected with the miRNA mimics, negative control (NC), or transfection reagent control (Mock), respectively. **d** Overexpression of BMP5 could reverse the cell proliferation ability after transfection of miR-32, miR-92, or miR-655. The data are showed as mean ± sem. *P < 0.05, **P < 0.01, ***P < 0.001

To clarify the factor for decreased level of BMP5 in addition to loss of function mutations, we utilized bioinformatic analyses to find potential epigenetic changes in BMP5. CpG island was not found in BMP5 promoter using MethPrimer tools [14], and studies about hypermethylation of BMP5 was not reported yet. Meanwhile, prediction of BMP5 3’-UTR binding miRNAs using TargetScan, miRanda, and together with TCGA expression analysis helped lead to the selection of previously reported oncogenic miRNAs (miR-32, miR-92a) or upregulated miRNAs (miR-25, miR-655, let-7f-2, and miR-4766) in CRC (Fig. 3a). In CRC samples of TCGA, BMP5 expression was only negatively correlated with miR-32 and miR-655 expression, mainly in stage IV patiens (Additional file 2: Figure S6). We finally selected four miRNA for binding analysis, among which miR-32, miR-25, and miR-92 share the same seeding match sequence (Position 51–57 of BMP5 3’ UTR). Dual luciferase assay demonstrated these four miRNAs could significantly repress the reporter activity (wild-type 3’-UTR of BMP5) (Fig. 3b). Further Western blotting showed these miRNAs could inhibit the expression of BMP5 in varying degrees (Fig. 3c). We also found miR-32 and miR-655 could partially reverse the tumor inhibition ability by stable BMP5 expression in SW480 cells (Fig. 3d).

### BMP5 inhibits CRC cell proliferation both in vitro and in vivo

**Fig. 4:**
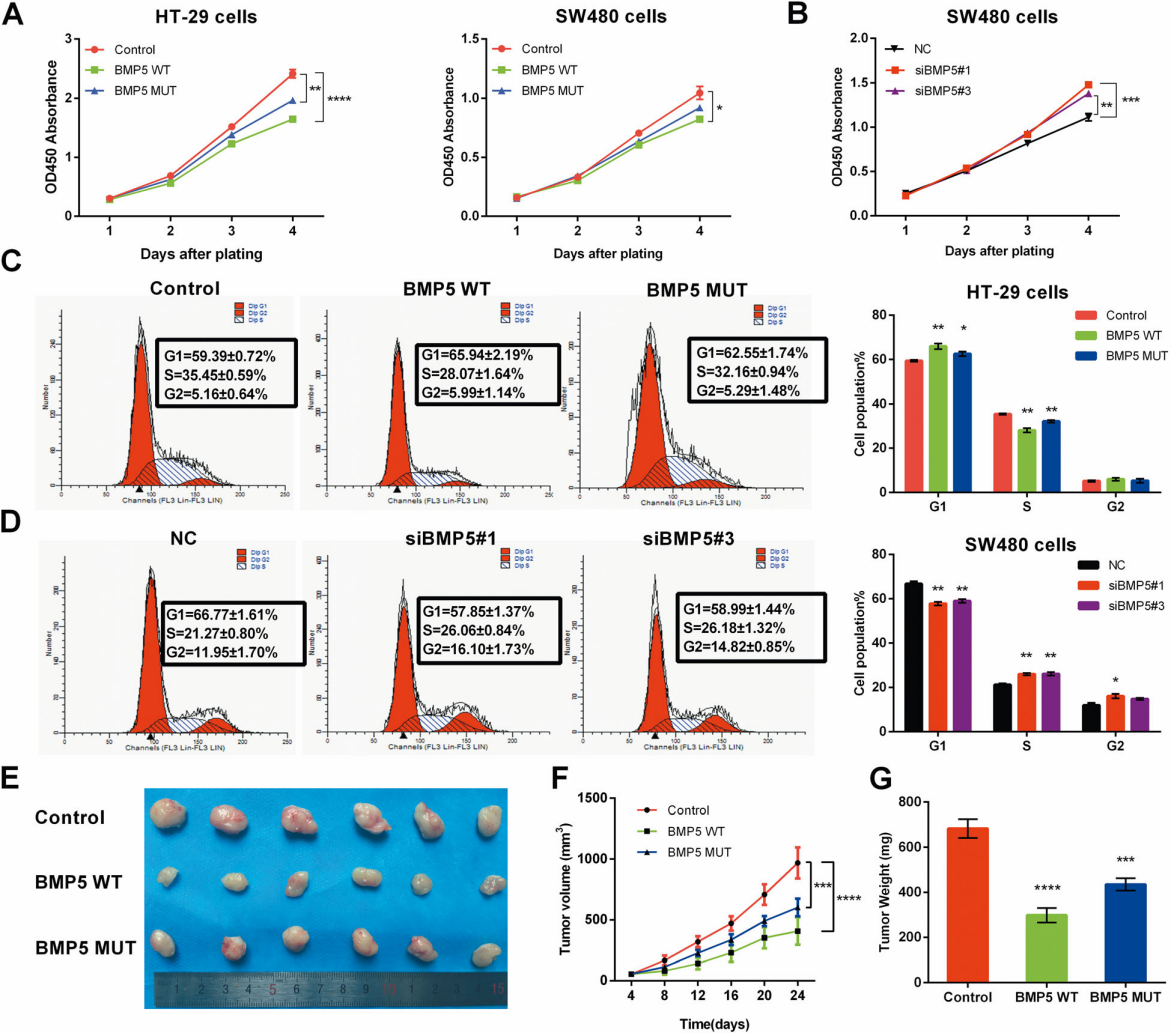
BMP5 inhibits cell proliferation in CRC model. **a-b** HT-29 and SW480 cell proliferation was determined by CCK8 assays after transfection, on day 1, 2, 3, 4. **c-d** Cell cycle distributions of HT-29 and SW480 cells were detected by flow cytometry. Representative of triplicate experiments was shown. **e** Images of isolated tumors from injected nude mice. **f** Tumor volume was assessed every 4 days with a total period of 25 days (*n* = 6). **g** Weights of the isolated tumor (*n* = 6). All data are showed as mean ± sem. *P < 0.05, **P < 0.01, ***P < 0.001, ****P < 0.0001

As previous studies reported that BMP5 inhibits cell proliferation in several tumor entities [15–17], we investigated the influence of BMP5 on colorectal cancer cell growth. BMP5 overexpression and knockdown efficiency were validated by western blots (Additional file 2: Figure S7). Both WT and MUT type BMP5 could decrease cell growth rate than control (pEF-BOS-EX vector-transfected cells) (HT-29 cells: WT *P* < 0.0001, MUT *P < 0.01*. SW480 cells: WT *P* < 0.05. Fig. 4a). while knockdown of BMP5 in SW480 cells promoted cell proliferation (Fig. 4b). The blots of tumor proliferation biomarker PCNA confirmed BMP5 could affect HT-29 and SW480 cell growth (Additional file 2: Figure S7). Flow cytometry showed cell growth inhibition effect of BMP5 is mainly mediated by cell cycle alterations. BMP5 could promote G1 phase cell cycle arrest (Fig. 4c-d) but had no effect on CRC cell apoptosis (Additional file 2: Figure S8). To evaluate the effect of BMP5 on tumorigenicity in vivo, we engineered HT-29 cells (BMP5-deficient cells) to stably express WT or MUT BMP5 by lentiviral infection and puromycin selection. Implant of HT-29 cells in mice revealed that expression of WT BMP5 reduced the tumor size and tumor weight as compared to BMP5-deficient control group (*P* < 0.0001), whereas the tumor inhibition effect weakened in MUT BMP5 group though the difference is statistically significant (*P* < 0.001) (Fig. 4e-g).

### BMP5 suppresses migration, invasion, and modulates epithelial-mesenchymal transition in CRC cells

**Fig. 5:**
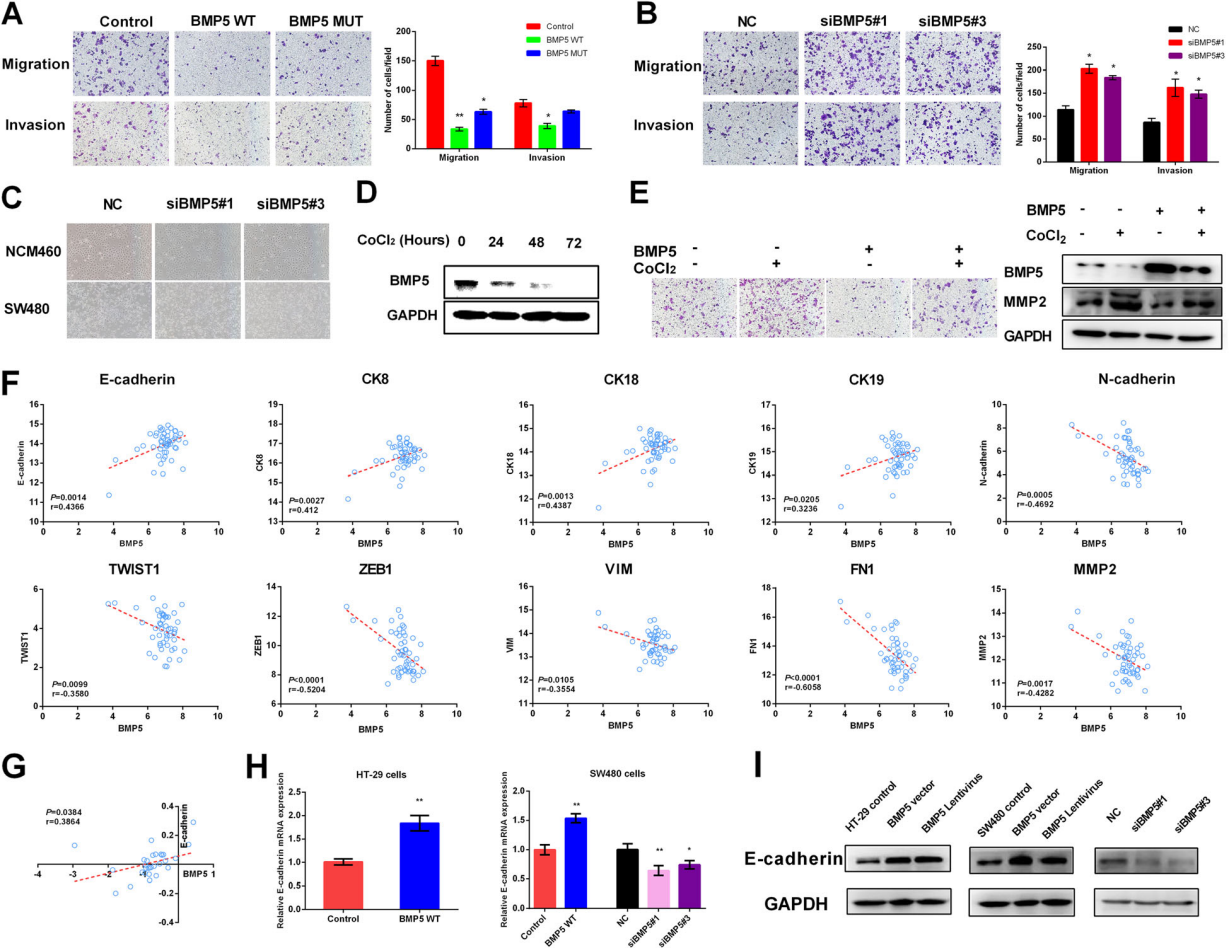
BMP5 supresses migration, invasion, and modulates EMT in CRC. **a-b** The migration and invasion ability was assessed using Transwell chamber (48 h after transfection for SW480 cells). Representative images were visualized at a magnification of 100×, *P < 0.05, **P < 0.01. **c** Cell morphological changes in epithelial NCM460 and SW480 cells after knockdown of BMP5. **D** Western blot showed BMP5 was downregulated in a time course after addition of CoCl_2_ in SW480 cells. **e** BMP5 expression could attenuate SW480 cell motility ability induced by CoCl_2_ mediated EMT. BMP5 and MMP2 expression were validated by blotting. **f** Pearson correlation between BMP5 and EMT markers in normal colorectal tissue of TCGA samples. **g** Correlation of fold change (Log_2_(Tumor/Normal)) between BMP5 and E-cadherin. **h** E-cadherin mRNA level analysis after transfection of BMP5 WT vector or siRNA, *P < 0.05, **P < 0.01. **i** E-cadherin protein level analysis after transfection of BMP5 WT vector or infection of BMP5 lentivirus in HT-29 and SW480 cells, as well as siRNA-mediated knockdown in SW480 cells

As reported previously, BMP5 has effect on cell migration and invasion, but the results were controversial [17, 18]. In SW480 cells, both WT BMP5 and MUT BMP5 led to reduced cell migration, whereas MUT BMP5 failed to suppress invasion (Fig. 5a; WT: *P* < 0.01, MUT: *P* < 0.05 for migration, and WT: *P* < 0.05, MUT: *P* = 0.25 for invasion, paired t-test). Furthermore, knockdown of BMP5 by two siRNAs significantly promoted both cell migration and invasion in SW480 cells (Fig. 5b). Migration indicator MMP2 showed inhibition of BMP5 enhanced the ability of cell mobility (Additional file 2: Figure S7).

Increasing evidence has confirmed the vital role of epithelial-mesenchymal transition (EMT) process in tumor initiation and metastasis [19]. Cell phenotypes change during the EMT process. Firstly, we investigated whether loss of BMP5 could promote cell motile abilities. Silencing of BMP5 indeed switched the epithelial cells NCM460 and SW480 from a polygonal and condensed pattern to a spindle and scattering like pattern (Fig. 5c). Addition of cobalt chloride (CoCl_2_) could induce EMT in vitro, causing loss of E-cadherin and increased level of ZEB and TWIST [20], and we then asked whether the expression of BMP5 changed during this process. In our study, BMP5 was downregulated in a time course after addition of CoCl_2_ in SW480 cells (Fig. 5d). Functionally, BMP5 expression could attenuate SW480 cell motility ability induced by CoCl_2_ (Fig. 5e).

In clinical samples, we found BMP5 expression was positively correlated with epithelial markers, and negatively correlated with mesenchymal markers (Fig. 5f and Additional file 2: Figure S9A). Further analysis in paired samples found only the fold change (Log_2_(Tumor/Normal)) of E-cadherin was correlated with that of BMP5 (Fig. 5g and Additional file 2: Figure S9B). What is more, we did not find this correlation in other 6 solid tumors tested (Additional file 2: Figure S9C). In HT-29 and SW480 cells, both overexpression and siRNA-mediated inhibition of BMP5 could affect E-cadherin expression (Fig. 5h and i). E-cadherin plays an essential role in cell-cell adhesion in normal cells, and the coexpression pattern of BMP5 and E-cadherin may reflect the importance of BMP5 in maintaining normal epithelial cell homeostasis, which confirms our hypothesis that loss of BMP5 is an early event in CRC.

### A novel downstream network of BMP5 determined by RNA sequencing

**Fig. 6:**
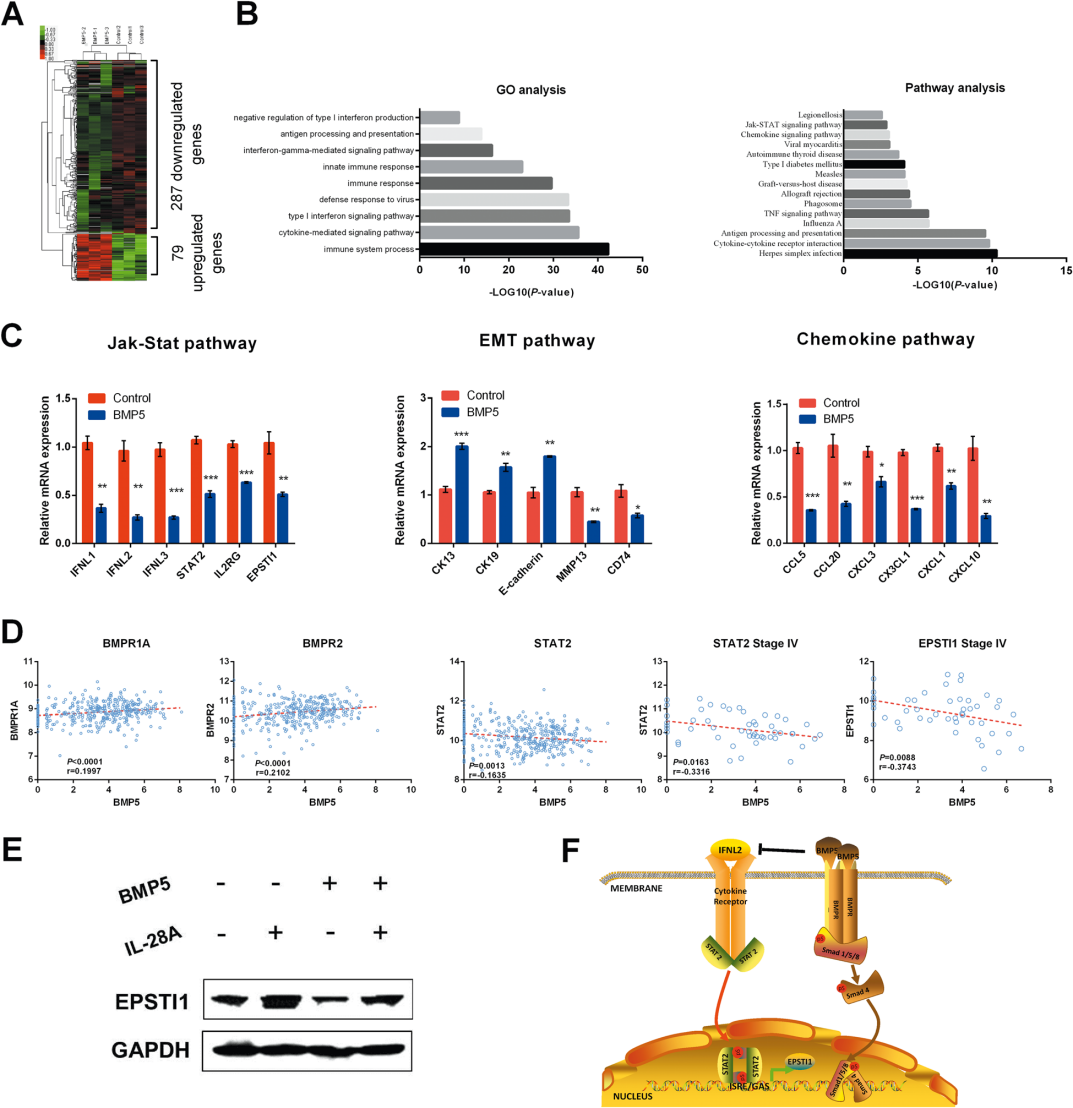
Transcriptome profile identifies novel downstream genes and pathways induced by BMP5. **a** The global expression profile of HT-29 and BMP5 expressing HT-29 cells was analyzed by RNA-seq analysis. The heat map shows the differentially gene expression patterns (fold change > 1.5 or < 0.667). **b** (Left) GO analysis of differentially expressed genes. (Right) Pathway analysis of differentially expressed genes. A Fisher exact test was used to find the significant enrichment GO term or pathway. The resulting *P* values were adjusted using the BH FDR algorithm. **c** Differentially expressed genes in Jak-Stat signaling pathway, EMT, and chemokine pathway identified in RNA-seq were confirmed by qPCR. **d** BMP5 was positively correlated with its receptors BMPR1A and BMPR2, and negatively correlated with STAT2 (*P* = 0.0013, *r* = − 0.1635) in all CRC samples. In Stage IV samples, the correlation was stronger for both STAT2 (*P* = 0.0163, *r* = − 0.3316) and EPSTI1 (*P* = 0.0088, *r* = − 0.3743). **e** IL-28A can activate EPSTI1 expression, while BMP5 can inhibit EPSTI1 expression. Addition of BMP5 can attenuate the activation of EPSTI1 induced by IL-28A in HT-29 cells. **f** Schematic model of BMP5-induced inhibition of Jak-Stat signaling pathway

In this study, we sought to characterize the downstream gene and pathway changes induced by BMP5. Transcriptome-wide analysis found 79 upregulated and 287 downregulated genes, with ≥1.5-fold change as compared to control group (Fig. 6a and Additional file 1: Table S14). The differentially expressed genes (DEGs) are mainly on interferons, interferon-stimulated genes (ISGs), chemokines and some EMT markers, which enriched in cytokine-cytokine receptor interaction, TNF signaling, chemokine signaling, and Jak-Stat signaling pathway (Fig. 6b and c). Interestingly, genes involved in BMP/Smad signaling pathway were not differentially expressed in this study, indicating BMP5 may signal through Smad-independent pathways. We further presented gene coexpression networks to reveal the interactions among genes (Additional file 1: Table S15 and Additional file 2 Figure S10). *EPSTI1* (Epithelial Stromal Interaction 1), an interferon stimulated gene, is the core regulatory gene in BMP5-expressing HT-29 cells. We also found IL-28A (IFNL2), the type III interferon, can signal through the Jak-Stat pathway to activate EPSTI1 expression [21]. In this study, genes involved in Jak-Stat signaling pathway, including IFNL1, IFNL2, IFNL3, IL2RG, IL15RA, IL23A, STAT2 and CSF3 were downregulated by overexpression of BMP5.

We utilized TCGA database and found BMP5 was positively correlated with its receptor, BMPR1A, and BMPR2 (Fig. 6d), but showed no correlation with downstream effector Smad2, Smad3, and Smad4. However, in lung, breast, liver, and pancreatic cancer, BMP5 could correlated with at least one or more Smad effectors (Additional file 2: Figure S11). In CRC, BMP5 was negatively correlated with STAT2, and EPSTI1, especially in Stage IV samples (Fig. 6d). Together with the coexpression network, we found a novel pathway that BMP5 signals through. Expression of BMP5 inhibits interferons, especially IL28A, thus to block the activation of STATs. Phosphorylated STAT dimers bind to STAT-responsive elements in the promoter of *EPSTI1*, resulting in modulation of its transcription (Fig. 6e-f).

## Discussion

In this study, we uncovered for the first time, the characteristic of BMP5 genetic alteration in CRC is distinctive, and loss of BMP5 expression may be a CRC-specific event. (1) Amplification of copy number could be found in all tumor types tested except CRC, and truncating mutation is predominant in CRC. (2) IHC of tissue microarrays revealed loss of BMP5 is a CRC-specific event. While BMP5 positive rate is significantly higher in breast carcinoma than normal tissues, which is consistent with the copy number amplification tested in public data (Fig. 1e). This difference in genetic alteration, especially in that of colon and breast cancer may suggest that the role of BMP5 varies among tumors. Loss of BMP5 is predominant in CRC, indicating it may be an early event of normal epithelium-adenoma transition. Copy number aberrations (CNAs) are acquired gradually and sequentially over extended periods of time, leading to successively more malignant stages of cancer. In breast cancer, amplification of BMP5 may act as a passenger, thus promote the advanced tumor progression. (3) mRNA level of BMP5 showed significant decreased in tumors than adjacent normal tissues in TCGA cohort and our validation group. Low expression of BMP5 correlates with patients’ poor survival, which is not significant in other 6 tumor types.

Next generation sequencing (NGS) has been in developing over the past ten years, leading to the identification of hundreds of high-penetrance mutation genes in different types of complex diseases or Mendelian diseases [22–27]. Geneticaly, the core of tumorigenesis is the alteration in driver genes, which ultimately leads to the emergence of a tumor phenotype. Genetic variants predicted to severely disrupt protein-coding genes, known as loss-of-function variants are rare and deleterious [10]. In this study, we performed exome analysis of 3 sCRC tumor samples with LoF mutation screening strategy, together with systems-level analysis, we uncovered well-studied tumor suppressor *APC, TCF7L2,* and *ELF3* in CRC, as well as novel top rank gene *BMP5* that can affect CRC initiation, progression and prognosis.

Several studies have demonstrated that BMP5 functions as tumor suppressor in myeloma, adrenocortical carcinoma, and breast cancer [15, 16, 18]. However, for gastrointestinal cancer and lung cancer that with high incidence and mortality rate worldwide, the intensive study of BMP5 has not been investigated yet. In CRC, we found decreased cell growth is mainly mediated by cell cycle, consistent with the previous study [17]. Our RNA-seq results also showed CDKN1C, a tight-binding inhibitor of several G1 cyclin/Cdk complexes and a negative regulator of cell proliferation, is upregulated after transfection of BMP5 (Additional file 1: Table S14), leading to a significant G1-S phase arrest. EMT is an essential step in metastatic cascades and characterized by loss of epithelial marker and increased level of mesenchymal markers. In BMP5-expressing HT-29 cells, we found upregulation of epithelial marker CK13, CK19, and E-cadherin, as compared with BMP5-deficient HT-29 cells. Cell motility markers, such as MMPs and CCL chemokines were downregulated.

Although BMP5 belongs to TGF-β/Smad signaling pathway, the downstream network induced by BMP5 is far from clear. Our transcriptome sequencing results discovered a novel pathway BMP5 transduces through, the Jak-STAT signaling. BMP5 may play as a negative regulator in this network. Core gene *EPSTI1* found by coexpression analysis convinced us the relationship between BMP5 and Jak-Stat pathway, and the CRC initiation role BMP5 plays in. *EPSTI1*, mapped to chromosome 13q13.3, is initially reported as a stromal fibroblast-induced gene in human breast cancer and highly upregulated in tumor tissues of breast cancer patients [28]. Expression levels of EPSTI1 associate with tumor initiation, stem cell–like properties, and EMT. The increased level of EPSTI1 is involved in activation of Jak-Stat pathway induced by IL28A, and BMP5 negatively suppress IL28A expression thus to inhibit the genes downstream of this pathway.

## Conclusions

In summary, the finding of common alterations in BMP5, together with functional data indicating its effect on cell growth and migration, suggest that BMP5 is an important tumor suppressor in human CRC. Particularly important is the fact that loss of BMP5 is an early event in CRC, and its prognostic value and coexpression pattern with E-cadherin could be sCRC tissue-specific. We also found a BMPs/Smad-independent pathway BMP5 may participate in, that suppress the expression of EPSTI1 through Jak-Stat signaling pathway induced by IL28A. Our results help further understand the importance of BMPs in CRC initiation and development.

## Additional files

Additional file 1: **Table S1.** Sample details. **Table S2.** BMP5 primer sequences. **Table S3.** qPCR Primers used in this study. **Table S4.** Tissue microarray sample details. **Table S5.** Primers used in BMP5 cloning. **Table S6.** BMP5 siRNAs used in this study. **Table S7.** Somatic loss of function mutations identified in exome sequencing. **Table S8.** 71 Gene expression analysis in normal and tumor samples. **Table S9.** Gene expression analysis in 3 GEO datasets. **Table S10.** Kaplan-Meier survival analysis of 71 genes in CRC (Data from HPA database using best seperation). **Table S11.** Gene function and pathway annotation. **Table S12.** Correlation of BMP5 expression to age, gender and clinical grade of 129 caddolorectal adenocarcinomas. **Table S13.** Correlation of BMP5 expression to age, gender, BMI, and recurrence of TCGA CRC samples. **Table S14.** Differentially expressed genes (Control vs BMP5 transfected in HT-29 cells). **Table S15.** Coexpression network analysis. (DOC 2017 kb)

Additional file 2: **Figure S1.** Exome capture and sequencing of 3 sporadic colorectal cancer samples. **Figure S2.** Detection of BMP5 somatic mutations in initial exome sequencing and expanded deep sequencing samples. **Figure S3.** Alignment of BMP5 in different species. The nonsynonymous mutation are marked and indicated with arrows, all seven amino acids are highly conserved among different species. Sus: *Sus scrofa*, Dr.: Zebra fish. **Figure S4.** Kaplan–Meier analysis of overall survival according to high or low BMP5 expression in six tumor types (Log rank test). **Figure S5.** Immunofluorescence staining of BMP5 in NCM460 (A) and SW480 (B) cells. **Figure S6.** Pearson correlation analysis between BMP5 and miR-32, miR-655. The data were analyzed using TCGA CRC tumor samples and stage IV samples. **Figure S7.** Overexpression and knockdown efficiency validation in HT-29 and SW480 cells. (A) Overexpression efficiency of BMP5 in HT-29 cells. Tumor proliferation marker PCNA was checked. (B) Overexpression and knockdown efficiency of BMP5 in SW480. Tumor proliferation marker PCNA and migration marker (MMP2 and MMP9) were detected. MMP9 showed no significant difference. **Figure S8.** Cell apoptosis of BMP5 in HT-29 and SW480 cells. The data are showed as mean ± sem. **Figure S9.** Pearson correlation analysis between BMP5 and EMT markers. (A) Correlation analysis in tumor samples. (B)Correlation of fold change (Log_2_(Tumor/Normal)) between BMP5 and EMT markers. (C) Correlation between BMP5 and E-cadherin in six tumor types. **Figure S10.** Coexpression network of control HT-29 (A) and BMP5-expressing HT-29 cells (B). **Figure S11.** Correlation analysis between BMP5 and SMAD or STAT signaling in lung, breast, esophagus, stomach, liver and pancreatic cancer. (DOC 10825 kb)

## Abbreviations

BMP5: Bone morphogenetic protein 5
CRC: colorectal cancer
EMT: epithelial-mesenchymal transition
GEO: Gene Expression Omnibus
HPA: Human Protein Atlas
KEGG: Kyoto Encyclopedia of Genes and Genomes
LoF: loss of function
MSI: Microsatellite instability
sCRC: Sporadic colorectal cancer
TCGA: The Cancer Genome Atlas
