# Supplementary material for: Alteration of tumor suppressor BMP5 in sporadic colorectal cancer: a genomic and transcriptomic profiling based study
Source: Mol Cancer. 2018 Dec 20;17:176. doi: 10.1186/s12943-018-0925-7 (PMC6302470; doi:10.1186/s12943-018-0925-7)
Supplement: Supplementary file 1 — Table S1. Sample details. Table S2. BMP5 primer sequences. Table S3. qPCR Primers used in this study. Table S4. Tissue microarray sample details. Table S5. Primers used in BMP5 cloning. Table S6. BMP5 siRNAs used in this study. Table S7. Somatic loss of function mutations identified in exome sequencing. Table S8. 71 Gene expression analysis in normal and tumor samples. Table S9. Gene expression analysis in 3 GEO datasets. Table S10. Kaplan-Meier survival analysis of 71 genes in CRC (Data from HPA database using best seperation). Table S11. Gene function and pathway annotation. Table S12. Correlation of BMP5 expression to age, gender and clinical grade of 129 caddolorectal adenocarcinomas. Table S13. Correlation of BMP5 expression to age, gender, BMI, and recurrence of TCGA CRC samples. Table S14. Differentially expressed genes (Control vs BMP5 transfected in HT-29 cells). Table S15. Coexpression network analysis. (DOC 2017 kb) [file 12943_2018_925_MOESM1_ESM.doc]

**Supplementary Tables**

**Table S1: Sample details**

| **Sample** | **Age** | **Gender** | **Grade** | **Location** | **Matched normal source** |
| --- | --- | --- | --- | --- | --- |
| In-1 | 52 | M | I-II | rectum | Blood |
| In-2 | 74 | F | I | colon ascendens | Blood |
| In-3 | 67 | F | I-II | right hemicolon | Blood |
| Ex-1 | 71 | M | I-II | rectum | Adjacent normal tissue |
| Ex-2 | 66 | F | II | colon sigmoideum | Adjacent normal tissue |
| Ex-3 | 45 | M | II | colon | Adjacent normal tissue |
| Ex-4 | 54 | F | II | rectum | Adjacent normal tissue |
| Ex-5 | 39 | M | I | rectum | Adjacent normal tissue |
| Ex-6 | 65 | F | II | rectum | Adjacent normal tissue |
| Ex-7 | 71 | F | II | [colon transversum](http://www.baidu.com/link?url=TlqQzNuEcmImcDriV0zGVfQv-sqGYhEoI0xvjMpFm3UcJ9jB8imv6eienosRK-5OouqcDj75TA7jgr2weFav0rACfir-j4xBPGgmHwQxCus5fzIXBR2fI43Fnfh-mwHJ) | Adjacent normal tissue |
| Ex-8 | 80 | M | II | [colon transversum](http://www.baidu.com/link?url=TlqQzNuEcmImcDriV0zGVfQv-sqGYhEoI0xvjMpFm3UcJ9jB8imv6eienosRK-5OouqcDj75TA7jgr2weFav0rACfir-j4xBPGgmHwQxCus5fzIXBR2fI43Fnfh-mwHJ) | Adjacent normal tissue |
| Ex-9 | 73 | M | II | rectum | Adjacent normal tissue |
| Ex-10 | 66 | F | II | right hemicolon | Adjacent normal tissue |
| Ex-11 | 64 | F | II | rectum | Adjacent normal tissue |
| Ex-12 | 78 | M | II | ileocecus | Adjacent normal tissue |
| Ex-13 | 42 | M | I-II | colon sigmoideum | Adjacent normal tissue |
| Ex-14 | 55 | F | III | rectum | Adjacent normal tissue |
| Ex-15 | 39 | M | II | [colon transversum](http://www.baidu.com/link?url=TlqQzNuEcmImcDriV0zGVfQv-sqGYhEoI0xvjMpFm3UcJ9jB8imv6eienosRK-5OouqcDj75TA7jgr2weFav0rACfir-j4xBPGgmHwQxCus5fzIXBR2fI43Fnfh-mwHJ) | Adjacent normal tissue |
| Ex-16 | 80 | F | II | colon sigmoideum | Adjacent normal tissue |
| Ex-17 | 63 | M | II | colon | Adjacent normal tissue |
| Ex-18 | 53 | F | I-II | rectum | Adjacent normal tissue |
| Ex-19 | 54 | F | II | rectum | Adjacent normal tissue |
| Ex-20 | 52 | F | II | rectum | Adjacent normal tissue |
| Ex-21 | 77 | F | I-II | rectum | Adjacent normal tissue |
| Ex-22 | 83 | M | - | rectal squamous cell | Adjacent normal tissue |
| Ex-23 | 38 | F | II | colon sigmoideum | Adjacent normal tissue |
| Ex-24 | 42 | M | II | colon ascendens | Adjacent normal tissue |
| Ex-25 | 77 | F | II | right hemicolon | Adjacent normal tissue |
| Ex-26 | 73 | M | II | rectum | Adjacent normal tissue |
| Ex-27 | 78 | F | - | [colon transversum](http://www.baidu.com/link?url=TlqQzNuEcmImcDriV0zGVfQv-sqGYhEoI0xvjMpFm3UcJ9jB8imv6eienosRK-5OouqcDj75TA7jgr2weFav0rACfir-j4xBPGgmHwQxCus5fzIXBR2fI43Fnfh-mwHJ) | Adjacent normal tissue |
| Ex-28 | 67 | F | - | colon sigmoideum | Adjacent normal tissue |
| Ex-29 | 71 | F | I | colon | Adjacent normal tissue |
| Ex-30 | 58 | M | II | right hemicolon | Adjacent normal tissue |
| Ex-31 | 60 | F | II | colon ascendens | Adjacent normal tissue |
| Ex-32 | 62 | F | I-II | colon sigmoideum | Adjacent normal tissue |
| Ex-33 | 55 | M | II | colon ascendens | Adjacent normal tissue |
| Ex-34 | 52 | F | I-II | rectum | Adjacent normal tissue |
| Ex-35 | 47 | M | - | rectal squamous cell | Adjacent normal tissue |
| Ex-36 | 62 | M | II | colon sigmoideum | Adjacent normal tissue |
| Ex-37 | 57 | F | II | rectum | Adjacent normal tissue |
| Ex-38 | 81 | F | II | rectum | Adjacent normal tissue |
| Ex-39 | 78 | M | II-III | rectum | Adjacent normal tissue |
| Ex-40 | 76 | M | II | colon ascendens | Adjacent normal tissue |
| Ex-41 | 52 | F | II | colon descendens | Adjacent normal tissue |
| Ex-42 | 79 | F | II | colon sigmoideum | Adjacent normal tissue |
| Ex-43 | 67 | F | II | ileocecus | Adjacent normal tissue |
| Ex-44 | 79 | M | II | colon sigmoideum | Adjacent normal tissue |
| Ex-45 | 58 | M | I-II | colon | Adjacent normal tissue |
| Ex-46 | 49 | M | II | rectum | Adjacent normal tissue |
| Ex-47 | 73 | M | II | right hemicolon | Adjacent normal tissue |
| Ex-48 | 52 | F | II | [colon transversum](http://www.baidu.com/link?url=TlqQzNuEcmImcDriV0zGVfQv-sqGYhEoI0xvjMpFm3UcJ9jB8imv6eienosRK-5OouqcDj75TA7jgr2weFav0rACfir-j4xBPGgmHwQxCus5fzIXBR2fI43Fnfh-mwHJ) | Adjacent normal tissue |
| Ex-49 | 48 | F | II | colon sigmoideum | Adjacent normal tissue |
| Ex-50 | 69 | M | II | colon ascendens | Adjacent normal tissue |
| Ex-51 | 73 | F | I-II | rectum | Adjacent normal tissue |
| Ex-52 | 74 | M | II | rectum | Adjacent normal tissue |
| Ex-53 | 52 | F | II | rectum | Adjacent normal tissue |
| Ex-54 | 75 | F | II | left hemicolon | Adjacent normal tissue |
| Ex-55 | 81 | M | II | colon | Adjacent normal tissue |
| Ex-56 | 78 | F | II-III | colon sigmoideum | Adjacent normal tissue |
| Ex-57 | 68 | M | II | rectum | Adjacent normal tissue |
| Ex-58 | 67 | F | II-III | colon sigmoideum | Adjacent normal tissue |
| Ex-59 | 52 | F | I-II | rectum | Adjacent normal tissue |
| Ex-60 | 63 | M | I | rectum | Adjacent normal tissue |
| Ex-61 | 63 | F | II | colon sigmoideum | Adjacent normal tissue |
| Ex-62 | 71 | F | II | right hemicolon | Adjacent normal tissue |
| Ex-63 | 50 | F | II | colon descendens | Adjacent normal tissue |
| Ex-64 | 55 | F | II | rectum | Adjacent normal tissue |
| Ex-65 | 63 | M | II | rectum | Adjacent normal tissue |
| Ex-66 | 68 | F | II | colon sigmoideum | Adjacent normal tissue |
| Ex-67 | 77 | M | II | colon sigmoideum and rectum | Adjacent normal tissue |
| Ex-68 | 57 | F | II | rectum | Adjacent normal tissue |
| Ex-69 | 61 | F | II | rectum | Adjacent normal tissue |
| Ex-70 | 61 | M | II | rectum | Adjacent normal tissue |
| Ex-71 | 63 | M | II-III | rectum | Adjacent normal tissue |
| Ex-72 | 72 | F | II | rectum | Adjacent normal tissue |
| Ex-73 | 67 | M | II | rectum | Adjacent normal tissue |
| Ex-74 | 79 | F | I-II | [colon transversum](http://www.baidu.com/link?url=TlqQzNuEcmImcDriV0zGVfQv-sqGYhEoI0xvjMpFm3UcJ9jB8imv6eienosRK-5OouqcDj75TA7jgr2weFav0rACfir-j4xBPGgmHwQxCus5fzIXBR2fI43Fnfh-mwHJ) | Adjacent normal tissue |
| Ex-75 | 73 | M | I-II | right hemicolon | Adjacent normal tissue |
| Ex-76 | 44 | F | I-II | rectum | Adjacent normal tissue |
| Ex-77 | 65 | M | II | rectum | Adjacent normal tissue |
| Ex-78 | 53 | F | II | rectum | Adjacent normal tissue |
| Ex-79 | 46 | F | I-II | right hemicolon | Adjacent normal tissue |
| Ex-80 | 63 | M | II | colon | Adjacent normal tissue |
| Ex-81 | 65 | F | II | rectum | Adjacent normal tissue |
| Ex-82 | 53 | F | II | colon | Adjacent normal tissue |
| Ex-83 | 81 | F | II | rectum | Adjacent normal tissue |
| Ex-84 | 78 | F | II | colon sigmoideum | Adjacent normal tissue |
| Ex-85 | 75 | M | II | colon sigmoideum | Adjacent normal tissue |
| Ex-86 | 70 | M | II | colon | Adjacent normal tissue |
| Ex-87 | 60 | M | I | rectum | Adjacent normal tissue |
| Ex-88 | 48 | M | II | colon | Adjacent normal tissue |
| Ex-89 | 80 | M | II | colon | Adjacent normal tissue |
| Ex-90 | 65 | F | II | colon | Adjacent normal tissue |
| Ex-91 | 75 | M | II | rectum | Adjacent normal tissue |
| Ex-92 | 56 | F | II | rectum | Adjacent normal tissue |
| Ex-93 | 83 | M | II | colon sigmoideum | Adjacent normal tissue |
| Ex-94 | 74 | F | II | ileocecus | Adjacent normal tissue |
| Ex-95 | 73 | F | II | colon | Adjacent normal tissue |
| Ex-96 | 83 | M | II | rectum | Adjacent normal tissue |
| Ex-97 | 55 | M | II | colon | Adjacent normal tissue |
| Ex-98 | 52 | M | II | colon | Adjacent normal tissue |
| Ex-99 | 81 | M | II | rectum | Adjacent normal tissue |
| Ex-100 | 57 | F | II | colon sigmoideum | Adjacent normal tissue |
| Ex-101 | 57 | M | II | colon | Adjacent normal tissue |
| R-1 | 67 | M | I | NA | Adjacent normal tissue |
| R-2 | 44 | F | II | NA | Adjacent normal tissue |
| R-3 | 81 | M | II | NA | Adjacent normal tissue |
| R-4 | 51 | M | II | NA | Adjacent normal tissue |
| R-5 | 65 | F | II | NA | Adjacent normal tissue |
| R-6 | 57 | M | III | NA | Adjacent normal tissue |
| R-7 | 61 | M | II | NA | Adjacent normal tissue |
| R-8 | 68 | M | II | NA | Adjacent normal tissue |
| R-9 | 62 | M | II | NA | Adjacent normal tissue |
| R-10 | 78 | M | II | NA | Adjacent normal tissue |
| R-11 | 73 | M | II | NA | Adjacent normal tissue |
| R-12 | 76 | F | II | NA | Adjacent normal tissue |
| R-13 | 31 | F | II | NA | Adjacent normal tissue |
| R-14 | 78 | F | III | NA | Adjacent normal tissue |
| R-15 | 55 | M | II | NA | Adjacent normal tissue |
| R-16 | 55 | F | II | NA | Adjacent normal tissue |
| R-17 | 47 | F | II | NA | Adjacent normal tissue |
| R-18 | 63 | M | II | NA | Adjacent normal tissue |
| R-19 | 49 | M | II | NA | Adjacent normal tissue |
| R-20 | 45 | M | II | NA | Adjacent normal tissue |
| R-21 | 61 | F | II | NA | Adjacent normal tissue |
| R-22 | 72 | M | II | NA | Adjacent normal tissue |
| R-23 | 74 | F | III | NA | Adjacent normal tissue |
| R-24 | 76 | F | II | NA | Adjacent normal tissue |
| R-25 | 64 | F | II | NA | Adjacent normal tissue |
| R-26 | 71 | M | III | NA | Adjacent normal tissue |
| R-27 | 73 | F | II | NA | Adjacent normal tissue |
| R-28 | 64 | F | II | NA | Adjacent normal tissue |

**Table S2**: BMP5 primer sequences

| **Exon** | **Product length** | **Amplification primers（5'-3'）** | **Sequencing primer（5'-3'）** |
| --- | --- | --- | --- |
| Exon 1-1 | 357 bp | F：AAGAGGACAAGAAGGACTAAA | TGCCCTTACTGAGTACTCCG |
|  |  | R：TGCCCTTACTGAGTACTCCG |  |
| Exon 1-2 | 323 bp | F：TTTGCCTCACAGACCCAGAC | TTTGCCTCACAGACCCAGAC |
|  |  | R：TGAACAACATACCTAAGTTGAC |  |
| Exon 2 | 357 bp | F：AAAGCTGGAGAATTTATCATTA | ATAGATCACATGAGTTATCAA |
|  |  | R：ATAGATCACATGAGTTATCAA |  |
| Exon 3 | 351 bp | F：AGGTTTGGTTGGGAGCCTAG | AGGTTTGGTTGGGAGCCTAG |
|  |  | R：TCAGTGCTTACCATCCCCTG |  |
| Exon 4 | 353 bp | F：AACCAAGTGCTTTTGTATTA | AAACATCACTGGAAGATTTAAT |
|  |  | R：AAACATCACTGGAAGATTTAAT |  |
| Exon 5 | 319 bp | F：TCCACAACTTCAAATAAAACCCC | AGCCATATTCTAAATGGTCATGT |
|  |  | R：AGCCATATTCTAAATGGTCATGT |  |
| Exon 6 | 277 bp | F：GTCAGGACTGGATTATAGCA | TGACTATACCCACAGTTGTT |
|  |  | R：TGACTATACCCACAGTTGTT |  |
| Exon 7 | 355 bp | F：GGTGTGCCTGATTATGAGTGA | CCCCGTTTGTCTGAAAGTATGC |
|  |  | R：CCCCGTTTGTCTGAAAGTATGC |  |

**Table S3: qPCR P**rimers used in this study

| **Gene** | **Product length** | **Forward primer** | **Reverse primer** |
| --- | --- | --- | --- |
| BMP5 | 60 bp | GCCTGTAAGAAGCACGAAC | AATCCAGTCCTGCCATCCC |
| CCL5 | 54 bp | CCAGCAGTCGTCTTTGTCAC | CTCTGGGTTGGCACACACTT |
| CCL20 | 138 bp | GACCGTATTCTTCATCCTA | TTTCACCCAAGTCTGTTT |
| CD74 | 206 bp | CCGGCTGGACAAACTGACA | GGTGCATCACATGGTCCTCTG |
| CK13 | 139 bp | CCCCAGGCATTGACCTGAC | GTGTTGGTAGACACCTCCTTG |
| CK19 | 98 bp | TGAGTGACATGCGAAGCCAAT | CTCCCGGTTCAATTCTTCAGTC |
| CXCL1 | 111 bp | CCCCAAGAACATCCAAAGTG | GATGCAGGATTGAGGCAAG |
| CXCL3 | 109 bp | GCCCAAACCGAAGTCATA | TGCTCCCCTTGTTCAGTATC |
| CXCL10 | 93 bp | GCCATTCTGATTTGCT | AATGCTGATGCAGGTA |
| CX3CL1 | 83 bp | ACTATCAACAGAACCAGGCATCA | CGGGTCGGCACAGAACA |
| CDH1 | 119 bp | CGAGAGCTACACGTTCACGG | GGGTGTCGAGGGAAAAATAGG |
| EPSTI1 | 206 bp | ACCCGCAATAGAGTGGTGAAC | GCTATCAAGGTGTATGCACTTGT |
| GAPDH | 86 bp | TGGAAATCCCATCACCATCT | TGGACTCCACGACGTACTCA |
| IFNL1 | 85 bp | GTGGTGCTGGTGACTTTGGTG | GGCAGCCCTTCCCAGTTGT |
| IFNL2 | 183 bp | CAAAGATGCCTTAGAAGAGTCGC | TCCACCAGGGCTGGGTCAGT |
| IFNL3 | 74 bp | CCAGTTCAAGTCCCTGTCTCCA | GAAGCGACTCTTCTAAGGCATCTTT |
| IL2RG | 107 bp | TGGGATTGATTATCAGCCTTCT | CCCGTGGTATTCAGTAACAAGA |
| MMP13 | 137 bp | CCAGACTTCACGATGGCATTG | GGCATCTCCTCCATAATTTGGC |
| STAT2 | 87 bp | CTGCTAGGCCGATTAACTACCC | TCTGATGCAGGCTTTTTGCTG |

**Table S4: Tissue microarray sample details**

| **Sample** | **Age** | **Gender** | **Grade** | **Organ** | **Normal tissue staining** | **Tumor tissue staining** |
| --- | --- | --- | --- | --- | --- | --- |
| C1 | 67 | F | II | colon | 2+ | 1+ |
| C2 | 62 | F | II | colon | 1+ | 1+ |
| C3 | 45 | F | I | colon | 3+ | 2+ |
| C4 | 57 | M | I | colon | 2+ | 1+ |
| C5 | 45 | M | II | colon | 2+ | 1+ |
| C6 | 32 | F | II | colon | 1+ | 1+ |
| C7 | 58 | F | II | colon | 2+ | 1+ |
| C8 | 39 | M | I | colon | 2+ | 2+ |
| C9 | 72 | F | II | colon | 3+ | 3+ |
| C10 | 50 | M | II | colon | 3+ | 2+ |
| C11 | 80 | M | I | colon | 2+ | 2+ |
| C12 | 81 | M | I | colon | 1+ | 2+ |
| C13 | 62 | F | I | colon | 2+ | 2+ |
| C14 | 62 | F | II | colon | 3+ | 3+ |
| C15 | 43 | M | II | colon | 1+ | 2+ |
| C16 | 64 | M | II | colon | 1+ | 1+ |
| C17 | 90 | M | I | colon | 1+ | 1+ |
| C18 | 78 | M | I | colon | 2+ | 1+ |
| C19 | 60 | M | II | colon | 3+ | 2+ |
| C20 | 77 | F | I | colon | 1+ | 2+ |
| C21 | 52 | M | II | colon | 1+ | 1+ |
| C22 | 60 | M | II | colon | 1+ | 1+ |
| C23 | 60 | M | II | colon | 1+ | 1+ |
| C24 | 32 | M | II | colon | 2+ | 1+ |
| C25 | 60 | M | III | colon | 0 | 0 |
| C26 | 57 | M | II | colon | 1+ | 0 |
| C27 | 73 | M | II | colon | 1+ | 1+ |
| C28 | 46 | F | II | colon | 1+ | 1+ |
| C29 | 57 | F | II | colon | 2+ | 1+ |
| C30 | 64 | M | I | colon | 0 | 0 |
| C31 | 52 | F | III | colon | 0 | 0 |
| C32 | 51 | M | I | colon | 1+ | 1+ |
| C33 | 74 | M | II | colon | 2+ | 0 |
| C34 | 48 | M | I | colon | 0 | 0 |
| C35 | 55 | M | I | colon | 0 | 0 |
| C36 | 59 | M | II | colon | 1+ | 0 |
| C37 | 46 | M | II | colon | 1+ | 0 |
| C38 | 43 | M | II | colon | 1+ | 0 |
| C39 | 55 | M | I | colon | 2+ | 1+ |
| C40 | 55 | F | I | colon | 2+ | 2+ |
| C41 | 50 | M | II | colon | 3+ | 3+ |
| C42 | 60 | F | II | colon | 3+ | 3+ |
| C43 | 56 | M | II | colon | 2+ | 2+ |
| C44 | 64 | M | II | colon | 3+ | 2+ |
| C45 | 72 | F | II | colon | 3+ | 2+ |
| C46 | 45 | M | II | colon | 3+ | 3+ |
| C47 | 35 | F | II | colon | 2+ | 3+ |
| C48 | 56 | F | II | colon | 2+ | 2+ |
| C49 | 31 | M | II | colon | 2+ | 2+ |
| C50 | 63 | F | II | colon | 1+ | 2+ |
| C51 | 73 | M | II | colon | 1+ | 1+ |
| C52 | 66 | M | II | colon | 3+ | 0 |
| C53 | 48 | M | II | colon | 2+ | 2+ |
| C54 | 72 | M | II | colon | 2+ | 2+ |
| C55 | 48 | M | II | colon | 2+ | 3+ |
| C56 | 54 | M | II | colon | 2+ | 1+ |
| C57 | 45 | F | II | colon | 1+ | 2+ |
| C58 | 49 | M | II | colon | 2+ | 1+ |
| C59 | 54 | M | II | colon | 3+ | 2+ |
| C60 | 65 | M | II | colon | 3+ | 2+ |
| C61 | 69 | M | II | colon | 2+ | 0 |
| C62 | 76 | M | II | colon | 1+ | 1+ |
| C63 | 39 | F | II | colon | 2+ | 1+ |
| C64 | 33 | M | II | colon | 1+ | 2+ |
| C65 | 59 | M | II | colon | 1+ | 1+ |
| C66 | 78 | M | II | colon | 1+ | 0 |
| C67 | 37 | F | III | colon | 1+ | 2+ |
| C68 | 58 | M | III | colon | 1+ | 0 |
| C69 | 58 | M | III | colon | 1+ | 1+ |
| C70 | 74 | M | III | colon | 1+ | 0 |
| C71 | 53 | M | III | colon | 1+ | 1+ |
| C72 | 38 | F | III | colon | 3+ | 2+ |
| C73 | 61 | F | II | colon | 3+ | 3+ |
| C74 | 55 | M | III | colon | 3+ | 3+ |
| C75 | 60 | F | III | colon | 3+ | 3+ |
| C76 | 61 | F | III | colon | 3+ | 3+ |
| C77 | 62 | M | II | colon | 3+ | 2+ |
| C78 | 60 | F | I | colon | 3+ | 2+ |
| C79 | 70 | F | III | colon | 3+ | 2+ |
| C80 | 60 | F | II | colon | 3+ | 1+ |
| C81 | 55 | M | II | colon | 2+ | 1+ |
| C82 | 38 | M | III | colon | 3+ | 1+ |
| C83 | 70 | M | II | colon | 2+ | 1+ |
| C84 | 74 | F | II | colon | 2+ | 2+ |
| C85 | 65 | F | II | colon | 2+ | 1+ |
| C86 | 41 | F | II | colon | 2+ | 1+ |
| C87 | 38 | F | III | colon | 2+ | 1+ |
| C88 | 55 | M | II | colon | 2+ | 1+ |
| C89 | 34 | M | II | colon | 2+ | 1+ |
| C90 | 40 | F | II | colon | 2+ | 1+ |
| C91 | 54 | F | II | colon | 2+ | 2+ |
| C92 | 82 | F | II | colon | 2+ | 1+ |
| C93 | 58 | F | III | colon | 2+ | 1+ |
| C94 | 49 | M | II | colon | 1+ | 1+ |
| C95 | 62 | M | III | colon | 1+ | 0 |
| C96 | 34 | F | III | colon | 1+ | 1+ |
| C97 | 40 | F | II | colon | 1+ | 0 |
| C98 | 73 | F | II | colon | 1+ | 1+ |
| C99 | 65 | F | II | colon | 2+ | 1+ |
| C100 | 67 | M | I | colon | 1+ | 1+ |
| C101 | 37 | M | II | colon | 1+ | 1+ |
| C102 | 54 | F | II | colon | 1+ | 0 |
| C103 | 70 | F | III | colon | 1+ | 0 |
| C104 | 74 | F | III | colon | 1+ | 0 |
| C105 | 44 | M | II | colon | 1+ | 0 |
| C106 | 31 | M | I | colon | 1+ | 0 |
| C107 | 52 | F | II | colon | 1+ | 0 |
| C108 | 48 | M | I | colon | 1+ | 0 |
| C109 | 32 | M | II | colon | 1+ | 0 |
| C110 | 56 | F | II | colon | 2+ | 2+ |
| C111 | 73 | F | III | colon | 1+ | 1+ |
| C112 | 45 | M | II | colon | 1+ | 0 |
| C113 | 79 | M | II | colon | 0 | 0 |
| C114 | 35 | M | III | colon | 1+ | 0 |
| C115 | 67 | F | III | colon | 1+ | 0 |
| C116 | 65 | M | II | colon | 2+ | 1+ |
| C117 | 75 | M | II | colon | 1+ | 0 |
| C118 | 74 | M | II | colon | 1+ | 1+ |
| C119 | 60 | M | II | colon | 0 | 0 |
| C120 | 57 | M | II | colon | 1+ | 1+ |
| C121 | 57 | F | II | colon | 1+ | 0 |
| C122 | 74 | M | II | colon | 0 | 0 |
| C123 | 70 | M | II | colon | 1+ | 0 |
| C124 | 68 | F | II | colon | 0 | 0 |
| C125 | 28 | F | II | colon | 0 | 0 |
| C126 | 55 | M | II | colon | 0 | 0 |
| C127 | 61 | F | II | colon | 0 | 0 |
| C128 | 41 | M | III | colon | 0 | 0 |
| C129 | 51 | M | II | colon | 1+ | 0 |
| B1 | 38 | F | III | Breast | 0 | 3+ |
| B2 | 38 | F | II | Breast | 0 | 0 |
| B3 | 55 | F | II | Breast | 0 | 0 |
| B4 | 49 | F | II | Breast | 0 | 0 |
| B5 | 58 | F | II | Breast | 0 | 1+ |
| B6 | 30 | F | II | Breast | 0 | 1+ |
| B7 | 51 | F | II | Breast | 0 | 0 |
| B8 | 78 | F | II | Breast | 0 | 0 |
| B9 | 38 | F | I | Breast | 1+ | 1+ |
| B10 | 35 | F | II | Breast | 0 | 1+ |
| B11 | 47 | F | II | Breast | 0 | 1+ |
| B12 | 45 | F | I | Breast | 0 | 1+ |
| B13 | 41 | F | II | Breast | 0 | 1+ |
| B14 | 30 | F | II | Breast | 0 | 1+ |
| B15 | 38 | F | II | Breast | 0 | 1+ |
| B16 | 34 | F | II | Breast | 0 | 1+ |
| B17 | 40 | F | I | Breast | 0 | 1+ |
| B18 | 52 | F | III | Breast | 0 | 1+ |
| B19 | 40 | F | I | Breast | 1+ | 1+ |
| B20 | 49 | F | II | Breast | 0 | 1+ |
| B21 | 36 | F | II | Breast | 0 | 1+ |
| B22 | 36 | F | I | Breast | 0 | 0 |
| B23 | 37 | F | I | Breast | 0 | 0 |
| B24 | 46 | F | I | Breast | 1+ | 1+ |
| B25 | 56 | F | II | Breast | 0 | 1+ |
| B26 | 52 | F | II | Breast | 0 | 0 |
| B27 | 36 | F | I | Breast | 0 | 0 |
| B28 | 46 | F | II | Breast | 0 | 0 |
| B29 | 51 | F | II | Breast | 1+ | 1+ |
| B30 | 32 | F | II | Breast | 0 | 1+ |
| B31 | 32 | F | II | Breast | 0 | 0 |
| B32 | 30 | F | I | Breast | 0 | 0 |
| B33 | 38 | F | I | Breast | 0 | 1+ |
| B34 | 48 | F | I | Breast | 0 | 0 |
| B35 | 40 | F | II | Breast | 0 | 0 |
| B36 | 56 | F | II | Breast | 0 | 0 |
| B37 | 39 | F | II | Breast | 0 | 0 |
| B38 | 50 | F | II | Breast | 1+ | 0 |
| B39 | 53 | F | II | Breast | 0 | 0 |
| B40 | 31 | F | II | Breast | 0 | 0 |
| E1 | 55 | F | I | Esophagus | 2+ | 3+ |
| E2 | 51 | F | I | Esophagus | 1+ | 3+ |
| E3 | 49 | M | I | Esophagus | 2+ | 3+ |
| E4 | 50 | M | I | Esophagus | 2+ | 3+ |
| E5 | 58 | M | I | Esophagus | 0 | 0 |
| E6 | 55 | M | I | Esophagus | 2+ | 3+ |
| E7 | 58 | M | I | Esophagus | 2+ | 1+ |
| E8 | 48 | M | I | Esophagus | 3+ | 2+ |
| E9 | 66 | M | I | Esophagus | 3+ | 2+ |
| E10 | 56 | M | I | Esophagus | 3+ | 1+ |
| E11 | 59 | F | I | Esophagus | 2+ | 3+ |
| E12 | 58 | M | II | Esophagus | 1+ | 3+ |
| E13 | 56 | M | II | Esophagus | 0 | 1+ |
| E14 | 64 | M | I | Esophagus | 1+ | 1+ |
| E15 | 60 | M | II | Esophagus | 0 | 0 |
| E16 | 53 | M | I | Esophagus | 2+ | 3+ |
| E17 | 55 | F | I | Esophagus | 2+ | 3+ |
| E18 | 54 | M | I | Esophagus | 2+ | 1+ |
| E19 | 46 | M | II | Esophagus | 2+ | 1+ |
| E20 | 60 | M | III | Esophagus | 2+ | 1+ |
| E21 | 36 | M | III | Esophagus | 3+ | 1+ |
| E22 | 65 | F | III | Esophagus | 3+ | 1+ |
| E23 | 69 | F | III | Esophagus | 2+ | 1+ |
| E24 | 63 | F | I | Esophagus | 2+ | 1+ |
| E25 | 57 | F | III | Esophagus | 1+ | 0 |
| E26 | 52 | M | III | Esophagus | 3+ | 2+ |
| E27 | 60 | F | III | Esophagus | 1+ | 3+ |
| E28 | 60 | M | III | Esophagus | 1+ | 2+ |
| E29 | 61 | M | III | Esophagus | 1+ | 0 |
| E30 | 55 | M | II | Esophagus | 2+ | 1+ |
| E31 | 73 | F | II | Esophagus | 2+ | 2+ |
| E32 | 52 | M | II | Esophagus | 1+ | 2+ |
| E33 | 41 | M | II | Esophagus | 1+ | 2+ |
| E34 | 63 | M | II | Esophagus | 1+ | 0 |
| E35 | 48 | M | II | Esophagus | 1+ | 0 |
| E36 | 66 | M | III | Esophagus | 1+ | 1+ |
| E37 | 57 | M | II | Esophagus | 2+ | 2+ |
| E38 | 64 | M | III | Esophagus | 0 | 0 |
| E39 | 60 | M | III | Esophagus | 1+ | 2+ |
| E40 | 66 | M | II | Esophagus | 1+ | 1+ |
| E41 | 68 | F | III | Esophagus | 1+ | 2+ |
| E42 | 58 | M | II | Esophagus | 1+ | 2+ |
| E43 | 45 | M | II | Esophagus | 1+ | 1+ |
| E44 | 53 | F | III | Esophagus | 0 | 1+ |
| E45 | 49 | F | III | Esophagus | 0 | 1+ |
| E46 | 54 | F | III | Esophagus | 1+ | 1+ |
| E47 | 45 | M | II | Esophagus | 0 | 1+ |
| E48 | 48 | M | III | Esophagus | 0 | 1+ |
| E49 | 66 | M | II | Esophagus | 0 | 0 |
| E50 | 58 | M | II | Esophagus | 0 | 0 |
| LV1 | 66 | M | I | Liver | 2+ | 3+ |
| LV2 | 52 | M | I | Liver | 2+ | 1+ |
| LV3 | 46 | M | I | Liver | 1+ | 1+ |
| LV4 | 60 | M | II | Liver | 1+ | 1+ |
| LV5 | 45 | M | II | Liver | 2+ | 2+ |
| LV6 | 36 | F | II | Liver | 2+ | 3+ |
| LV7 | 35 | M | II | Liver | 2+ | 2+ |
| LV8 | 48 | M | II | Liver | 3+ | 2+ |
| LV9 | 48 | M | II | Liver | 1+ | 3+ |
| LV10 | 37 | F | II | Liver | 2+ | 1+ |
| LV11 | 52 | F | II | Liver | 2+ | 3+ |
| LV12 | 32 | F | II | Liver | 3+ | 2+ |
| LV13 | 55 | F | II | Liver | 1+ | 1+ |
| LV14 | 43 | M | II | Liver | 2+ | 3+ |
| LV15 | 48 | M | II | Liver | 2+ | 1+ |
| LV16 | 43 | M | II | Liver | 1+ | 3+ |
| LV17 | 52 | M | II | Liver | 2+ | 2+ |
| LV18 | 43 | F | II | Liver | 1+ | 2+ |
| LV19 | 71 | M | II | Liver | 2+ | 3+ |
| LV20 | 36 | M | II | Liver | 2+ | 1+ |
| LV21 | 40 | M | II | Liver | 2+ | 3+ |
| LV22 | 45 | F | II | Liver | 2+ | 2+ |
| LV23 | 48 | M | III | Liver | 1+ | 1+ |
| LV24 | 56 | M | III | Liver | 2+ | 3+ |
| LV25 | 49 | F | II | Liver | 2+ | 1+ |
| LV26 | 46 | M | II | Liver | 2+ | 3+ |
| LV27 | 48 | M | II | Liver | 2+ | 3+ |
| LV28 | 52 | M | III | Liver | 2+ | 3+ |
| LV29 | 39 | M | II | Liver | 2+ | 2+ |
| LV30 | 48 | M | II | Liver | 3+ | 2+ |
| LV31 | 56 | M | II | Liver | 2+ | 1+ |
| LV32 | 41 | M | II | Liver | 2+ | 2+ |
| LV33 | 57 | M | II | Liver | 2+ | 1+ |
| LV34 | 62 | F | II | Liver | 1+ | 2+ |
| LV35 | 67 | M | II | Liver | 2+ | 1+ |
| LV36 | 54 | M | II | Liver | 1+ | 2+ |
| LV37 | 57 | M | II | Liver | 1+ | 2+ |
| LV38 | 38 | M | II | Liver | 1+ | 3+ |
| LV39 | 50 | M | II | Liver | 2+ | 2+ |
| LV40 | 43 | F | II | Liver | 2+ | 2+ |
| LV41 | 47 | M | II | Liver | 1+ | 3+ |
| LV42 | 37 | M | I | Liver | 2+ | 3+ |
| LV43 | 50 | M | II | Liver | 1+ | 2+ |
| LV44 | 50 | F | III | Liver | 2+ | 2+ |
| LV45 | 51 | M | III | Liver | 2+ | 2+ |
| LV46 | 63 | M | II | Liver | 1+ | 2+ |
| LV47 | 45 | M | II | Liver | 1+ | 1+ |
| LV48 | 40 | M | II | Liver | 2+ | 3+ |
| LV49 | 40 | F | II | Liver | 2+ | 3+ |
| LV50 | 70 | F | III | Liver | 2+ | 2+ |
| LN1 | 69 | F | II | Lung | 0 | 0 |
| LN2 | 65 | M | II | Lung | 0 | 0 |
| LN3 | 72 | M | II | Lung | 0 | 0 |
| LN4 | 54 | F | II | Lung | 0 | 0 |
| LN5 | 75 | M | II | Lung | 0 | 0 |
| LN6 | 59 | F | II | Lung | 0 | 0 |
| LN7 | 68 | M | II | Lung | 0 | 0 |
| LN8 | 68 | M | II | Lung | 0 | 0 |
| LN9 | 62 | F | II | Lung | 0 | 0 |
| LN10 | 37 | F | II | Lung | 0 | 0 |
| LN11 | 52 | M | II | Lung | 0 | 0 |
| LN12 | 65 | M | II | Lung | 0 | 0 |
| LN13 | 49 | F | II | Lung | 0 | 0 |
| LN14 | 75 | M | III | Lung | 0 | 0 |
| LN15 | 46 | M | II | Lung | 0 | 0 |
| LN16 | 53 | M | II | Lung | 0 | 0 |
| LN17 | 62 | M | II | Lung | 0 | 0 |
| LN18 | 53 | M | II | Lung | 0 | 0 |
| LN19 | 66 | F | II | Lung | 0 | 0 |
| LN20 | 62 | F | II | Lung | 0 | 0 |
| LN21 | 39 | M | II | Lung | 0 | 0 |
| LN22 | 59 | F | II | Lung | 0 | 0 |
| LN23 | 52 | F | II | Lung | 0 | 0 |
| LN24 | 53 | M | II | Lung | 0 | 0 |
| LN25 | 59 | M | III | Lung | 0 | 0 |
| LN26 | 62 | M | III | Lung | 0 | 0 |
| LN27 | 62 | M | II | Lung | 0 | 0 |
| LN28 | 37 | F | II | Lung | 0 | 0 |
| LN29 | 55 | M | III | Lung | 0 | 0 |
| LN30 | 63 | F | II | Lung | 0 | 0 |
| LN31 | 65 | M | III | Lung | 0 | 0 |
| LN32 | 64 | M | I | Lung | 0 | 0 |
| LN33 | 40 | M | I | Lung | 0 | 0 |
| LN34 | 69 | M | III | Lung | 0 | 0 |
| LN35 | 51 | M | II | Lung | 0 | 0 |
| LN36 | 50 | M | III | Lung | 0 | 0 |
| LN37 | 65 | M | III | Lung | 0 | 0 |
| LN38 | 30 | M | III | Lung | 0 | 0 |
| LN39 | 54 | F | III | Lung | 0 | 0 |
| LN40 | 36 | F | III | Lung | 0 | 0 |
| LN41 | 77 | M | III | Lung | 0 | 0 |
| LN42 | 63 | M | III | Lung | 0 | 0 |
| LN43 | 32 | F | III | Lung | 0 | 0 |
| LN44 | 51 | F | III | Lung | 0 | 0 |
| LN45 | 61 | M | III | Lung | 0 | 0 |
| LN46 | 52 | F | III | Lung | 0 | 0 |
| LN47 | 52 | M | III | Lung | 0 | 0 |
| LN48 | 60 | M | III | Lung | 0 | 0 |
| LN49 | 63 | M | III | Lung | 0 | 0 |
| LN50 | 52 | M | III | Lung | 0 | 0 |
| P1 | 38 | F | I | Pancreas | 0 | 2+ |
| P2 | 74 | M | II | Pancreas | 0 | 0 |
| P3 | 47 | M | I | Pancreas | 2+ | 0 |
| P4 | 40 | M | I | Pancreas | 0 | 0 |
| P5 | 54 | M | I | Pancreas | 2+ | 2+ |
| P6 | 64 | F | I | Pancreas | 2+ | 3+ |
| P7 | 80 | M | II | Pancreas | 0 | 0 |
| P8 | 68 | F | II | Pancreas | 0 | 0 |
| P9 | 62 | F | II | Pancreas | 0 | 0 |
| P10 | 49 | M | II | Pancreas | 2+ | 0 |
| P11 | 56 | F | I | Pancreas | 3+ | 3+ |
| P12 | 61 | M | II | Pancreas | 1+ | 2+ |
| P13 | 52 | M | II | Pancreas | 0 | 1+ |
| P14 | 46 | F | II | Pancreas | 0 | 0 |
| P15 | 64 | M | II | Pancreas | 1+ | 2+ |
| P16 | 48 | F | II | Pancreas | 2+ | 2+ |
| P17 | 76 | F | III | Pancreas | 1+ | 1+ |
| P18 | 65 | M | II | Pancreas | 2+ | 2+ |
| P19 | 64 | M | II | Pancreas | 0 | 2+ |
| P20 | 66 | F | I | Pancreas | 1+ | 0 |
| P21 | 65 | M | II | Pancreas | 1+ | 2+ |
| P22 | 68 | F | II | Pancreas | 0 | 1+ |
| P23 | 60 | M | II | Pancreas | 2+ | 2+ |
| P24 | 60 | M | III | Pancreas | 1+ | 1+ |
| P25 | 60 | M | II | Pancreas | 1+ | 1+ |
| P26 | 58 | F | I | Pancreas | 1+ | 3+ |
| P27 | 71 | F | III | Pancreas | 1+ | 3+ |
| P28 | 51 | M | III | Pancreas | 1+ | 1+ |
| P29 | 47 | F | III | Pancreas | 1+ | 1+ |
| P30 | 51 | F | I | Pancreas | 1+ | 2+ |
| P31 | 54 | M | II | Pancreas | 1+ | 3+ |
| P32 | 56 | M | III | Pancreas | 0 | 0 |
| P33 | 62 | M | III | Pancreas | 1+ | 0 |
| P34 | 53 | F | III | Pancreas | 0 | 0 |
| P35 | 78 | M | III | Pancreas | 0 | 0 |
| P36 | 48 | F | III | Pancreas | 3+ | 3+ |
| P37 | 66 | F | III | Pancreas | 0 | 2+ |
| P38 | 55 | F | III | Pancreas | 0 | 0 |
| P39 | 45 | M | I | Pancreas | 0 | 0 |
| P40 | 62 | M | II | Pancreas | 0 | 0 |
| P41 | 48 | F | NA | Pancreas | 2+ | 2+ |
| P42 | 31 | M | NA | Pancreas | 0 | 2+ |
| P43 | 51 | F | NA | Pancreas | 0 | 0 |
| P44 | 51 | M | NA | Pancreas | 0 | 0 |
| P45 | 52 | M | III | Pancreas | 0 | 0 |
| P46 | 47 | M | NA | Pancreas | 3+ | 3+ |
| P47 | 50 | M | NA | Pancreas | 0 | 2+ |
| P48 | 21 | F | NA | Pancreas | 0 | 0 |
| P49 | 27 | M | NA | Pancreas | 0 | 0 |
| P50 | 40 | M | NA | Pancreas | 0 | 0 |
| G1 | 53 | M | I | Stomach | 1+ | 2+ |
| G2 | 55 | M | I | Stomach | 1+ | 2+ |
| G3 | 80 | F | II | Stomach | 1+ | 2+ |
| G4 | 75 | M | II | Stomach | 1+ | 2+ |
| G5 | 65 | M | II | Stomach | 1+ | 0 |
| G6 | 61 | M | II | Stomach | 1+ | 3+ |
| G7 | 58 | M | II | Stomach | 1+ | 2+ |
| G8 | 76 | M | II | Stomach | 3+ | 1+ |
| G9 | 70 | M | II | Stomach | 1+ | 1+ |
| G10 | 70 | M | III | Stomach | 1+ | 0 |
| G11 | 49 | M | II | Stomach | 1+ | 3+ |
| G12 | 65 | M | II | Stomach | 1+ | 1+ |
| G13 | 53 | M | II | Stomach | 0 | 1+ |
| G14 | 66 | M | I | Stomach | 1+ | 1+ |
| G15 | 68 | M | II | Stomach | 1+ | 1+ |
| G16 | 61 | F | II | Stomach | 1+ | 2+ |
| G17 | 60 | F | II | Stomach | 1+ | 2+ |
| G18 | 69 | M | II | Stomach | 1+ | 1+ |
| G19 | 77 | M | II | Stomach | 1+ | 1+ |
| G20 | 56 | M | II | Stomach | 1+ | 1+ |
| G21 | 72 | M | II | Stomach | 1+ | 2+ |
| G22 | 64 | M | III | Stomach | 1+ | 2+ |
| G23 | 44 | M | II | Stomach | 1+ | 1+ |
| G24 | 64 | M | II | Stomach | 1+ | 2+ |
| G25 | 63 | M | III | Stomach | 1+ | 1+ |
| G26 | 55 | F | III | Stomach | 1+ | 1+ |
| G27 | 59 | M | III | Stomach | 1+ | 2+ |
| G28 | 57 | M | II | Stomach | 1+ | 1+ |
| G29 | 39 | F | III | Stomach | 1+ | 2+ |
| G30 | 60 | M | III | Stomach | 1+ | 2+ |
| G31 | 55 | F | II | Stomach | 1+ | 2+ |
| G32 | 57 | M | III | Stomach | 1+ | 1+ |
| G33 | 42 | M | III | Stomach | 1+ | 1+ |
| G34 | 37 | M | III | Stomach | 0 | 0 |
| G35 | 78 | F | III | Stomach | 1+ | 1+ |
| G36 | 58 | M | II | Stomach | 1+ | 1+ |
| G37 | 65 | M | III | Stomach | 1+ | 2+ |
| G38 | 74 | F | III | Stomach | 1+ | 1+ |
| G39 | 45 | M | III | Stomach | 0 | 0 |
| G40 | 48 | M | III | Stomach | 1+ | 2+ |
| G41 | 34 | F | III | Stomach | 1+ | 3+ |
| G42 | 60 | M | III | Stomach | 1+ | 1+ |
| G43 | 67 | M | III | Stomach | 1+ | 1+ |
| G44 | 30 | M | III | Stomach | 1+ | 1+ |
| G45 | 51 | M | III | Stomach | 0 | 1+ |
| G46 | 53 | M | III | Stomach | 1+ | 1+ |
| G47 | 73 | F | III | Stomach | 1+ | 1+ |
| G48 | 43 | F | III | Stomach | 1+ | 1+ |
| G49 | 62 | M | III | Stomach | 1+ | 1+ |
| G50 | 55 | F | III | Stomach | 1+ | 2+ |

**C: colorectal cancer; B: breast cancer; E: esophageal cancer; LV: liver cancer; LN: lung cancer; P: pancreatic cancer; G: gastric cancer.**

**Table S5: Primers used in BMP5 cloning**

| **Forward** | tttggatcccatctgactgtatttttacttaaggg |
| --- | --- |
| **Reverse** | tttgtcgacttagtggcagccacatgagcgtac |

**Table S6: BMP5 siRNAs used in this study**

| **siBMP5#1** | GAGUCGGAGUACUCAGUAA |
| --- | --- |
| **siBMP5#3** | CCAACCACGCUAUAGUUCA |

**Table S7: Somatic loss of function mutations identified in exome sequencing**

| **Sample** | **Gene** | **Annotated transcript** | **Chromosome** | **Position** | **Mutation type** |
| --- | --- | --- | --- | --- | --- |
| 1 | *BMP5* | ENST00000370830 | 6 | 55684616 | C>T, NONSENSE |
| 1 | *DDI2* | ENST00000480945 | 1 | 15970110 | Del T, FRAMESHIFT |
| 1 | *APC* | ENST00000457016 | 5 | 112175746 | Del T, FRAMESHIFT |
| 1 | *TCF7L2* | ENST00000545257 | 10 | 114920446 | Del T, FRAMESHIFT |
| 1 | *HFM1* | ENST00000370424 | 1 | 91727873 | INS T, FRAMESHIFT |
| 2 | *SYDE2* | ENST00000341460 | 1 | 85648161 | C>T, NONSENSE |
| 2 | *PHLDB2* | ENST00000359729 | 3 | 111603771 | C>T, NONSENSE |
| 2 | *KCNMB3* | ENST00000314235 | 3 | 178968622 | C>T, NONSENSE |
| 2 | *MEPE* | ENST00000395102 | 4 | 88755870 | C>T, NONSENSE |
| 2 | *PDE2A* | ENST00000475807 | 11 | 72301286 | G>T, NONSENSE |
| 2 | *REP15* | ENST00000310791 | 12 | 27850191 | T>A, NONSENSE |
| 2 | *DUOX2* | ENST00000389039 | 15 | 45392371 | C>T, NONSENSE |
| 2 | *CPPED1* | ENST00000261660 | 16 | 12758833 | C>T, NONSENSE |
| 2 | *ZNF404* | ENST00000324394 | 19 | 44377834 | C>T, NONSENSE |
| 2 | *TCP10L2* | ENST00000366832 | 6 | 167591956 | C>T, NONSENSE |
| 2 | *RFC2* | ENST00000055077 | 7 | 73651695 | C>A, NONSENSE |
| 2 | *DEM1* | ENST00000372703 | 1 | 40981008 | Del TG, FRAMESHIFT |
| 2 | *ELF3* | ENST00000310044 | 1 | 201982143 | INS G, FRAMESHIFT |
| 2 | *TMEM178* | ENST00000281961 | 2 | 39934211 | Del C, FRAMESHIFT |
| 2 | *LNP1* | ENST00000383693 | 3 | 100174674 | INS A, FRAMESHIFT |
| 2 | *TMPRSS7* | ENST00000437906 | 3 | 111766749 | Del C, FRAMESHIFT |
| 2 | *KIAA1407* | ENST00000483766 | 3 | 113753927 | INS A, FRAMESHIFT |
| 2 | *KIAA1407* | ENST00000545063 | 3 | 113697704 | G>A, SPLICE |
| 2 | *COPB2* | ENST00000507777 | 3 | 139097018 | INS T, FRAMESHIFT |
| 2 | *RIMS1* | ENST00000521978 | 6 | 72974697 | Del A, FRAMESHIFT |
| 2 | *AKD1* | ENST00000355283 | 6 | 109850200 | Del AC, FRAMESHIFT |
| 2 | *E2F5* | ENST00000518234 | 8 | 86121633 | INS ATAT, FRAMESHIFT |
| 2 | *LDB3* | ENST00000539402 | 10 | 88428502 | INS G, FRAMESHIFT |
| 2 | *TAS2R10* | ENST00000240619 | 12 | 10978289 | Del T, FRAMESHIFT |
| 2 | *ATP7B* | ENST00000400366 | 13 | 52539010 | Del C, FRAMESHIFT |
| 2 | *METTL21C* | ENST00000267273 | 13 | 103338590 | INS C, FRAMESHIFT |
| 2 | *ZNF527* | ENST00000317566 | 19 | 37879853 | INS TGTG, FRAMESHIFT |
| 2 | *ZNF175* | ENST00000262259 | 19 | 52084691 | INS G, FRAMESHIFT |
| 2 | *SPTLC3* | ENST00000399002 | 20 | 13052906 | Del T, FRAMESHIFT |
| 2 | *CTSA* | ENST00000372484 | 20 | 44520260 | Del TG, FRAMESHIFT |
| 2 | *SVEP1* | ENST00000401783 | 9 | 113220797 | INS G, FRAMESHIFT |
| 2 | *SSPO* | ENST00000262089 | 7 | 149479269 | G>C, SPLICE |
| 2 | *ATP6V0D2* | ENST00000285393 | 8 | 87163770 | G>T, SPLICE |
| 2 | *CAPN1* | ENST00000527189 | 11 | 64953386 | A>G, SPLICE |
| 3 | *POLR1A* | ENST00000409681 | 2 | 86281307 | C>T, NONSENSE |
| 3 | *EYS* | ENST00000503581 | 6 | 64431122 | C>A, NONSENSE |
| 3 | *ZNF3* | ENST00000303915 | 7 | 99669077 | G>T, NONSENSE |
| 3 | *IFT74* | ENST00000443698 | 9 | 27056428 | C>T, NONSENSE |
| 3 | *GTF3C5* | ENST00000372095 | 9 | 135930466 | C>T, NONSENSE |
| 3 | *AHNAK* | ENST00000257247 | 11 | 62259258 | C>T, NONSENSE |
| 3 | *EMR1* | ENST00000543519 | 19 | 6926385 | C>A, NONSENSE |
| 3 | *FBXW10* | ENST00000301938 | 17 | 18670128 | C>T, NONSENSE |
| 3 | *SLC16A4* | ENST00000472422 | 1 | 110906427 | Del A, FRAMESHIFT |
| 3 | *DYRK3* | ENST00000367108 | 1 | 206821525 | INS A, FRAMESHIFT |
| 3 | *C2orf81* | ENST00000290390 | 2 | 74641741 | Del C, FRAMESHIFT |
| 3 | *MGAT5* | ENST00000281923 | 2 | 135093823 | Del A, FRAMESHIFT |
| 3 | *CAPN10* | ENST00000391984 | 2 | 241536203 | Del G, FRAMESHIFT |
| 3 | *C3orf20* | ENST00000435614 | 3 | 14802989 | Del G, FRAMESHIFT |
| 3 | *DHX30* | ENST00000348968 | 3 | 47882535 | INS G, FRAMESHIFT |
| 3 | *CCDC54* | ENST00000261058 | 3 | 107097081 | Del AAATG, FRAMESHIFT |
| 3 | *LIMCH1* | ENST00000381753 | 4 | 41648508 | Del GA, FRAMESHIFT |
| 3 | *AMBN* | ENST00000538728 | 4 | 71468346 | Del AGG, FRAMESHIFT |
| 3 | *RASSF6* | ENST00000342081 | 4 | 74453572 | INS A, FRAMESHIFT |
| 3 | *DNAH5* | ENST00000265104 | 5 | 13769218 | Del G, FRAMESHIFT |
| 3 | *MLL5* | ENST00000311117 | 7 | 104730611 | Del A, FRAMESHIFT |
| 3 | *GPSM1* | ENST00000392945 | 9 | 139235482 | Del C, FRAMESHIFT |
| 3 | *SLCO1B3* | ENST00000261196 | 12 | 21069003 | INS T, FRAMESHIFT |
| 3 | *VSIG10* | ENST00000359236 | 12 | 118506329 | Del CCTC, FRAMESHIFT |
| 3 | *SLFN12L* | ENST00000260908 | 17 | 33806842 | Del G, FRAMESHIFT |
| 3 | *PRR11* | ENST00000262293 | 17 | 57247171 | Del A, FRAMESHIFT |
| 3 | *ATP8B1* | ENST00000283684 | 18 | 55365040 | INS T, FRAMESHIFT |
| 3 | *TMEM91* | ENST00000542945 | 19 | 41888851 | Del GGGGACTGGGC, FRAMESHIFT |
| 3 | *SPHK2* | ENST00000340932 | 19 | 49132157 | INS G, FRAMESHIFT |
| 3 | *C9orf9* | ENST00000356311 | 9 | 135754402 | G>A, SPLICE |
| 3 | *SARDH* | ENST00000422262 | 9 | 136535875 | G>T, SPLICE |
| 3 | *EZH2* | ENST00000541220 | 7 | 148507508 | A>T, SPLICE |
| 3 | *KRT73* | ENST00000546934 | 12 | 53004398 | G>A, SPLICE |

**Table S8: 71 Gene expression analysis in normal and tumor** samples

| **Gene** | **RNA Level** | | | | | **Protein Level (HPA)** | |
| --- | --- | --- | --- | --- | --- | --- | --- |
|  | **Normal colorectal tissue enriched(HPA)** | **Colon adenocarcinoma (TCGA)** | | **Rectum Adenocarcinoma (TCGA)** | | **Normal** | **Tumor** |
|  |  | ***P*Value** | **logFC** | ***P*Value** | **logFC** |  |  |
| *LDB3* |  | 4.77E-91 | -4.06548875 | NS |  | Not detected | Not detected |
| *PDE2A* |  | 4.47E-83 | -2.67178702 | 2.88E-36 | -2.93041909 | Low | Not detected |
| *ATP8B1* | Yes | 3.89E-66 | -1.66585801 | 1.52E-18 | -1.73474239 | High | Medium |
| *EZH2* |  | 9.56E-40 | 1.278283103 | 2.66E-08 | 1.201576271 | Medium | High |
| *ATP6V0D2* |  | 1.09E-33 | -2.22734403 | NS |  | Not detected | Not detected |
| *AHNAK* |  | 2.13E-32 | -1.55669236 | 2.11E-24 | -2.31999167 | Medium | Medium |
| *PRR11* | Yes | 1.03E-25 | 1.310670874 | 4.27E-05 | 1.190072845 | NA | Not detected/Low |
| *SVEP1* |  | 4.36E-25 | -1.72426276 | NS |  | Rectum: Medium Colon: Not detected | Not detected/Low |
| *BMP5* | Yes | 3.92E-24 | -2.61993003 | 3.35E-12 | -2.50703316 | NA | NA |
| *E2F5* |  | 1.18E-23 | 1.420551218 | 3.81E-06 | 1.250557121 | NA | NA |
| *SLCO1B3* |  | 1.78E-19 | 6.438470959 | 6.49E-06 | 6.983189352 | Not detected | Not detected/Low |
| *DNAH5* | Not detected | 7.15E-17 | 2.415360396 | 1.94E-04 | 2.309349805 | Not detected | Not detected |
| *RASSF6* | Yes | 2.24E-16 | -1.09016217 | 6.13E-06 | -1.15371031 | Medium | Not detected/Low |
| *REP15* | Yes | 1.70E-14 | -2.21349419 | 6.35E-06 | -2.43961443 | Rectum: Medium Colon: Low | Medium/Low |
| *SPTLC3* |  | 5.54E-09 | -1.07378605 | NS |  | Colon: High Rectum: Medium | Not detected |
| *PHLDB2* |  | 9.11E-08 | -1.01644695 | 6.01E-11 | -1.99563664 | Medium | High/Medium |
| *TMEM178* |  | 6.69E-05 | 1.525133888 | NS |  | NA | Not detected |
| *CAPN10* |  | NS |  | 3.32E-11 | 1.227997188 | Medium | Medium/Low |
| *APC* |  | NS |  | 2.27E-06 | -1.07634998 | Medium | Medium/Low |
| *ZNF404* |  | NS |  | 2.28E-04 | -1.18475565 | NA | Not detected/Low |
| *CPPED1* |  | NS |  | NS |  | Medium | Not detected/Low |
| *SLC16A4* |  | NS |  | NS |  | Medium | Not detected/Low |
| *ELF3* | Yes | NS |  | NS |  | High | Medium/Low |
| *GPSM1* |  | NS |  | NS |  | Medium | Not detected/Low |
| *TCF7L2* | Yes | NS |  | NS |  | High | High |
| *CTSA* | Yes | NS |  | NS |  | High | Medium |
| *VSIG10* | Yes | NS |  | NS |  | NA | NA |
| *SPHK2* |  | NS |  | NS |  | NA | NA |
| *MEPE* | Not detected | NS |  | NS |  | NA | Not detected/Low |
| *TMPRSS7* | Not detected | NS |  | NS |  | NA | Medium |
| *TAS2R10* | Not detected | NS |  | NS |  | NA | NA |
| *METTL21C* | Not detected | NS |  | NS |  | NA | NA |
| *SSPO* | Not detected | NS |  | NS |  | NA | NA |
| *FBXW10* | Not detected | NS |  | NS |  | NA | NA |
| *C3orf20* | Not detected | NS |  | NS |  | NA | Medium |
| *CCDC54* | Not detected | NS |  | NS |  | NA | NA |
| *AMBN* | Not detected | NS |  | NS |  | NA | NA |
| *KRT73* | Not detected | NS |  | NS |  | Not detected | Not detected |
| *SYDE2* |  | NS |  | NS |  | NA | Not detected |
| *DUOX2* |  | NS |  | NS |  | NA | NA |
| *RFC2* |  | NS |  | NS |  | Medium | Medium |
| *DEM1* |  | NS |  | NS |  | NA | NA |
| *ATP7B* |  | NS |  | NS |  | High | Midium/High |
| *COPB2* |  | NS |  | NS |  | High | Medium/High |
| *CAPN1* |  | NS |  | NS |  | High | High |
| *POLR1A* |  | NS |  | NS |  | Medium | Medium |
| *ZNF3* |  | NS |  | NS |  | High | High/Medium |
| *IFT74* |  | NS |  | NS |  | Medium | Medium |
| *GTF3C5* |  | NS |  | NS |  | High | Medium |
| *MGAT5* |  | NS |  | NS |  | Medium | Medium |
| *DHX30* |  | NS |  | NS |  | Medium | Medium/Low |
| *MLL5* |  | NS |  | NS |  | Not detected | Not detected |
| *DDI2* |  | NS |  | NS |  | Medium | Medium/Low |
| *HFM1* |  | NS |  | NS |  | NA | NA |
| *KCNMB3* |  | NS |  | NS |  | High | High/Medium |
| *TCP10L2* |  | NS |  | NS |  | NA | Not detected |
| *LNP1* |  | NS |  | NS |  | NA | NA |
| *KIAA1407* |  | NS |  | NS |  | Medium | Medium |
| *RIMS1* |  | NS |  | NS |  | NA | NA |
| *AKD1* |  | NS |  | NS |  | Medium | Medium |
| *ZNF527* |  | NS |  | NS |  | NA | Medium |
| *ZNF175* |  | NS |  | NS |  | NA | Low |
| *EYS* |  | NS |  | NS |  | Not detected | Not detected |
| *EMR1* |  | NS |  | NS |  | Low | NA |
| *DYRK3* |  | NS |  | NS |  | NA | NA |
| *C2orf81* |  | NS |  | NS |  | Rectum: Low Colon: Not detected | Not detected |
| *LIMCH1* |  | NS |  | NS |  | Low | Low |
| *SLFN12L* |  | NS |  | NS |  | NA | Low/Medium |
| *TMEM91* |  | NS |  | NS |  | NA | Not detected |
| *C9orf9* |  | NS |  | NS |  | Not detected | Not detected |
| *SARDH* |  | NS |  | NS |  | Medium | Medium/High |

**Table S9**：Gene expression analysis in 3 GEO datasets

| **Gene** | **GSE71187** | **GSE41258** | **GSE8671** |
| --- | --- | --- | --- |
| *BMP5* | 4.91E-08; down | - | 4.62E-17; down |
| *PDE2A* | 7.50E-16; down | 5.28E-28; down | 9.55E-15; down |
| *ZNF175* | 3.00E-04; down | - | 7.56E-03; down |
| *CTSA* | 1.12E-06; down | - | 1.71E-16; down |
| *SVEP1* | 1.45E-05; down | - | 8.15E-07; down |
| *ATP6V0D2* | 9.22E-22; down | - | 1.74E-06; down |
| *AHNAK* | 1.41E-05; down | - | 2.12E-12; down |
| *DUOX2* | 3.85E-12 | 2.85E-24; up | 4.04E-12; up |
| *POLR1A* | 1.88E-09; up | - | 2.17E-07; up |
| *SLC16A4* | 1.46E-07; up | - | 5.37E-12; up |
| *DNAH5* | 1.19E-07; up | - | 1.14E-04; up |
| *SLCO1B3* | 3.99E-22; up | 1.35E-15; up | 1.42E-17; up |
| *PRR11* | 8.41E-05; up | - | 6.77E-12; up |
| *DDI2* | 2.47E-04; up | - | - |
| *APC* | - | - | 8.45E-06; down |
| *HFM1* | 9.34E-08; up | - | 4.32E-02; down |
| *PHLDB2* | 3.02E-04; up | - | - |
| *MEPE* | 1.68E-06; up | - | - |
| *ZNF404* | - | - | 1.20E-03; down |
| *TMEM178* | 2.27E-05; up | - | 1.15E-07; down |
| *COPB2* | - | - | 6.83E-04; down |
| *RIMS1* | 2.17E-04; up | - | - |
| *LDB3* | 5.53E-04; up | - | 1.52E-02; down |
| *METTL21C* | 2.08E-05 | - | - |
| *SSPO* | 5.92E-08; up | - | - |
| *EYS* | 1.26E-04; up | - | - |
| *MGAT5* | 9.41E-07; up | - | - |
| *C3orf20* | 8.26E-08; up | - | - |
| *DHX30* | 4.26E-12; up | - | - |
| *CCDC54* | 7.37E-05; up | - | - |
| *LIMCH1* | 1.98E-02; up | - | - |
| *AMBN* | 2.43E-04; up | - | - |
| *GPSM1* | 1.29E-06; up | - | 7.77E-11; down |
| *VSIG10* | 6.30E-08; down | - | - |
| *SARDH* | 5.09E-10; down | - | - |
| *KRT73* | 1.02E-07; up | - | - |
| *TCP10L2* | - | - | - |
| *KCNMB3* | - | - | - |
| *REP15* | - | - | - |
| *CPPED1* | - | - | - |
| *RFC2* | - | - | - |
| *DEM1* | - | - | - |
| *ELF3* | - | - | - |
| *SYDE2* | - | - | - |
| *ZNF527* | - | - | - |
| *LNP1* | - | - | - |
| *TMPRSS7* | - | - | - |
| *KIAA1407* | - | - | - |
| *AKD1* | - | - | - |
| *E2F5* | - | - | - |
| *SPTLC3* | - | - | - |
| *TAS2R10* | - | - | - |
| *ATP7B* | - | - | - |
| *CAPN1* | - | - | - |
| *CAPN10* | - | - | - |
| *RASSF6* | - | - | - |
| *MLL5* | - | - | - |
| *TCF7L2* | - | - | - |
| *ZNF3* | - | - | - |
| *IFT74* | - | - | - |
| *GTF3C5* | - | - | - |
| *EMR1* | - | - | - |
| *FBXW10* | - | - | - |
| *DYRK3* | - | - | - |
| *C2orf81* | - | - | - |
| *EZH2* | - | - | - |
| *SLFN12L* | - | - | - |
| *ATP8B1* | - | - | - |
| *TMEM91* | - | - | - |
| *SPHK2* | - | - | - |
| *C9orf9* | - | - | - |

**Table S10: Kaplan-Meier survival analysis of 71 genes in CRC (Data from HPA database using best seperation)**

| **Gene** | **Combined** | | **Colon (438 cases)** | | **Rectum(159 cases)** | |
| --- | --- | --- | --- | --- | --- | --- |
|  | **Five year survival (High vs low)** | **Log-rank *P* value** | **Five year survival (High vs low)** | **Log-rank *P* value** | **Five year survival (High vs low)** | **Log-rank *P* value** |
| *KCNMB3* | 31% vs 71% | 6.76E-05 | 25% vs 73% | 9.81E-05 | 52% vs 0% | 3.01E-01 |
| *REP15* | 74% vs 52% | 9.43E-05 | 71% vs 56% | 3.88E-04 | 90% vs 39% | 4.94E-02 |
| *SSPO* | 34% vs 69% | 1.95E-04 | 31% vs 73% | 8.53E-07 | 50% vs 53% | 3.43E-02 |
| *ATP8B1* | 77% vs 49% | 2.60E-04 | 74% vs 52% | 1.58E-03 | 97% vs 36% | 5.61E-03 |
| *GPSM1* | 49% vs 64% | 6.07E-04 | 47% vs 67% | 7.18E-05 | 44% vs 53% | 7.96E-03 |
| *BMP5* | 67% vs 46% | 9.07E-04 | 71% vs 45% | 4.80E-03 | 82% vs 42% | 3.32E-02 |
| *CPPED1* | 67% vs 53% | 2.04E-03 | 68% vs 56% | 1.72E-02 | 63% vs 39% | 1.31E-02 |
| *TMEM91* | 54% vs 70% | 5.13E-03 | 57% vs 70% | 7.84E-03 | 44% vs 92% | 7.61E-02 |
| *C3orf20* | 64% vs 53% | 5.88E-03 | 64% vs 56% | 7.42E-02 | 92% vs 26% | 2.32E-03 |
| *C9orf9* | 55% vs 73% | 8.00E-03 | 56% vs 77% | 2.98E-03 | 47% vs 0% | 1.14E-01 |
| *LNP1* | 53% vs 67% | 8.33E-03 | 58% vs 66% | 2.23E-02 | 0% vs 65% | 1.01E-01 |
| *CAPN10* | 45% vs 65% | 9.29E-03 | 53% vs 66% | 1.63E-02 | 36% vs 60% | 9.00E-02 |
| *ZNF404* | 50% vs 65% | 1.11E-02 | 61% vs 71% | 6.84E-02 | 38% vs 81% | 6.45E-03 |
| *PRR11* | 72% vs 55% | 1.30E-02 | 73% vs 58% | 1.97E-02 | 56% vs 29% | 1.21E-01 |
| *IFT74* | 65% vs 57% | 1.33E-02 | 67% vs 60% | 1.46E-01 | 61% vs 39% | 6.13E-03 |
| *SVEP1* | 43% vs 65% | 1.62E-02 | 41% vs 68% | 8.38E-03 | 58% vs 29% | 3.34E-01 |
| *AKD1* | 66% vs 54% | 1.66E-02 | 67% vs 59% | 1.15E-01 | 72% vs 40% | 2.71E-02 |
| *ZNF527* | 67% vs 53% | 1.68E-02 | 74% vs 55% | 8.98E-02 | 66% vs 20% | 3.99E-02 |
| *PHLDB2* | 65% vs 59% | 2.15E-02 | 67% vs 61% | 3.57E-02 | 50% vs 53% | 1.38E-01 |
| *SARDH* | 52% vs 62% | 2.31E-02 | 55% vs 64% | 7.12E-03 | 43% vs 60% | 2.21E-01 |
| *SYDE2* | 64% vs 51% | 2.34E-02 | 66% vs 56% | 3.27E-02 | 61% vs 38% | 6.35E-02 |
| *E2F5* | 68% vs 57% | 3.15E-02 | 73% vs 59% | 3.19E-02 | 73% vs 30% | 1.45E-01 |
| *DYRK3* | 56% vs 69% | 3.36E-02 | 57% vs 79% | 7.95E-02 | 52% vs 53% | 1.28E-01 |
| *DEM1* | 69% vs 55% | 3.69E-02 | 68% vs 58% | 9.65E-02 | 77% vs 30% | 5.81E-02 |
| *C2orf81* | 54% vs 73% | 3.71E-02 | 59% vs 71% | 1.30E-01 | 30% vs 83% | 6.64E-02 |
| *APC* | 64% vs 55% | 3.72E-02 | 61% vs 63% | 7.80E-02 | 74% vs 0% | 7.37E-02 |
| *DUOX2* | 76% vs 58% | 3.77E-02 | 80% vs 60% | 9.72E-02 | 64% vs 27% | 2.83E-01 |
| *SLFN12L* | 66% vs 51% | 3.77E-02 | 65% vs 58% | 1.22E-01 | 70% vs 19% | 7.96E-02 |
| *CTSA* | 55% vs 82% | 4.30E-02 | 63% vs 63% | 1.38E-01 | 40% vs 80% | 8.28E-02 |
| *SPTLC3* | 68% vs 46% | 4.46E-02 | 65% vs 60% | 1.08E-01 | 64% vs 37% | 1.64E-02 |
| *PDE2A* | 55% vs 61% | 4.58E-02 | 55% vs 64% | 3.66E-02 | 51% vs 50% | 1.20E-01 |
| *RASSF6* | 68% vs 46% | 4.82E-02 | 70% vs 48% | 9.87E-03 | 45% vs 52% | 2.14E-01 |
| *LDB3* | 58% vs 64% | 4.87E-02 | 56% vs 70% | 6.79E-02 | 46% vs 53% | 1.15E-01 |
| *TCP10L2* | 62% vs 59% | 5.07E-02 | 65% vs 59% | 1.11E-01 | 35% vs 67% | 1.66E-01 |
| *MGAT5* | 62% vs 54% | 5.39E-02 | 64% vs 55% | 2.75E-01 | 59% vs 0% | 3.24E-03 |
| *GTF3C5* | 51% vs 66% | 5.58E-02 | 55% vs 66% | 8.36E-02 | 42% vs 92% | 1.16E-01 |
| *TMEM178* | 52% vs 67% | 5.94E-02 | 54% vs 69% | 1.87E-02 | 56% vs 57% | 7.88E-02 |
| *DNAH5* | 73% vs 58% | 5.94E-02 | 71% vs 61% | 1.08E-01 | 54% vs 0% | 5.31E-02 |
| *MLL5* | 47% vs 67% | 6.49E-02 | 52% vs 72% | 1.11E-01 | 56% vs 0% | 1.57E-01 |
| *ELF3* | 54% vs 67% | 7.50E-02 | 55% vs 72% | 1.23E-01 | 46% vs 45% | 1.10E-01 |
| *TMPRSS7* | 41% vs 68% | 9.05E-02 | 42% vs 68% | 1.31E-01 | 52% vs 46% | 2.19E-01 |
| *COPB2* | 66% vs 54% | 9.38E-02 | 64% vs 60% | 2.25E-01 | 75% vs 28% | 1.77E-02 |
| *FBXW10* | 55% vs 62% | 9.59E-02 | 59% vs 64% | 9.37E-02 | 59% vs 64% | 2.00E+00 |
| *SPHK2* | 63% vs 60% | 9.61E-02 | 57% vs 64% | 2.30E-02 | 77% vs 46% | 2.76E-01 |
| *ZNF3* | 51% vs 64% | 1.00E-01 | 50% vs 67% | 6.05E-02 | 58% vs 40% | 3.25E-01 |
| *TCF7L2* | 72% vs 55% | 1.11E-01 | 74% vs 57% | 2.53E-02 | 38% vs 57% | 6.40E-02 |
| *HFM1* | 59% vs 61% | 1.17E-01 | 58% vs 64 % | 1.64E-01 | 50% vs 51% | 1.62E-01 |
| *SLCO1B3* | 64% vs 52% | 1.25E-01 | 47% vs 65% | 1.06E-01 | 94% vs 32% | 1.32E-02 |
| *DDI2* | 70% vs 57% | 1.26E-01 | 66% vs 61% | 1.60E-01 | 58% vs 0% | 3.13E-02 |
| *EMR1* | 67% vs 51% | 1.32E-01 | 67% vs 63% | 2.72E-01 | 84% vs 35% | 1.22E-02 |
| *RFC2* | 56% vs 64% | 1.44E-01 | 65% vs 48% | 7.73E-02 | 21% vs 65% | 3.26E-02 |
| *RIMS1* | 54% vs 62% | 1.48E-01 | 55% vs 64% | 5.48E-02 | 63% vs 38% | 1.10E-01 |
| *POLR1A* | 65% vs 54% | 1.55E-01 | 51% vs 66% | 3.71E-01 | 67% vs 0% | 1.62E-02 |
| *EZH2* | 61% vs 55% | 1.57E-01 | 64% vs 56% | 1.73E-01 | 59% vs 0% | 1.59E-01 |
| *EYS* | 59% vs 63% | 1.64E-01 | 64% vs 64% | 2.98E-01 | 62% vs 28% | 4.07E-03 |
| *LIMCH1* | 60% vs 63% | 1.67E-01 | 62% vs 65% | 2.29E-01 | 44% vs 51% | 1.32E-01 |
| *TAS2R10* | 63% vs 58% | 1.77E-01 | 57% vs 75% | 1.16E-01 | 55% vs 33% | 1.02E-01 |
| *ATP6V0D2* | 61% vs 57% | 1.78E-01 | 63% vs 63% | 2.58E-01 | 58% vs 30% | 1.10E-01 |
| *KIAA1407* | 49% vs 66% | 1.86E-01 | 43% vs 71 | 1.28E-02 | 64% vs 32% | 2.07E-02 |
| *VSIG10* | 59% vs 64% | 2.05E-01 | 59% vs 70% | 1.16E-01 | 58% vs 30% | 3.08E-01 |
| *ATP7B* | 64% vs 58% | 2.15E-01 | 59% vs 64% | 1.78E-01 | 94% vs 43% | 4.56E-02 |
| *ZNF175* | 55% vs 63% | 2.44E-01 | 51% vs 67% | 1.82E-01 | 62% vs 21% | 1.10E-01 |
| *SLC16A4* | 66% vs 55% | 2.56E-01 | 69% vs 54% | 3.10E-01 | 51% vs 49% | 2.12E-01 |
| *CAPN1* | 64% vs 48% | 2.69E-01 | 65% vs 52% | 2.57E-01 | 55% vs 37% | 3.09E-01 |
| *DHX30* | 62% vs 58% | 3.27E-01 | 59% vs 63% | 1.55E-01 | 71% vs 37% | 8.60E-03 |
| *AHNAK* | 63% vs 60% | 3.46E-01 | 60% vs 63% | 8.14E-02 | 64% vs 0% | 5.13E-02 |
| *MEPE* | NA | NA | NA | NA | NA | NA |
| *METTL21C* | NA | NA | NA | NA | NA | NA |
| *CCDC54* | NA | NA | NA | NA | NA | NA |
| *AMBN* | NA | NA | NA | NA | NA | NA |
| *KRT73* | NA | NA | NA | NA | NA | NA |

**Table S11: Gene function and pathway annotation**

| **Gene** | **Gene Ontology** | **KEGG Pathways** |
| --- | --- | --- |
| *APC* | beta-catenin binding | Wnt signaling pathway |
| *TCF7L2* | transcription factor activity, sequence-specific DNA binding | Wnt signaling pathway |
| *BMP5* | growth factor activity, cytokine activity | TGF-beta signaling pathway |
| *RASSF6* | protein binding | Hippo signaling pathway |
| *KCNMB3* | calcium-activated potassium channel activity | cGMP-PKG signaling pathway |
| *RFC2* | protein binding | Mismatch repair |
| *MLL5* | protein binding | Lysine degradation |
| *SARDH* | oxidoreductase activity | Glycine, serine and threonine metabolism |
| *RIMS1* | RNA binding | Synaptic vesicle cycle |
| *EZH2* | chromatin binding | Lysine degradation |
| *DUOX2* | calcium ion binding | Thyroid hormone synthesis |
| *ELF3* | transcription factor activity, sequence-specific DNA binding | Circadian rhythm - plant |
| *ATP7B* | copper ion binding | Platinum drug resistance |
| *PDE2A* | cyclic-nucleotide phosphodiesterase activity | Purine metabolism |
| *CAPN1* | calcium-dependent cysteine-type endopeptidase activity | Apoptosis |
| *MGAT5* | transferase activity | N-Glycan biosynthesis |
| *CTSA* | serine-type carboxypeptidase activity | Renin-angiotensin system |
| *DDI2* | ubiquitin binding | Microbial metabolism in diverse environments |
| *GPSM1* | GDP-dissociation inhibitor activity | Cocaine addiction |
| *DYRK3* | protein kinase activity | Null |
| *EYS* | calcium ion binding | Null |
| *HFM1* | nucleic acid binding | Null |
| *FBXW10* | ubiquitin-protein transferase activity | Null |
| *ATP6V0D2* | protein binding | Null |
| *LIMCH1* | metal ion binding | Null |
| *KRT73* | protein binding | Null |
| *LDB3* | cytoskeletal protein binding | Null |
| *EMR1* | integral component of membrane | Null |
| *TMPRSS7* | serine-type endopeptidase activity | Null |
| *COPB2* | protein binding | Null |
| *SPHK2* | kinase activity | Null |
| *GTF3C5* | protein binding | Null |
| *SLCO1B3* | organic anion transmembrane transporter activity | Null |
| *SYDE2* | GTPase activator activity | Null |
| *SPTLC3* | transferase activity | Null |
| *AKD1* | nucleoside diphosphate kinase activity | Null |
| *MEPE* | extracellular matrix protein binding | Null |
| *CPPED1* | phosphoprotein phosphatase activity | Null |
| *CAPN10* | calcium-dependent cysteine-type endopeptidase activity | Null |
| *DHX30* | chromatin binding | Null |
| *ATP8B1* | phospholipid-translocating ATPase activity | Null |
| *PHLDB2* | cadherin binding | Null |
| *POLR1A* | chromatin binding | Null |
| *ZNF527* | DNA binding | Null |
| *IFT74* | chromatin binding | Null |
| *ZNF3* | zinc ion binding | Null |
| *TAS2R10* | G-protein coupled receptor activity | Null |
| *AMBN* | protein binding | Null |
| *METTL21C* | protein-lysine N-methyltransferase activity | Null |
| *SLC16A4* | symporter activity | Null |
| *ZNF175* | RNA polymerase II regulatory region sequence-specific DNA binding | Null |
| *C9orf9* | calcium-dependent protein binding | Null |
| *SSPO* | peptidase inhibitor activity | Null |
| *SLFN12L* | ATP binding | Null |
| *ZNF404* | protein binding | Null |
| *E2F5* | protein binding | Null |
| *DEM1* | single-stranded DNA 3-5 exodeoxyribonuclease activity | Null |
| *REP15* | protein binding | Null |
| *DNAH5* | ATP binding | Null |
| *AHNAK* | cadherin binding | Null |
| *SVEP1* | chromatin binding | Null |
| *KIAA1407* | Null | Null |
| *C3orf20* | Null | Null |
| *TMEM178* | Null | Null |
| *CCDC54* | Null | Null |
| *VSIG10* | Null | Null |
| *PRR11* | Null | Null |
| *LNP1* | Null | Null |
| *TMEM91* | Null | Null |
| *TCP10L2* | Null | Null |
| *C2orf81* | Null | Null |

**Table S12: Correlation of BMP5 expression to age, gender and clinical grade of 129 colorectal adenocarcinomas**

| **Variables** | **Cases** | **BMP5 staining** | | | | **χ2** | ***P* value** |
| --- | --- | --- | --- | --- | --- | --- | --- |
|  |  | **0** | **1+** | **2+** | **3+** |  |  |
| **Age(y)** |  |  |  |  |  |  |  |
| **≥55** | 79 | 24 | 30 | 18 | 7 | 0.1041 | 0.9913 |
| **<55** | 50 | 16 | 18 | 12 | 4 |  |  |
|  |  |  |  |  |  |  |  |
| **Gender** |  |  |  |  |  |  |  |
| **Male** | 78 | 29 | 30 | 15 | 4 | 6.554 | 0.0876 |
| **Female** | 51 | 11 | 18 | 15 | 7 |  |  |
|  |  |  |  |  |  |  |  |
| **Grade** |  |  |  |  |  |  |  |
| **I** | 19 | 6 | 5 | 8 | 0 | 8.504 | 0.2035 |
| **II**  **III** | 87  23 | 24  10 | 34  7 | 19  3 | 8  3 |  |  |

**Table S13: Correlation of BMP5 expression to age, gender, BMI, and recurrence of TCGA CRC samples**

| **Variables** | **Cases** | **BMP5 expression** | | **χ2** | ***P* Value** |
| --- | --- | --- | --- | --- | --- |
|  |  | **High** | **Low** |  |  |
| **Age(years）** |  |  |  |  |  |
| >65 | 192 | 102 | 90 | 0.822 | 0.365 |
| ≤65 | 187 | 108 | 79 |  |  |
|  |  |  |  |  |  |
| **Gender** |  |  |  |  |  |
| Male | 208 | 112 | 96 | 0.456 | 0.500 |
| Female | 171 | 98 | 73 |  |  |
|  |  |  |  |  |  |
| **BMI** |  |  |  |  |  |
| >25 | 193 | 112 | 81 | 4.199 | **0.040** |
| ≤25 | 89 | 40 | 49 |  |  |
|  |  |  |  |  |  |
| **Recurrence** |  |  |  |  |  |
| Yes | 84 | 37 | 47 | 5.093 | **0.024** |
| No | 265 | 154 | 111 |  |  |

**Table S14: Differentially expressed genes (Control vs BMP5 transfected in HT-29 cells)**

| **AccID** | **BMP5** | **Control** | **FoldChange** | **Log2FC** | **P-Value** | **FDR** | **Style** | **KeggID** |
| --- | --- | --- | --- | --- | --- | --- | --- | --- |
| BMP5 | 2747.233 | 0 | Inf | 20 | 0 | 0 | up | hsa:653 |
| HLA-B | 7219.405 | 20219.65 | 0.357049 | -1.48581 | 1.76E-195 | 1.70E-191 | down | hsa:3106 |
| IFITM1 | 3596.239 | 11677.29 | 0.307969 | -1.69914 | 2.90E-182 | 1.87E-178 | down | hsa:8519 |
| DHRS2 | 433.3582 | 1628.041 | 0.266184 | -1.90951 | 4.72E-121 | 2.28E-117 | down | hsa:10202 |
| UBE2L6 | 1492.567 | 3784.151 | 0.394426 | -1.34217 | 2.16E-117 | 8.34E-114 | down | hsa:9246 |
| IFITM3 | 7769.862 | 16396.76 | 0.473866 | -1.07745 | 3.78E-104 | 1.22E-100 | down | hsa:10410 |
| PSMB9 | 459.2855 | 1388.46 | 0.330788 | -1.59602 | 3.27E-99 | 9.02E-96 | down | hsa:5698 |
| HLA-C | 10236.41 | 20376.22 | 0.502371 | -0.99318 | 2.29E-91 | 5.53E-88 | down | hsa:3107 |
| TRIM22 | 326.8747 | 1162.142 | 0.281269 | -1.82998 | 1.29E-86 | 2.77E-83 | down | hsa:10346 |
| CMPK2 | 1024.406 | 2346.021 | 0.436657 | -1.19543 | 3.45E-79 | 6.67E-76 | down | hsa:129607 |
| MX2 | 1666.065 | 3471.814 | 0.479883 | -1.05924 | 1.75E-74 | 3.07E-71 | down | hsa:4600 |
| HLA-F | 869.6174 | 2206.729 | 0.394075 | -1.34346 | 9.05E-72 | 1.46E-68 | down | hsa:3134 |
| CFB | 745.4677 | 1666.498 | 0.447326 | -1.1606 | 1.10E-64 | 1.64E-61 | down | hsa:629 |
| MX1 | 4239.851 | 7767.345 | 0.545856 | -0.87341 | 4.04E-64 | 5.58E-61 | down | hsa:4599 |
| IFIT3 | 2123.076 | 4081.127 | 0.520218 | -0.94281 | 4.56E-64 | 5.87E-61 | down | hsa:3437 |
| SLC15A3 | 272.8708 | 781.3264 | 0.34924 | -1.51771 | 6.48E-64 | 7.82E-61 | down | hsa:51296 |
| OAS2 | 9446.717 | 16647.02 | 0.567472 | -0.81738 | 3.70E-62 | 4.21E-59 | down | hsa:4939 |
| HLA-E | 4765.403 | 8451.32 | 0.563865 | -0.82658 | 2.51E-58 | 2.69E-55 | down | hsa:3133 |
| HCP5 | 258.9778 | 736.0053 | 0.35187 | -1.50689 | 5.47E-57 | 5.57E-54 | down | hsa:10866 |
| TAP1 | 1881.493 | 4037.224 | 0.466036 | -1.10149 | 1.05E-53 | 1.01E-50 | down | hsa:6890 |
| RSAD2 | 406.1899 | 971.6483 | 0.418042 | -1.25828 | 1.74E-50 | 1.60E-47 | down | hsa:91543 |
| OAS1 | 5325.923 | 9028.613 | 0.589894 | -0.76147 | 5.24E-47 | 4.60E-44 | down | hsa:4938 |
| IFI35 | 1671.901 | 2968.037 | 0.563302 | -0.82802 | 1.62E-45 | 1.36E-42 | down | hsa:3430 |
| NLRC5 | 407.0432 | 899.2298 | 0.452658 | -1.14351 | 6.50E-44 | 5.23E-41 | down | hsa:84166 |
| OASL | 3624.051 | 6003.105 | 0.603696 | -0.72811 | 1.10E-43 | 8.49E-41 | down | hsa:8638 |
| IFI44L | 1541.423 | 2707.565 | 0.569302 | -0.81273 | 4.63E-43 | 3.44E-40 | down | hsa:10964 |
| CXCL10 | 93.05179 | 316.0698 | 0.294403 | -1.76414 | 6.52E-40 | 4.67E-37 | down | hsa:3627 |
| IFIT1 | 2478.278 | 4660.678 | 0.531742 | -0.9112 | 8.32E-40 | 5.74E-37 | down | hsa:3434 |
| IFI6 | 13163.63 | 20456.2 | 0.643503 | -0.63598 | 2.14E-39 | 1.43E-36 | down | hsa:2537 |
| PSMB8 | 1055.047 | 1882.214 | 0.560535 | -0.83512 | 5.58E-38 | 3.59E-35 | down | hsa:5696 |
| ISG20 | 1151.581 | 1987.055 | 0.579542 | -0.78702 | 2.23E-35 | 1.39E-32 | down | hsa:3669 |
| GBP1 | 158.6078 | 411.7235 | 0.385229 | -1.37621 | 5.46E-34 | 3.30E-31 | down | hsa:2633 |
| SLFN5 | 519.3543 | 991.8401 | 0.523627 | -0.93339 | 2.47E-33 | 1.45E-30 | down | hsa:162394 |
| IFITM2 | 1389.84 | 2728.105 | 0.509452 | -0.97298 | 1.27E-32 | 7.19E-30 | down | hsa:10581 |
| TAP2 | 3153.47 | 4898.987 | 0.643698 | -0.63554 | 4.08E-32 | 2.19E-29 | down | hsa:6891 |
| IFI16 | 89.49429 | 278.4637 | 0.321386 | -1.63762 | 6.47E-32 | 3.38E-29 | down | hsa:3428 |
| GSDMB | 317.722 | 663.8347 | 0.478616 | -1.06306 | 1.08E-31 | 5.49E-29 | down | hsa:55876 |
| XAF1 | 540.0668 | 1005.713 | 0.536999 | -0.89701 | 1.97E-31 | 9.75E-29 | down | hsa:54739 |
| DSPA2D | 246.3876 | 540.8933 | 0.45552 | -1.13441 | 9.06E-31 | 4.38E-28 | down |  |
| LOC100419583 | 247.0075 | 544.4177 | 0.45371 | -1.14016 | 1.11E-30 | 5.21E-28 | down |  |
| RPLP2P5 | 569.4667 | 1082.045 | 0.526288 | -0.92608 | 2.00E-30 | 9.19E-28 | down |  |
| HLA-H | 1484.474 | 2449.63 | 0.605999 | -0.72261 | 2.67E-30 | 1.20E-27 | down | hsa:3136 |
| IFI30 | 854.977 | 1460.759 | 0.585297 | -0.77276 | 1.35E-29 | 5.93E-27 | down | hsa:10437 |
| ETV7 | 153.9919 | 418.9702 | 0.367549 | -1.44399 | 3.59E-29 | 1.54E-26 | down | hsa:51513 |
| EPSTI1 | 413.5921 | 782.4834 | 0.528563 | -0.91985 | 2.33E-28 | 9.78E-26 | down | hsa:94240 |
| PARP12 | 2203.998 | 3390.272 | 0.650095 | -0.62128 | 5.96E-28 | 2.45E-25 | down | hsa:64761 |
| PML | 739.0824 | 1261.461 | 0.585894 | -0.77129 | 1.44E-27 | 5.80E-25 | down | hsa:5371 |
| CCL5 | 52.83485 | 189.2449 | 0.279188 | -1.84069 | 4.39E-27 | 1.73E-24 | down | hsa:6352 |
| TRIM21 | 668.0855 | 1150.94 | 0.58047 | -0.78471 | 4.56E-27 | 1.76E-24 | down | hsa:6737 |
| RTP4 | 140.0042 | 396.2213 | 0.353349 | -1.50084 | 5.63E-27 | 2.13E-24 | down | hsa:64108 |
| HLA-G | 386.3949 | 810.0222 | 0.477018 | -1.06789 | 1.32E-26 | 4.89E-24 | down | hsa:3135 |
| TNFSF10 | 748.7019 | 1272.491 | 0.588375 | -0.76519 | 5.56E-25 | 2.03E-22 | down | hsa:8743 |
| IFIH1 | 1327.031 | 2249.062 | 0.590037 | -0.76112 | 1.57E-24 | 5.40E-22 | down | hsa:64135 |
| UBA7 | 374.4005 | 695.3929 | 0.538401 | -0.89325 | 1.69E-24 | 5.73E-22 | down | hsa:7318 |
| IGHV3OR16-6 | 268.3182 | 103.0459 | 2.60387 | 1.380658 | 2.37E-24 | 7.90E-22 | up |  |
| STAT2 | 345.277 | 656.6305 | 0.525832 | -0.92733 | 5.87E-24 | 1.89E-21 | down | hsa:6773 |
| SAMD9L | 141.3656 | 328.701 | 0.430073 | -1.21734 | 1.33E-23 | 4.20E-21 | down | hsa:219285 |
| B2M | 23237.68 | 36872.4 | 0.630219 | -0.66608 | 7.50E-23 | 2.30E-20 | down | hsa:567 |
| HSH2D | 338.7579 | 624.2244 | 0.542686 | -0.88181 | 2.51E-22 | 7.59E-20 | down | hsa:84941 |
| BTN3A3 | 172.074 | 373.4124 | 0.460815 | -1.11774 | 2.81E-22 | 8.35E-20 | down | hsa:10384 |
| LAMP3 | 91.76294 | 241.2573 | 0.380353 | -1.39459 | 3.24E-22 | 9.48E-20 | down | hsa:27074 |
| MDK | 340.2888 | 622.4802 | 0.546666 | -0.87127 | 5.47E-22 | 1.55E-19 | down | hsa:4192 |
| BTN3A2 | 414.5478 | 715.6446 | 0.579265 | -0.78771 | 1.99E-20 | 5.56E-18 | down | hsa:11118 |
| CD68 | 490.8661 | 821.072 | 0.597836 | -0.74218 | 6.20E-20 | 1.71E-17 | down | hsa:968 |
| SAMHD1 | 1068.461 | 1606.922 | 0.664912 | -0.58877 | 3.00E-19 | 8.17E-17 | down | hsa:25939 |
| RPL10P3 | 109.5071 | 255.3991 | 0.428768 | -1.22173 | 3.69E-19 | 9.91E-17 | down |  |
| MT2A | 384.4974 | 658.2866 | 0.584088 | -0.77574 | 1.38E-18 | 3.60E-16 | down | hsa:4502 |
| IFIT2 | 928.1747 | 1641.413 | 0.565473 | -0.82247 | 1.60E-17 | 4.01E-15 | down | hsa:3433 |
| TRANK1 | 382.943 | 668.211 | 0.573087 | -0.80317 | 4.22E-16 | 1.02E-13 | down | hsa:9881 |
| BTN3A1 | 230.9057 | 414.8544 | 0.556595 | -0.8453 | 1.36E-15 | 3.12E-13 | down | hsa:11119 |
| BATF2 | 73.32677 | 179.3214 | 0.408913 | -1.29014 | 2.17E-15 | 4.93E-13 | down | hsa:116071 |
| DHX58 | 554.1775 | 852.7575 | 0.649865 | -0.62179 | 4.33E-15 | 9.74E-13 | down | hsa:79132 |
| ZBP1 | 75.53436 | 230.6776 | 0.327446 | -1.61067 | 6.48E-15 | 1.44E-12 | down | hsa:81030 |
| CXCL11 | 143.167 | 278.0297 | 0.514934 | -0.95754 | 1.58E-14 | 3.42E-12 | down | hsa:6373 |
| SLC25A28 | 484.9503 | 749.0188 | 0.647447 | -0.62716 | 1.99E-14 | 4.27E-12 | down | hsa:81894 |
| USP18 | 533.2945 | 897.0281 | 0.594513 | -0.75022 | 4.87E-14 | 9.80E-12 | down | hsa:11274 |
| GBP4 | 92.57388 | 194.828 | 0.475157 | -1.07352 | 1.18E-12 | 2.22E-10 | down | hsa:115361 |
| LOC728048 | 91.91928 | 192.3764 | 0.47781 | -1.06549 | 2.51E-12 | 4.58E-10 | down |  |
| ARHGAP23P1 | 80.46429 | 217.9337 | 0.369215 | -1.43747 | 2.78E-12 | 5.02E-10 | down |  |
| CSF3 | 647.684 | 1347.316 | 0.480722 | -1.05673 | 2.95E-12 | 5.29E-10 | down | hsa:1440 |
| IL32 | 64.62939 | 150.9084 | 0.428269 | -1.22341 | 3.46E-12 | 6.13E-10 | down | hsa:9235 |
| TRIM5 | 410.5267 | 622.4706 | 0.659512 | -0.60053 | 4.85E-12 | 8.44E-10 | down | hsa:85363 |
| RPLP2P2 | 179.6483 | 314.3015 | 0.57158 | -0.80697 | 6.67E-12 | 1.14E-09 | down |  |
| ACO1 | 332.2959 | 192.6197 | 1.72514 | 0.786713 | 7.55E-12 | 1.28E-09 | up | hsa:48 |
| CTSS | 190.8421 | 335.8477 | 0.56824 | -0.81543 | 1.35E-11 | 2.27E-09 | down | hsa:1520 |
| HLA-J | 93.10955 | 188.6612 | 0.493528 | -1.0188 | 2.49E-11 | 4.16E-09 | down | hsa:3137 |
| USP41 | 69.93959 | 150.2425 | 0.465511 | -1.10311 | 6.22E-11 | 1.01E-08 | down | hsa:373856 |
| TMEM140 | 188.8471 | 318.6782 | 0.592595 | -0.75488 | 1.10E-10 | 1.76E-08 | down | hsa:55281 |
| LOC442309 | 55.22469 | 126.4212 | 0.436831 | -1.19485 | 3.61E-10 | 5.63E-08 | down |  |
| APOL3 | 118.2914 | 216.2126 | 0.547107 | -0.87011 | 4.82E-10 | 7.28E-08 | down | hsa:80833 |
| CX3CL1 | 142.486 | 247.4823 | 0.575742 | -0.79651 | 9.17E-10 | 1.34E-07 | down | hsa:6376 |
| TYMP | 122.1814 | 219.2038 | 0.557387 | -0.84325 | 1.27E-09 | 1.84E-07 | down | hsa:1890 |
| CCL20 | 88.4854 | 170.2276 | 0.519807 | -0.94395 | 1.99E-09 | 2.81E-07 | down | hsa:6364 |
| UBD | 7.138896 | 38.65564 | 0.184679 | -2.43691 | 2.03E-09 | 2.85E-07 | down | hsa:10537 |
| AKR1C1 | 406.8092 | 267.849 | 1.5188 | 0.602932 | 4.03E-09 | 5.54E-07 | up | hsa:1645 |
| ISG15 | 12510.34 | 18884.12 | 0.662479 | -0.59405 | 5.29E-09 | 7.09E-07 | down | hsa:9636 |
| RPL21P34 | 5.552178 | 35.94645 | 0.154457 | -2.69472 | 6.32E-09 | 8.38E-07 | down |  |
| HLA-L | 111.103 | 195.9203 | 0.567083 | -0.81837 | 1.38E-08 | 1.78E-06 | down | hsa:3139 |
| PLEKHA4 | 224.6503 | 345.6515 | 0.649933 | -0.62164 | 1.60E-08 | 2.01E-06 | down | hsa:57664 |
| RPL21P86 | 2.403229 | 28.11418 | 0.085481 | -3.54825 | 2.98E-08 | 3.51E-06 | down |  |
| IL15RA | 226.2274 | 343.4113 | 0.658765 | -0.60216 | 5.54E-08 | 6.37E-06 | down | hsa:3601 |
| TNFAIP3 | 158.331 | 255.4344 | 0.61985 | -0.69001 | 7.65E-08 | 8.69E-06 | down | hsa:7128 |
| IFITM9P | 62.6749 | 122.153 | 0.513085 | -0.96273 | 1.70E-07 | 1.80E-05 | down |  |
| CD74 | 130.7511 | 211.4229 | 0.618434 | -0.69331 | 4.54E-07 | 4.57E-05 | down | hsa:972 |
| PLA1A | 3.087883 | 22.19919 | 0.139099 | -2.84582 | 6.32E-07 | 6.26E-05 | down | hsa:51365 |
| RPL32P27 | 108.7599 | 229.0866 | 0.474755 | -1.07475 | 1.40E-06 | 1.31E-04 | down |  |
| LGALS9DP | 44.2679 | 90.35354 | 0.489941 | -1.02932 | 1.93E-06 | 1.73E-04 | down |  |
| CSF1 | 89.19454 | 152.04 | 0.586652 | -0.76942 | 2.53E-06 | 2.20E-04 | down | hsa:1435 |
| NUPR1 | 17.39253 | 54.70649 | 0.317924 | -1.65324 | 2.88E-06 | 2.45E-04 | down | hsa:26471 |
| RASGRP3 | 12.9654 | 40.13066 | 0.32308 | -1.63004 | 3.90E-06 | 3.22E-04 | down | hsa:25780 |
| MUC17 | 258.9273 | 420.4832 | 0.615785 | -0.6995 | 4.33E-06 | 3.57E-04 | down | hsa:140453 |
| RPL7AP50 | 265.8472 | 443.5057 | 0.599422 | -0.73836 | 4.87E-06 | 3.95E-04 | down |  |
| LOC100101246 | 23.69132 | 56.31001 | 0.42073 | -1.24903 | 7.42E-06 | 5.64E-04 | down |  |
| C5orf56 | 39.72841 | 79.09747 | 0.502272 | -0.99346 | 1.14E-05 | 8.22E-04 | down | hsa:441108 |
| LAP3P2 | 150.3003 | 228.8112 | 0.656875 | -0.60631 | 1.17E-05 | 8.42E-04 | down |  |
| KRT13 | 196.0822 | 130.4348 | 1.503297 | 0.58813 | 2.43E-05 | 0.001596835 | up | hsa:3860 |
| SECTM1 | 80.3778 | 132.4276 | 0.606956 | -0.72034 | 2.72E-05 | 0.001758273 | down | hsa:6398 |
| CTGF | 115.2734 | 177.5465 | 0.649257 | -0.62314 | 2.87E-05 | 0.001841947 | down | hsa:1490 |
| IFNL1 | 11.31945 | 34.06307 | 0.332308 | -1.58941 | 3.04E-05 | 0.001933497 | down | hsa:282618 |
| NAPSB | 14.32498 | 38.77298 | 0.369458 | -1.43652 | 3.78E-05 | 0.002320179 | down | hsa:256236 |
| FRRS1 | 172.2777 | 112.148 | 1.536163 | 0.619331 | 4.06E-05 | 0.002452695 | up | hsa:391059 |
| TRNF | 115.9012 | 195.1641 | 0.593865 | -0.75179 | 4.25E-05 | 0.002543046 | down | hsa:4558 |
| IL2RG | 37.72164 | 81.62015 | 0.462161 | -1.11353 | 4.36E-05 | 0.002575575 | down | hsa:3561 |
| CTSO | 44.87115 | 83.59873 | 0.536744 | -0.89769 | 4.40E-05 | 0.00259169 | down | hsa:1519 |
| PRKACB | 120.0516 | 72.16793 | 1.663504 | 0.734225 | 4.53E-05 | 0.002659648 | up | hsa:5567 |
| GMPR | 2.681702 | 16.71621 | 0.160425 | -2.64003 | 4.74E-05 | 0.002769524 | down | hsa:2766 |
| RPL7P32 | 155.5124 | 100.8797 | 1.541563 | 0.624394 | 5.24E-05 | 0.002994787 | up |  |
| VSIG1 | 33.35326 | 11.35156 | 2.938209 | 1.554937 | 5.72E-05 | 0.00323152 | up | hsa:340547 |
| AHCYP4 | 29.31103 | 60.9007 | 0.481292 | -1.05502 | 6.10E-05 | 0.003387634 | down |  |
| PIGR | 26.16717 | 55.28966 | 0.473274 | -1.07925 | 6.62E-05 | 0.003646785 | down | hsa:5284 |
| CASP1 | 34.439 | 68.05092 | 0.506077 | -0.98257 | 6.92E-05 | 0.00379796 | down | hsa:834 |
| ACKR4 | 21.52263 | 48.46748 | 0.444063 | -1.17116 | 6.99E-05 | 0.00382793 | down | hsa:51554 |
| HLA-W | 13.69725 | 36.81706 | 0.372035 | -1.42649 | 7.27E-05 | 0.003963329 | down |  |
| LOC100101247 | 31.86502 | 76.359 | 0.417305 | -1.26082 | 8.71E-05 | 0.004589716 | down |  |
| TAPBPL | 257.7945 | 387.9405 | 0.664521 | -0.58961 | 1.10E-04 | 0.005588828 | down | hsa:55080 |
| SMG1P1 | 73.83243 | 118.3915 | 0.623629 | -0.68124 | 1.27E-04 | 0.006247622 | down | hsa:641298 |
| PLAC8 | 82.20137 | 129.1197 | 0.636629 | -0.65148 | 1.50E-04 | 0.007203419 | down | hsa:51316 |
| LOC105372935 | 6.061043 | 23.77916 | 0.254889 | -1.97206 | 1.67E-04 | 0.007960059 | down |  |
| TXNP4 | 88.6214 | 137.3434 | 0.645254 | -0.63206 | 1.68E-04 | 0.007980347 | down |  |
| FLT3LG | 36.16913 | 67.56386 | 0.535333 | -0.90149 | 1.76E-04 | 0.00825347 | down | hsa:2323 |
| RPL32P7 | 12.95567 | 33.85319 | 0.382702 | -1.38571 | 1.93E-04 | 0.00889102 | down |  |
| HERC5 | 73.04972 | 116.6074 | 0.626459 | -0.67471 | 1.95E-04 | 0.008927249 | down | hsa:51191 |
| RAET1L | 87.93023 | 135.6235 | 0.648341 | -0.62518 | 2.23E-04 | 0.009999016 | down | hsa:154064 |
| PI3 | 85.97574 | 131.9125 | 0.651763 | -0.61758 | 2.26E-04 | 0.010087497 | down | hsa:5266 |
| LOC105374236 | 23.50397 | 49.49042 | 0.47492 | -1.07424 | 2.58E-04 | 0.011268986 | down |  |
| LOC105371082 | 2.681702 | 14.71665 | 0.182222 | -2.45623 | 2.72E-04 | 0.011641569 | down |  |
| SUSD3 | 27.10255 | 53.63875 | 0.505279 | -0.98485 | 2.84E-04 | 0.011939404 | down | hsa:203328 |
| PSMB8-AS1 | 77.16867 | 145.2283 | 0.531361 | -0.91224 | 3.07E-04 | 0.012636907 | down | hsa:100507463 |
| CDH17 | 83.27276 | 48.98747 | 1.699879 | 0.765432 | 3.33E-04 | 0.01353202 | up | hsa:1015 |
| TNFSF12-TNFSF13 | 87.54444 | 132.4679 | 0.660873 | -0.59756 | 3.75E-04 | 0.014721207 | down | hsa:407977 |
| ZBTB41 | 105.5152 | 65.7865 | 1.603904 | 0.681587 | 3.90E-04 | 0.015162553 | up | hsa:360023 |
| IFNL2 | 7.261027 | 22.84617 | 0.317822 | -1.65371 | 4.31E-04 | 0.016259098 | down | hsa:282616 |
| MR1 | 17.40961 | 39.46681 | 0.44112 | -1.18076 | 5.21E-04 | 0.018873673 | down | hsa:3140 |
| TNFSF13B | 11.65715 | 29.8612 | 0.390378 | -1.35706 | 5.50E-04 | 0.019665531 | down | hsa:10673 |
| MIB2 | 94.26743 | 59.40506 | 1.586858 | 0.666173 | 8.37E-04 | 0.027524949 | up | hsa:142678 |
| SEPT4 | 21.81314 | 44.39063 | 0.491391 | -1.02506 | 8.95E-04 | 0.02900271 | down | hsa:5414 |
| CXCL1 | 54.31331 | 87.76287 | 0.618864 | -0.6923 | 9.45E-04 | 0.030302874 | down | hsa:2919 |
| HEPACAM2 | 64.08814 | 35.20996 | 1.820171 | 0.864074 | 9.71E-04 | 0.030919809 | up | hsa:253012 |
| C17orf67 | 57.1944 | 91.28537 | 0.626545 | -0.67451 | 0.00107821 | 0.033291883 | down | hsa:339210 |
| HNRNPA3P7 | 20.71816 | 42.40552 | 0.488572 | -1.03336 | 0.001114494 | 0.034193728 | down |  |
| ARHGAP33 | 47.00603 | 23.01385 | 2.042511 | 1.030344 | 0.00116655 | 0.035342072 | up | hsa:115703 |
| LOC100420067 | 4.112078 | 15.80438 | 0.260186 | -1.94238 | 0.00121503 | 0.036242761 | down |  |
| TNFRSF1B | 5.376388 | 17.37711 | 0.309395 | -1.69248 | 0.001481236 | 0.042042302 | down | hsa:7133 |
| LOC441880 | 24.01373 | 9.345306 | 2.569603 | 1.361546 | 0.00152627 | 0.043067542 | up |  |
| TXNP5 | 67.78017 | 102.8952 | 0.65873 | -0.60224 | 0.001667493 | 0.046442323 | down |  |
| DUOX1 | 66.89707 | 100.7028 | 0.664302 | -0.59009 | 1.69E-03 | 4.70E-02 | down | hsa:53905 |
| CKMT1B | 88.56782 | 153.3839 | 0.577426 | -0.79229 | 0.00186513 | 0.05042112 | down | hsa:1159 |
| TRIM52-AS1 | 34.03234 | 58.27718 | 0.583974 | -0.77602 | 0.00189159 | 0.051014189 | down | hsa:100507602 |
| PATL2 | 15.22473 | 32.87701 | 0.463081 | -1.11066 | 0.001946269 | 0.052249205 | down | hsa:197135 |
| C4A | 4.559877 | 15.75967 | 0.289338 | -1.78917 | 0.001956156 | 0.052441796 | down | hsa:720 |
| RPL32P21 | 13.09168 | 29.07635 | 0.450252 | -1.1512 | 0.002556431 | 0.063676869 | down |  |
| CAPS | 95.99252 | 63.18252 | 1.519289 | 0.603396 | 0.002586862 | 0.06419577 | up | hsa:828 |
| RASSF4 | 16.81145 | 34.75476 | 0.483716 | -1.04777 | 0.002596799 | 0.06426828 | down | hsa:83937 |
| GPR89B | 92.38836 | 140.5749 | 0.657218 | -0.60556 | 0.002661466 | 0.065700469 | down | hsa:51463 |
| HMGN2P40 | 12.78729 | 27.97113 | 0.45716 | -1.12923 | 0.00282457 | 0.068416187 | down |  |
| NOSTRIN | 88.38455 | 58.01848 | 1.523386 | 0.607282 | 0.003288042 | 0.076030365 | up | hsa:115677 |
| IFNL3 | 6.193788 | 18.04417 | 0.343257 | -1.54264 | 0.003342494 | 0.077096747 | down | hsa:282617 |
| HLA-S | 14.67425 | 31.10375 | 0.471784 | -1.0838 | 0.003462112 | 0.079194286 | down |  |
| CCDC28B | 46.49208 | 73.85685 | 0.629489 | -0.66775 | 0.003637829 | 0.081857497 | down | hsa:79140 |
| C4B | 5.495261 | 16.69051 | 0.329245 | -1.60277 | 0.003657042 | 0.08200345 | down | hsa:721 |
| FOSB | 85.64267 | 56.53007 | 1.514993 | 0.599311 | 0.003706654 | 0.08282764 | up | hsa:2354 |
| RN7SK | 63.19077 | 108.8162 | 0.580711 | -0.78411 | 0.00372119 | 0.082960654 | down | hsa:125050 |
| RPL12P6 | 77.77246 | 116.6886 | 0.666496 | -0.58533 | 0.003905932 | 0.085204866 | down |  |
| GAPDHP62 | 42.55164 | 68.75706 | 0.618869 | -0.69229 | 0.004104152 | 0.088438292 | down |  |
| CA11 | 40.50923 | 65.65335 | 0.617017 | -0.69662 | 0.004275513 | 0.091518696 | down | hsa:770 |
| ST13P17 | 4.593198 | 0 | Inf | 20 | 0.004357846 | 0.092563529 | up |  |
| LOC105374327 | 11.9259 | 26.63702 | 0.447719 | -1.15933 | 0.004415286 | 0.093271118 | down |  |
| C9orf91 | 32.71485 | 55.43101 | 0.59019 | -0.76075 | 0.004459706 | 0.094003991 | down | hsa:203197 |
| VRTN | 38.44465 | 62.2158 | 0.617924 | -0.6945 | 0.004477902 | 0.094284713 | down | hsa:55237 |
| PAX4 | 41.12078 | 21.58916 | 1.904695 | 0.92956 | 0.004918506 | 0.1002846 | up | hsa:5078 |
| BCL2L14 | 20.86157 | 38.42379 | 0.542934 | -0.88115 | 0.004940204 | 0.10051496 | down | hsa:79370 |
| GAPDHP32 | 27.84087 | 48.07198 | 0.57915 | -0.78799 | 0.005043023 | 0.102283928 | down |  |
| RPL36AP19 | 1.359586 | 10.99059 | 0.123705 | -3.01503 | 0.005206355 | 0.104608768 | down |  |
| RPL21P101 | 43.9529 | 74.1799 | 0.592518 | -0.75507 | 0.005376447 | 0.107025072 | down |  |
| BLACAT1 | 44.87856 | 69.63749 | 0.64446 | -0.63384 | 0.005628537 | 0.110901118 | down | hsa:101669762 |
| TMEM229B | 22.786 | 41.4463 | 0.549772 | -0.8631 | 0.005829436 | 0.113235249 | down | hsa:161145 |
| ZSCAN31 | 39.75058 | 62.46674 | 0.636348 | -0.65211 | 0.006177838 | 0.118463729 | down | hsa:64288 |
| HOXA-AS3 | 71.06008 | 46.34602 | 1.533251 | 0.616594 | 0.00660292 | 0.123192891 | up | hsa:100133311 |
| C6orf222 | 32.96558 | 54.42717 | 0.605682 | -0.72337 | 0.006629641 | 0.123200576 | down | hsa:389384 |
| ARHGAP11B | 62.89325 | 95.88257 | 0.65594 | -0.60836 | 0.00668714 | 0.123689692 | down | hsa:89839 |
| LOC123862 | 15.71232 | 31.27241 | 0.502434 | -0.99299 | 0.006913904 | 0.126792084 | down |  |
| RPS26P28 | 76.55855 | 49.67032 | 1.541334 | 0.624179 | 0.006969704 | 0.127694231 | up |  |
| ACTBP12 | 11.33564 | 24.7938 | 0.457196 | -1.12911 | 0.007040929 | 0.128568686 | down |  |
| BEND7 | 6.657776 | 17.9336 | 0.371246 | -1.42955 | 0.007047063 | 0.128568686 | down | hsa:222389 |
| CNTNAP3P2 | 0.311795 | 5.461832 | 0.057086 | -4.13072 | 0.007034867 | 0.128568686 | down |  |
| RPS14P8 | 56.66703 | 86.48452 | 0.655227 | -0.60993 | 0.007077131 | 0.128686613 | down |  |
| TRIM6 | 9.593466 | 22.16627 | 0.432796 | -1.20824 | 0.007163822 | 0.129169325 | down | hsa:117854 |
| CPT1B | 20.43411 | 37.69347 | 0.542113 | -0.88334 | 0.007484082 | 0.133231322 | down | hsa:1375 |
| TMSB10P2 | 7.413221 | 18.94931 | 0.391213 | -1.35397 | 0.007485603 | 0.133231322 | down |  |
| MT2P1 | 29.54231 | 49.8977 | 0.592057 | -0.75619 | 0.007561139 | 0.134081892 | down |  |
| SRP14-AS1 | 32.29895 | 53.13268 | 0.607892 | -0.71811 | 0.007625861 | 0.134366705 | down | hsa:100131089 |
| PTPRN2 | 46.18586 | 27.57054 | 1.675189 | 0.744324 | 0.008329042 | 0.142344881 | up | hsa:5799 |
| COL9A2 | 1.945706 | 9.344771 | 0.208213 | -2.26387 | 0.008547848 | 0.144803983 | down | hsa:1298 |
| FN1 | 7.657485 | 1.274948 | 6.006115 | 2.586432 | 0.008626446 | 0.145342747 | up | hsa:2335 |
| RPS3AP49 | 64.08257 | 41.55481 | 1.542122 | 0.624916 | 0.008790952 | 0.14635685 | up |  |
| ATP1A3 | 7.281365 | 18.54819 | 0.392565 | -1.349 | 0.009204983 | 0.150660282 | down | hsa:478 |
| ZNF385C | 32.2712 | 52.76653 | 0.611585 | -0.70938 | 0.009443266 | 0.153128269 | down | hsa:201181 |
| TMED10P1 | 24.45738 | 43.07445 | 0.567793 | -0.81656 | 0.009649053 | 0.155034539 | down | hsa:286102 |
| C3 | 1.420652 | 7.688774 | 0.18477 | -2.4362 | 0.009726396 | 0.155759323 | down | hsa:718 |
| GDPD3 | 8.566012 | 21.31199 | 0.401934 | -1.31497 | 0.009934158 | 0.157867194 | down | hsa:79153 |
| ARHGAP9 | 0.311795 | 5.158421 | 0.060444 | -4.04826 | 0.010044892 | 0.15890554 | down | hsa:64333 |
| RND1 | 48.43547 | 72.71924 | 0.666061 | -0.58627 | 0.010056061 | 0.158931813 | down | hsa:27289 |
| APOBEC3G | 0.311795 | 5.1188 | 0.060912 | -4.03714 | 0.01017682 | 0.160185466 | down | hsa:60489 |
| CXCL3 | 27.16036 | 45.22536 | 0.600556 | -0.73563 | 0.01058652 | 0.164358905 | down | hsa:2921 |
| SOD3 | 16.54685 | 6.37474 | 2.59569 | 1.376118 | 0.01106615 | 0.168955457 | up | hsa:6649 |
| LOC105373098 | 8.421226 | 19.25995 | 0.43724 | -1.1935 | 0.011159684 | 0.169721018 | down |  |
| LOC402096 | 21.32556 | 9.538686 | 2.235692 | 1.160721 | 0.011683979 | 0.174360515 | up |  |
| STAG3L3 | 54.15791 | 88.42965 | 0.612441 | -0.70736 | 0.011696638 | 0.174360515 | down | hsa:442578 |
| SLC14A1 | 18.07576 | 7.403837 | 2.441404 | 1.287711 | 0.011806384 | 0.175138607 | up | hsa:6563 |
| APOBEC3F | 4.149547 | 12.62089 | 0.328784 | -1.60479 | 0.012670388 | 0.182629327 | down | hsa:200316 |
| MMP13 | 29.25648 | 59.64788 | 0.490487 | -1.02771 | 0.012843575 | 0.184164284 | down | hsa:4322 |
| GIMAP2 | 1.406779 | 7.370908 | 0.190856 | -2.38945 | 0.013065152 | 0.185455073 | down | hsa:26157 |
| EIF3LP2 | 51.00387 | 32.13231 | 1.587308 | 0.666582 | 0.013125113 | 0.185852246 | up |  |
| TTC30A | 70.94205 | 47.21877 | 1.502412 | 0.587281 | 0.013370676 | 0.187957666 | up | hsa:92104 |
| TXNDC16 | 43.27886 | 25.93248 | 1.668905 | 0.738902 | 0.013533638 | 0.188602521 | up | hsa:57544 |
| RPL7P19 | 59.01525 | 38.612 | 1.528418 | 0.612039 | 0.013846403 | 0.191306022 | up |  |
| HLA-K | 27.92968 | 45.21251 | 0.617742 | -0.69492 | 0.014485614 | 0.196885103 | down |  |
| RPS3AP37 | 5.60352 | 0.64082 | 8.744291 | 3.128341 | 0.014620016 | 0.198169909 | up |  |
| C15orf48 | 21.91909 | 37.17508 | 0.589618 | -0.76215 | 0.014637731 | 0.198270995 | down | hsa:84419 |
| LOC102723728 | 205.6297 | 427.8235 | 0.480641 | -1.05697 | 0.014955224 | 0.200989883 | down | hsa:102723728 |
| FER | 33.52258 | 16.97545 | 1.974768 | 0.981683 | 0.0152699 | 0.203240293 | up | hsa:2241 |
| ITGAM | 96.29128 | 148.8635 | 0.646843 | -0.62851 | 0.0152678 | 0.203240293 | down | hsa:3684 |
| ATP5J2P5 | 27.47773 | 44.76605 | 0.613807 | -0.70414 | 0.015525122 | 0.204850778 | down |  |
| SERF1B | 37.70817 | 14.55673 | 2.590428 | 1.373191 | 0.015551006 | 0.20489802 | up | hsa:728492 |
| KRT8P34 | 10.18284 | 22.79531 | 0.446708 | -1.1626 | 0.015605567 | 0.205010688 | down |  |
| LOC100420353 | 14.25419 | 5.468525 | 2.606588 | 1.382163 | 0.015585277 | 0.205010688 | up |  |
| SEPT6 | 15.84417 | 29.11704 | 0.544155 | -0.87791 | 0.016483479 | 0.212831771 | down | hsa:23157 |
| HNRNPA1P59 | 21.62348 | 36.94824 | 0.585237 | -0.77291 | 0.016685086 | 0.214574873 | down |  |
| ZNF25 | 14.71587 | 5.734455 | 2.566219 | 1.359644 | 0.017209943 | 0.219817844 | up | hsa:219749 |
| RORC | 22.25448 | 37.19195 | 0.598368 | -0.74089 | 0.017385864 | 0.220795901 | down | hsa:6097 |
| CPM | 20.90136 | 9.986126 | 2.09304 | 1.0656 | 0.017850032 | 0.223912905 | up | hsa:1368 |
| SLC8B1 | 45.1399 | 70.36282 | 0.641531 | -0.64041 | 0.017950789 | 0.224431308 | down | hsa:80024 |
| SNORD55 | 41.36647 | 25.11006 | 1.647406 | 0.720196 | 0.018115794 | 0.225850221 | up | hsa:26811 |
| SNORD3A | 406.0833 | 692.5851 | 0.58633 | -0.77022 | 0.018152386 | 0.225928831 | down | hsa:780851 |
| LOC105376752 | 31.47508 | 104.8699 | 0.300135 | -1.73632 | 0.018549238 | 0.229684955 | down |  |
| KRT78 | 0.698528 | 5.495296 | 0.127114 | -2.97581 | 0.018571989 | 0.229819452 | down | hsa:196374 |
| NFAM1 | 0.745721 | 5.429439 | 0.137348 | -2.8641 | 0.018661436 | 0.230631003 | down | hsa:150372 |
| LOC101929767 | 36.01551 | 20.76737 | 1.734236 | 0.7943 | 0.018675649 | 0.230659186 | up | hsa:101929767 |
| LOC100289320 | 2.067837 | 8.34298 | 0.247854 | -2.01244 | 0.018738016 | 0.230870733 | down |  |
| CD99 | 0.623589 | 7.326734 | 0.085111 | -3.5545 | 0.019241485 | 0.234648994 | down | hsa:4267 |
| ANKLE1 | 8.186687 | 18.30287 | 0.44729 | -1.16072 | 0.019309284 | 0.23503095 | down | hsa:126549 |
| LOC100130331 | 33.62018 | 51.74306 | 0.649752 | -0.62204 | 0.019693156 | 0.237608616 | down | hsa:100130331 |
| RPL21P51 | 36.36526 | 55.50098 | 0.655218 | -0.60995 | 0.019678509 | 0.237608616 | down |  |
| HDAC9 | 28.4612 | 45.51646 | 0.625295 | -0.67739 | 0.02058553 | 0.244559137 | down | hsa:9734 |
| LOC391020 | 16.7444 | 40.25247 | 0.415984 | -1.2654 | 0.020839079 | 0.245309722 | down |  |
| RPL21P94 | 22.29195 | 10.89234 | 2.046571 | 1.033209 | 0.02096166 | 0.24604336 | up |  |
| KANSL1-AS1 | 6.878443 | 16.11394 | 0.426863 | -1.22816 | 0.02147776 | 0.249141028 | down | hsa:644246 |
| RPL6P2 | 13.27162 | 5.164579 | 2.569738 | 1.361622 | 0.021547199 | 0.2493927 | up |  |
| RPS26P29 | 17.96567 | 31.78928 | 0.565149 | -0.8233 | 0.021648896 | 0.250120448 | down |  |
| HRASLS2 | 19.60052 | 33.08538 | 0.592422 | -0.7553 | 0.021748753 | 0.250525419 | down | hsa:54979 |
| LOC553103 | 3.512086 | 10.54931 | 0.332921 | -1.58675 | 0.021818634 | 0.250687504 | down | hsa:553103 |
| LOC105369863 | 4.616794 | 12.23208 | 0.377433 | -1.40571 | 0.022914587 | 0.258514549 | down |  |
| ATP10D | 6.576373 | 15.33123 | 0.428953 | -1.22111 | 0.023250551 | 0.2604845 | down | hsa:57205 |
| GABPAP | 47.36319 | 30.54003 | 1.550856 | 0.633065 | 0.023372659 | 0.261346531 | up |  |
| PARM1 | 22.64353 | 11.5594 | 1.958885 | 0.970033 | 0.023501334 | 0.26197075 | up | hsa:25849 |
| LOC101929057 | 1.383182 | 6.685379 | 0.206897 | -2.27302 | 0.023529259 | 0.262079921 | down | hsa:101929057 |
| TTYH2 | 1.247178 | 6.782559 | 0.18388 | -2.44316 | 0.023706124 | 0.263191084 | down | hsa:94015 |
| CD274 | 24.84737 | 39.81386 | 0.624088 | -0.68018 | 2.47E-02 | 2.70E-01 | down | hsa:29126 |
| MAMDC4 | 43.16734 | 27.62248 | 1.562761 | 0.644098 | 0.024726789 | 0.270132378 | up | hsa:158056 |
| SPATA3-AS1 | 7.677822 | 17.46198 | 0.439688 | -1.18545 | 0.02483099 | 0.271009714 | down | hsa:348761 |
| SCD5 | 1.308244 | 9.324693 | 0.140299 | -2.83342 | 0.025158178 | 0.272885763 | down | hsa:79966 |
| RBM43 | 17.18663 | 30.52611 | 0.563014 | -0.82876 | 0.025301494 | 0.273736814 | down | hsa:375287 |
| SRGAP2C | 27.66324 | 43.57552 | 0.634834 | -0.65555 | 0.025309863 | 0.273736814 | down | hsa:653464 |
| LINC00460 | 37.91545 | 23.04624 | 1.64519 | 0.718254 | 0.025705874 | 0.275884972 | up | hsa:728192 |
| SNORD3B-1 | 6.836825 | 15.77868 | 0.433295 | -1.20658 | 0.025873435 | 0.276455295 | down | hsa:26851 |
| ACOT1 | 32.0066 | 49.31096 | 0.649077 | -0.62354 | 0.025941532 | 0.276876796 | down | hsa:641371 |
| ENPP2 | 23.13941 | 12.28348 | 1.883783 | 0.913633 | 0.025929493 | 0.276876796 | up | hsa:5168 |
| SNORD28 | 17.86067 | 8.433468 | 2.117832 | 1.082588 | 0.026558097 | 0.280035838 | up | hsa:9300 |
| SCARNA22 | 0 | 3.236495 | 0 | -20 | 0.026618157 | 0.280226101 | down | hsa:677770 |
| RPS27P29 | 24.04799 | 12.5304 | 1.919172 | 0.940484 | 0.026707307 | 0.280442343 | up |  |
| RPL7AP40 | 4.82033 | 12.58234 | 0.383103 | -1.3842 | 0.026764674 | 0.280550102 | down |  |
| PPIAP33 | 23.3152 | 12.56226 | 1.855972 | 0.892175 | 0.027288084 | 0.284262165 | up |  |
| CBWD3 | 60.25597 | 38.44941 | 1.567149 | 0.648143 | 0.027367541 | 0.284554705 | up | hsa:445571 |
| PPIAP8 | 8.881066 | 18.62636 | 0.476801 | -1.06854 | 0.027491881 | 0.285080774 | down |  |
| TRNY | 7.572822 | 1.935311 | 3.912973 | 1.968265 | 0.028028088 | 0.288578279 | up | hsa:4579 |
| RPL7AP18 | 8.247753 | 18.19953 | 0.453185 | -1.14183 | 0.028170593 | 0.288940639 | down |  |
| FAM221B | 0.349264 | 4.219813 | 0.082768 | -3.59479 | 0.028790808 | 0.29304767 | down | hsa:392307 |
| LOC102723575 | 0.996449 | 5.785322 | 0.172238 | -2.53753 | 0.029410735 | 0.296237674 | down | hsa:102723575 |
| UBE2CP1 | 13.19253 | 5.442824 | 2.423839 | 1.277294 | 0.029597048 | 0.297648985 | up |  |
| LOC643454 | 24.3621 | 38.91032 | 0.626109 | -0.67551 | 0.029822486 | 0.2989828 | down |  |
| CDKN1C | 18.59109 | 8.904558 | 2.087817 | 1.061995 | 3.02E-02 | 3.01E-01 | up | hsa:1028 |
| ACTBL2 | 12.12529 | 4.84109 | 2.504661 | 1.324616 | 0.030224635 | 0.301450964 | up | hsa:345651 |
| RMRP | 28.41963 | 54.90129 | 0.51765 | -0.94995 | 0.030417427 | 0.302528035 | down | hsa:6023 |
| PHKG1 | 2.08171 | 7.740711 | 0.26893 | -1.8947 | 0.031139418 | 0.306619367 | down | hsa:5260 |
| LOC105377182 | 2.044241 | 7.630145 | 0.267916 | -1.90015 | 0.031833327 | 0.307677438 | down |  |
| MTMR9LP | 23.0506 | 12.54994 | 1.836709 | 0.877123 | 0.031800311 | 0.307677438 | up | hsa:339483 |
| TREML2 | 29.43499 | 44.87046 | 0.655999 | -0.60823 | 0.031728795 | 0.307677438 | down | hsa:79865 |
| ZGLP1 | 2.006771 | 7.688239 | 0.261018 | -1.93778 | 0.03173503 | 0.307677438 | down | hsa:100125288 |
| TFAP2A-AS1 | 8.224156 | 17.43574 | 0.471684 | -1.08411 | 0.031923147 | 0.308213042 | down | hsa:100130275 |
| LOC102723553 | 0.745721 | 7.040192 | 0.105923 | -3.23891 | 0.032005734 | 0.308702009 | down | hsa:102723553 |
| TMEM191C | 13.06625 | 2.284501 | 5.71952 | 2.515894 | 0.032180257 | 0.309612837 | up | hsa:645426 |
| WASH5P | 4.881396 | 12.15953 | 0.401446 | -1.31672 | 0.032835369 | 0.313476763 | down | hsa:375690 |
| LOC653653 | 12.34827 | 4.491365 | 2.749337 | 1.459084 | 0.032953125 | 0.313975645 | up | hsa:653653 |
| ANGPTL2 | 3.45102 | 10.00567 | 0.344906 | -1.53572 | 0.033972332 | 0.320630472 | down | hsa:23452 |
| FRMD3 | 6.25717 | 14.79482 | 0.42293 | -1.24151 | 0.03401692 | 0.32089461 | down | hsa:257019 |
| LOC105377381 | 7.663949 | 2.24595 | 3.412342 | 1.770762 | 0.03515386 | 0.327092319 | up |  |
| COL1A2 | 3.983481 | 0 | Inf | 20 | 0.035206465 | 0.327166231 | up | hsa:1278 |
| ARL4D | 23.00109 | 36.63144 | 0.627906 | -0.67138 | 0.035724507 | 0.330590539 | down | hsa:379 |
| ATP13A4 | 7.373435 | 1.896225 | 3.888481 | 1.959207 | 0.03661006 | 0.335301455 | up | hsa:84239 |
| CCDC15 | 37.01339 | 23.20875 | 1.594803 | 0.673379 | 0.036854268 | 0.336753828 | up | hsa:80071 |
| BCO1 | 22.23735 | 35.56942 | 0.625182 | -0.67765 | 0.037157293 | 0.338460564 | down | hsa:53630 |
| TMEM40 | 4.210613 | 10.90519 | 0.386111 | -1.37291 | 0.037318445 | 0.339608389 | down | hsa:55287 |
| INPP5J | 10.29199 | 19.93932 | 0.516166 | -0.95409 | 0.037478151 | 0.340510409 | down | hsa:27124 |
| HCAR3 | 8.857469 | 17.9593 | 0.493197 | -1.01976 | 0.038113059 | 0.343124038 | down | hsa:8843 |
| VAX2 | 2.653957 | 8.600612 | 0.308578 | -1.69629 | 0.038496845 | 0.34545289 | down | hsa:25806 |
| LOC101927673 | 28.68186 | 16.9883 | 1.68833 | 0.755597 | 0.038553086 | 0.345636641 | up | hsa:101927673 |
| SGMS1-AS1 | 5.956932 | 13.44224 | 0.44315 | -1.17413 | 0.03868464 | 0.346334138 | down |  |
| LOC643072 | 15.78542 | 7.403837 | 2.13206 | 1.092248 | 0.039134663 | 0.348170002 | up | hsa:643072 |
| SLC35G2 | 11.47164 | 21.20089 | 0.541093 | -0.88605 | 0.039406304 | 0.349396536 | down | hsa:80723 |
| SPN | 1.010322 | 5.468525 | 0.184752 | -2.43634 | 0.039394481 | 0.349396536 | down | hsa:6693 |
| ST7-AS1 | 0.996449 | 8.395451 | 0.118689 | -3.07474 | 0.039509586 | 0.349934969 | down | hsa:93653 |
| HMGB1P20 | 25.20725 | 14.51765 | 1.736318 | 0.796031 | 0.039624678 | 0.350368437 | up |  |
| MRPL45P1 | 0.996449 | 5.429974 | 0.183509 | -2.44608 | 0.039818254 | 0.350871128 | down |  |
| RPS17P9 | 3.739218 | 0.310639 | 12.03719 | 3.589427 | 0.039970448 | 0.351814212 | up |  |
| SRPK2P | 3.772538 | 10.26999 | 0.367336 | -1.44483 | 0.040092932 | 0.351814212 | down |  |
| LOC100190986 | 8.238029 | 2.588982 | 3.181956 | 1.669914 | 0.040376771 | 0.353141448 | up | hsa:100190986 |
| IL23A | 26.39104 | 40.59818 | 0.650055 | -0.62137 | 0.041146555 | 0.358091741 | down | hsa:51561 |
| RPS8P3 | 4.745391 | 11.84433 | 0.400646 | -1.3196 | 0.041413683 | 0.358801025 | down |  |
| ANKRD57P1 | 1.308244 | 6.037866 | 0.216673 | -2.20641 | 0.041757909 | 0.360812973 | down |  |
| FGF11 | 18.14655 | 8.905093 | 2.037772 | 1.026993 | 0.042202414 | 0.362702929 | up | hsa:2256 |
| PPIAP16 | 11.71265 | 21.56507 | 0.54313 | -0.88063 | 0.042128224 | 0.362702929 | down |  |
| ITIH6 | 3.437147 | 9.649252 | 0.356209 | -1.48921 | 0.042479417 | 0.363246415 | down | hsa:347365 |
| FBXO36 | 10.65839 | 19.64876 | 0.542446 | -0.88245 | 0.042766269 | 0.365441738 | down | hsa:130888 |
| BMS1P11 | 10.04358 | 3.818151 | 2.630483 | 1.395328 | 0.043042129 | 0.366886674 | up |  |
| APOD | 10.82677 | 20.48912 | 0.528416 | -0.92026 | 0.043449042 | 0.368022146 | down | hsa:347 |
| LOC105374775 | 4.484938 | 0.621277 | 7.2189 | 2.851779 | 0.043558934 | 0.368080588 | up |  |
| RASD1 | 24.75713 | 14.1797 | 1.745956 | 0.804017 | 0.043973316 | 0.370380107 | up | hsa:51655 |
| LOC101926889 | 4.41 | 0.64082 | 6.881804 | 2.782787 | 0.044072753 | 0.370859222 | up | hsa:101926889 |
| HCG27 | 5.183466 | 12.1539 | 0.426486 | -1.22943 | 0.045271391 | 0.376527844 | down | hsa:253018 |
| SLC25A6 | 0 | 207.5066 | 0 | -20 | 0.045716037 | 0.377657729 | down | hsa:293 |
| HADHAP1 | 25.39044 | 38.87632 | 0.653108 | -0.61461 | 0.045944785 | 0.379191613 | down |  |
| LOC202227 | 17.97722 | 31.25501 | 0.575179 | -0.79792 | 0.046008474 | 0.379555179 | down |  |
| LOC105376805 | 29.11994 | 17.60342 | 1.65422 | 0.726151 | 0.046391634 | 0.381251653 | up |  |
| FAM60BP | 51.80236 | 34.29849 | 1.51034 | 0.594873 | 0.047012525 | 0.384245248 | up |  |
| GPRASP1 | 8.545675 | 2.873385 | 2.974079 | 1.572443 | 0.046971207 | 0.384245248 | up | hsa:9737 |
| LOC100996741 | 2.837155 | 8.290508 | 0.342217 | -1.54702 | 0.047358001 | 0.38574918 | down | hsa:100996741 |
| SLC8A2 | 9.380207 | 17.95261 | 0.522498 | -0.9365 | 0.047661519 | 0.387079626 | down | hsa:6543 |
| XRCC6P1 | 29.3013 | 18.0174 | 1.626278 | 0.701574 | 0.047646806 | 0.387079626 | up |  |
| ACOXL | 4.810606 | 11.65604 | 0.412713 | -1.27679 | 0.04833464 | 0.390509489 | down | hsa:55289 |
| LOC100422076 | 0.745721 | 4.458972 | 0.167241 | -2.58 | 0.048900915 | 0.392603429 | down |  |
| RPL13P13 | 14.12234 | 6.749631 | 2.092313 | 1.065099 | 0.048987063 | 0.392729547 | up |  |
| MAST1 | 6.888167 | 1.896225 | 3.632568 | 1.86099 | 0.04902206 | 0.392847176 | up | hsa:22983 |
| LOC105372579 | 11.07518 | 20.24327 | 0.547104 | -0.87011 | 0.049510161 | 0.394969666 | down |  |
| LONRF2 | 37.18918 | 24.15351 | 1.539701 | 0.62265 | 0.04949407 | 0.394969666 | up | hsa:164832 |
| FCHO1 | 21.92555 | 34.35926 | 0.638127 | -0.64809 | 0.04980877 | 0.39555467 | down | hsa:23149 |
| FTH1P13 | 20.61316 | 11.11811 | 1.854016 | 0.890653 | 0.049783609 | 0.39555467 | up |  |
| LOC101930094 | 0.661058 | 4.478515 | 0.147607 | -2.76017 | 0.04964954 | 0.39555467 | down | hsa:101930094 |

**Table S15: Coexpression network analysis**

| **AccID** | **Degree-BMP** | **K-core-BMP** | **Degree-Control** | **K-core-Control** | **Dif_Degree** | **Dif_K-core** |
| --- | --- | --- | --- | --- | --- | --- |
| EPSTI1 | 16 | 12 | 1 | 1 | 15 | 11 |
| LOC102723728 | 13 | 12 | 5 | 3 | 8 | 9 |
| FTH1P13 | 11 | 9 | 0 | 0 | 11 | 9 |
| TMEM40 | 12 | 11 | 3 | 3 | 9 | 8 |
| LOC105369863 | 14 | 12 | 6 | 4 | 8 | 8 |
| RPL7P19 | 15 | 11 | 4 | 3 | 11 | 8 |
| LOC402096 | 16 | 12 | 6 | 4 | 10 | 8 |
| LOC101929767 | 13 | 11 | 5 | 4 | 8 | 7 |
| LOC391020 | 12 | 11 | 6 | 4 | 6 | 7 |
| HNRNPA3P7 | 14 | 11 | 7 | 4 | 7 | 7 |
| ANKRD57P1 | 14 | 11 | 5 | 4 | 9 | 7 |
| XAF1 | 12 | 11 | 5 | 4 | 7 | 7 |
| GPR89B | 12 | 11 | 5 | 4 | 7 | 7 |
| RPS3AP49 | 14 | 12 | 8 | 6 | 6 | 6 |
| CX3CL1 | 16 | 12 | 8 | 6 | 8 | 6 |
| BATF2 | 16 | 12 | 9 | 6 | 7 | 6 |
| TMEM140 | 14 | 12 | 7 | 6 | 7 | 6 |
| IFI44L | 14 | 9 | 4 | 3 | 10 | 6 |
| IFI30 | 10 | 9 | 6 | 4 | 4 | 5 |
| HADHAP1 | 11 | 7 | 2 | 2 | 9 | 5 |
| PAX4 | 11 | 7 | 2 | 2 | 9 | 5 |
| KRT8P34 | 13 | 12 | 9 | 7 | 4 | 5 |
| C17orf67 | 14 | 12 | 12 | 7 | 2 | 5 |
| CD68 | 12 | 9 | 6 | 4 | 6 | 5 |
| SAMD9L | 11 | 8 | 5 | 3 | 6 | 5 |
| HOXA-AS3 | 12 | 9 | 7 | 4 | 5 | 5 |
| CD99 | 8 | 8 | 4 | 3 | 4 | 5 |
| ISG15 | 10 | 9 | 5 | 4 | 5 | 5 |
| SNORD28 | 10 | 9 | 5 | 4 | 5 | 5 |
| ACO1 | 11 | 9 | 5 | 4 | 6 | 5 |
| SLC25A28 | 13 | 11 | 8 | 6 | 5 | 5 |
| BMP5 | 5 | 5 | 0 | 0 | 5 | 5 |
| HLA-C | 15 | 11 | 9 | 7 | 6 | 4 |
| SRPK2P | 10 | 7 | 4 | 3 | 6 | 4 |
| ARHGAP9 | 8 | 8 | 6 | 4 | 2 | 4 |
| RPLP2P2 | 12 | 9 | 8 | 5 | 4 | 4 |
| LOC101930094 | 15 | 12 | 13 | 8 | 2 | 4 |
| BTN3A1 | 9 | 7 | 3 | 3 | 6 | 4 |
| SCD5 | 14 | 11 | 11 | 7 | 3 | 4 |
| CNTNAP3P2 | 8 | 8 | 5 | 4 | 3 | 4 |
| LOC105373098 | 12 | 11 | 8 | 7 | 4 | 4 |
| IGHV3OR16-6 | 8 | 7 | 3 | 3 | 5 | 4 |
| MAST1 | 16 | 12 | 12 | 8 | 4 | 4 |
| RN7SK | 7 | 7 | 4 | 3 | 3 | 4 |
| CDH17 | 7 | 7 | 5 | 3 | 2 | 4 |
| SLFN5 | 10 | 7 | 4 | 3 | 6 | 4 |
| SAMHD1 | 6 | 6 | 2 | 2 | 4 | 4 |
| IFNL2 | 10 | 7 | 3 | 3 | 7 | 4 |
| GBP1 | 14 | 12 | 12 | 8 | 2 | 4 |
| COL1A2 | 4 | 4 | 0 | 0 | 4 | 4 |
| GABPAP | 8 | 7 | 4 | 4 | 4 | 3 |
| B2M | 9 | 7 | 5 | 4 | 4 | 3 |
| IL15RA | 11 | 6 | 4 | 3 | 7 | 3 |
| LOC553103 | 6 | 6 | 3 | 3 | 3 | 3 |
| RPL7AP50 | 11 | 7 | 7 | 4 | 4 | 3 |
| APOBEC3F | 9 | 7 | 6 | 4 | 3 | 3 |
| APOBEC3G | 8 | 8 | 5 | 5 | 3 | 3 |
| TRNF | 10 | 7 | 5 | 4 | 5 | 3 |
| TFAP2A-AS1 | 11 | 7 | 5 | 4 | 6 | 3 |
| RPL7AP18 | 10 | 6 | 5 | 3 | 5 | 3 |
| CSF1 | 8 | 7 | 5 | 4 | 3 | 3 |
| GMPR | 8 | 7 | 5 | 4 | 3 | 3 |
| MUC17 | 5 | 4 | 2 | 1 | 3 | 3 |
| CXCL1 | 11 | 7 | 5 | 4 | 6 | 3 |
| RND1 | 12 | 7 | 6 | 4 | 6 | 3 |
| TNFSF13B | 9 | 7 | 6 | 4 | 3 | 3 |
| SNORD55 | 7 | 5 | 2 | 2 | 5 | 3 |
| LOC105374236 | 10 | 7 | 5 | 4 | 5 | 3 |
| LOC105371082 | 8 | 7 | 6 | 4 | 2 | 3 |
| ITIH6 | 10 | 7 | 7 | 4 | 3 | 3 |
| USP18 | 9 | 7 | 6 | 4 | 3 | 3 |
| ZGLP1 | 7 | 4 | 1 | 1 | 6 | 3 |
| ST13P17 | 3 | 3 | 0 | 0 | 3 | 3 |
| IFITM1 | 10 | 8 | 7 | 6 | 3 | 2 |
| IFI35 | 7 | 5 | 3 | 3 | 4 | 2 |
| TXNDC16 | 6 | 5 | 4 | 3 | 2 | 2 |
| DHX58 | 13 | 7 | 7 | 5 | 6 | 2 |
| CKMT1B | 7 | 5 | 5 | 3 | 2 | 2 |
| RPL12P6 | 8 | 7 | 7 | 5 | 1 | 2 |
| HLA-J | 9 | 7 | 8 | 5 | 1 | 2 |
| HLA-H | 10 | 6 | 6 | 4 | 4 | 2 |
| HLA-E | 8 | 7 | 7 | 5 | 1 | 2 |
| LOC100101247 | 5 | 5 | 5 | 3 | 0 | 2 |
| FAM60BP | 10 | 7 | 6 | 5 | 4 | 2 |
| C3 | 6 | 4 | 3 | 2 | 3 | 2 |
| NAPSB | 10 | 7 | 7 | 5 | 3 | 2 |
| TREML2 | 11 | 8 | 8 | 6 | 3 | 2 |
| BTN3A3 | 6 | 5 | 4 | 3 | 2 | 2 |
| ANGPTL2 | 9 | 7 | 7 | 5 | 2 | 2 |
| CA11 | 8 | 7 | 6 | 5 | 2 | 2 |
| AKR1C1 | 6 | 5 | 4 | 3 | 2 | 2 |
| PLEKHA4 | 4 | 4 | 2 | 2 | 2 | 2 |
| TRNY | 11 | 7 | 7 | 5 | 4 | 2 |
| ST7-AS1 | 9 | 7 | 6 | 5 | 3 | 2 |
| GAPDHP32 | 8 | 6 | 5 | 4 | 3 | 2 |
| CSF3 | 8 | 7 | 6 | 5 | 2 | 2 |
| HNRNPA1P59 | 12 | 9 | 8 | 7 | 4 | 2 |
| LOC123862 | 8 | 7 | 7 | 5 | 1 | 2 |
| TAP1 | 4 | 4 | 2 | 2 | 2 | 2 |
| FRMD3 | 6 | 6 | 6 | 4 | 0 | 2 |
| SNORD3A | 12 | 7 | 6 | 5 | 6 | 2 |
| RASGRP3 | 6 | 5 | 4 | 3 | 2 | 2 |
| CCDC15 | 6 | 6 | 6 | 4 | 0 | 2 |
| ITGAM | 7 | 6 | 7 | 4 | 0 | 2 |
| SMG1P1 | 6 | 6 | 5 | 4 | 1 | 2 |
| LOC105377182 | 10 | 9 | 11 | 7 | -1 | 2 |
| SEPT4 | 9 | 7 | 7 | 5 | 2 | 2 |
| PATL2 | 8 | 7 | 7 | 5 | 1 | 2 |
| EIF3LP2 | 6 | 6 | 7 | 4 | -1 | 2 |
| LOC643454 | 10 | 7 | 6 | 5 | 4 | 2 |
| BCO1 | 8 | 7 | 6 | 5 | 2 | 2 |
| GBP4 | 7 | 6 | 7 | 4 | 0 | 2 |
| MX2 | 5 | 5 | 4 | 3 | 1 | 2 |
| LOC101927673 | 7 | 6 | 4 | 4 | 3 | 2 |
| ANKLE1 | 8 | 7 | 11 | 6 | -3 | 1 |
| IFIT3 | 9 | 7 | 7 | 6 | 2 | 1 |
| SRGAP2C | 7 | 5 | 6 | 4 | 1 | 1 |
| TNFSF10 | 5 | 4 | 4 | 3 | 1 | 1 |
| TRIM22 | 11 | 7 | 9 | 6 | 2 | 1 |
| FLT3LG | 8 | 7 | 9 | 6 | -1 | 1 |
| CPT1B | 5 | 4 | 4 | 3 | 1 | 1 |
| RPS14P8 | 7 | 5 | 7 | 4 | 0 | 1 |
| ZNF25 | 8 | 7 | 7 | 6 | 1 | 1 |
| HLA-S | 7 | 6 | 8 | 5 | -1 | 1 |
| HLA-W | 9 | 7 | 9 | 6 | 0 | 1 |
| RPL32P21 | 8 | 7 | 10 | 6 | -2 | 1 |
| SRP14-AS1 | 8 | 7 | 7 | 6 | 1 | 1 |
| CDKN1C | 6 | 5 | 7 | 4 | -1 | 1 |
| TRIM5 | 9 | 8 | 10 | 7 | -1 | 1 |
| SGMS1-AS1 | 5 | 4 | 5 | 3 | 0 | 1 |
| ARHGAP23P1 | 5 | 5 | 7 | 4 | -2 | 1 |
| LOC100190986 | 4 | 4 | 4 | 3 | 0 | 1 |
| FOSB | 5 | 5 | 5 | 4 | 0 | 1 |
| TMEM229B | 10 | 9 | 10 | 8 | 0 | 1 |
| RORC | 8 | 6 | 5 | 5 | 3 | 1 |
| CCDC28B | 8 | 7 | 10 | 6 | -2 | 1 |
| CTGF | 5 | 5 | 4 | 4 | 1 | 1 |
| ZBP1 | 14 | 11 | 14 | 10 | 0 | 1 |
| CMPK2 | 6 | 4 | 3 | 3 | 3 | 1 |
| HCAR3 | 6 | 5 | 4 | 4 | 2 | 1 |
| UBD | 5 | 4 | 6 | 3 | -1 | 1 |
| PI3 | 4 | 4 | 3 | 3 | 1 | 1 |
| TXNP4 | 7 | 5 | 5 | 4 | 2 | 1 |
| TNFAIP3 | 5 | 5 | 5 | 4 | 0 | 1 |
| CXCL3 | 6 | 6 | 6 | 5 | 0 | 1 |
| TMEM191C | 9 | 7 | 8 | 6 | 1 | 1 |
| SEPT6 | 9 | 7 | 10 | 6 | -1 | 1 |
| RPS26P28 | 7 | 4 | 5 | 3 | 2 | 1 |
| ZNF385C | 5 | 4 | 3 | 3 | 2 | 1 |
| LOC100996741 | 6 | 6 | 5 | 5 | 1 | 1 |
| PHKG1 | 5 | 5 | 6 | 4 | -1 | 1 |
| IFITM3 | 6 | 4 | 4 | 4 | 2 | 0 |
| C4B | 8 | 5 | 6 | 5 | 2 | 0 |
| HERC5 | 5 | 4 | 8 | 4 | -3 | 0 |
| C4A | 7 | 5 | 6 | 5 | 1 | 0 |
| HLA-L | 9 | 7 | 10 | 7 | -1 | 0 |
| HLA-G | 5 | 3 | 5 | 3 | 0 | 0 |
| CFB | 10 | 7 | 10 | 7 | 0 | 0 |
| SNORD3B-1 | 6 | 4 | 5 | 4 | 1 | 0 |
| TTYH2 | 8 | 8 | 9 | 8 | -1 | 0 |
| FAM221B | 7 | 5 | 8 | 5 | -1 | 0 |
| RPS8P3 | 7 | 6 | 8 | 6 | -1 | 0 |
| TRIM52-AS1 | 7 | 4 | 6 | 4 | 1 | 0 |
| RPL7AP40 | 7 | 6 | 9 | 6 | -2 | 0 |
| AHCYP4 | 4 | 4 | 6 | 4 | -2 | 0 |
| HEPACAM2 | 4 | 3 | 4 | 3 | 0 | 0 |
| LOC653653 | 7 | 4 | 6 | 4 | 1 | 0 |
| GPRASP1 | 11 | 6 | 7 | 6 | 4 | 0 |
| BTN3A2 | 11 | 7 | 10 | 7 | 1 | 0 |
| DUOX1 | 7 | 4 | 7 | 4 | 0 | 0 |
| SUSD3 | 5 | 4 | 6 | 4 | -1 | 0 |
| IL32 | 3 | 3 | 3 | 3 | 0 | 0 |
| CCL20 | 9 | 7 | 9 | 7 | 0 | 0 |
| MIB2 | 5 | 4 | 4 | 4 | 1 | 0 |
| PSMB8 | 6 | 4 | 5 | 4 | 1 | 0 |
| TTC30A | 4 | 3 | 4 | 3 | 0 | 0 |
| GIMAP2 | 7 | 5 | 6 | 5 | 1 | 0 |
| SLC8A2 | 5 | 4 | 4 | 4 | 1 | 0 |
| LOC442309 | 8 | 5 | 7 | 5 | 1 | 0 |
| ENPP2 | 8 | 5 | 9 | 5 | -1 | 0 |
| RPL21P94 | 5 | 3 | 3 | 3 | 2 | 0 |
| HRASLS2 | 6 | 4 | 6 | 4 | 0 | 0 |
| LOC102723575 | 9 | 7 | 8 | 7 | 1 | 0 |
| MR1 | 5 | 4 | 5 | 4 | 0 | 0 |
| CD274 | 6 | 3 | 3 | 3 | 3 | 0 |
| PARM1 | 10 | 7 | 9 | 7 | 1 | 0 |
| LOC100130331 | 10 | 8 | 11 | 8 | -1 | 0 |
| LONRF2 | 5 | 4 | 6 | 4 | -1 | 0 |
| TXNP5 | 6 | 4 | 4 | 4 | 2 | 0 |
| CXCL10 | 5 | 4 | 6 | 4 | -1 | 0 |
| CXCL11 | 4 | 4 | 4 | 4 | 0 | 0 |
| C6orf222 | 7 | 4 | 6 | 4 | 1 | 0 |
| RPL10P3 | 9 | 5 | 7 | 5 | 2 | 0 |
| TNFRSF1B | 4 | 4 | 5 | 4 | -1 | 0 |
| ACTBL2 | 5 | 4 | 4 | 4 | 1 | 0 |
| LOC100422076 | 7 | 5 | 7 | 5 | 0 | 0 |
| FRRS1 | 4 | 3 | 5 | 3 | -1 | 0 |
| RPL32P7 | 6 | 4 | 4 | 4 | 2 | 0 |
| HCG27 | 7 | 6 | 6 | 6 | 1 | 0 |
| LOC100420353 | 6 | 4 | 6 | 4 | 0 | 0 |
| ACKR4 | 5 | 4 | 6 | 4 | -1 | 0 |
| RBM43 | 8 | 7 | 9 | 7 | -1 | 0 |
| HCP5 | 3 | 3 | 6 | 4 | -3 | -1 |
| IFITM2 | 8 | 7 | 12 | 8 | -4 | -1 |
| RPL36AP19 | 9 | 7 | 11 | 8 | -2 | -1 |
| VSIG1 | 6 | 4 | 8 | 5 | -2 | -1 |
| LOC105374327 | 9 | 7 | 10 | 8 | -1 | -1 |
| TRIM21 | 7 | 6 | 8 | 7 | -1 | -1 |
| VRTN | 6 | 6 | 9 | 7 | -3 | -1 |
| HMGB1P20 | 9 | 7 | 9 | 8 | 0 | -1 |
| HLA-F | 5 | 3 | 4 | 4 | 1 | -1 |
| TAPBPL | 4 | 3 | 6 | 4 | -2 | -1 |
| LOC100101246 | 6 | 3 | 4 | 4 | 2 | -1 |
| PARP12 | 7 | 5 | 8 | 6 | -1 | -1 |
| LOC105376752 | 5 | 4 | 5 | 5 | 0 | -1 |
| RAET1L | 6 | 5 | 9 | 6 | -3 | -1 |
| IFIH1 | 5 | 5 | 7 | 6 | -2 | -1 |
| TRIM6 | 9 | 7 | 8 | 8 | 1 | -1 |
| MAMDC4 | 5 | 3 | 5 | 4 | 0 | -1 |
| SLC35G2 | 4 | 3 | 6 | 4 | -2 | -1 |
| LOC105376805 | 10 | 7 | 12 | 8 | -2 | -1 |
| MMP13 | 8 | 6 | 10 | 7 | -2 | -1 |
| LOC105372579 | 6 | 5 | 11 | 6 | -5 | -1 |
| PPIAP16 | 7 | 5 | 9 | 6 | -2 | -1 |
| LOC100420067 | 11 | 7 | 11 | 8 | 0 | -1 |
| APOL3 | 9 | 7 | 9 | 8 | 0 | -1 |
| CD74 | 6 | 5 | 8 | 6 | -2 | -1 |
| STAT2 | 7 | 6 | 12 | 7 | -5 | -1 |
| CAPS | 8 | 6 | 8 | 7 | 0 | -1 |
| LAMP3 | 7 | 5 | 8 | 6 | -1 | -1 |
| SERF1B | 10 | 6 | 8 | 7 | 2 | -1 |
| LOC101926889 | 6 | 5 | 9 | 6 | -3 | -1 |
| FBXO36 | 4 | 4 | 6 | 5 | -2 | -1 |
| LINC00460 | 8 | 7 | 10 | 8 | -2 | -1 |
| ARHGAP11B | 6 | 4 | 7 | 5 | -1 | -1 |
| SPN | 4 | 3 | 6 | 4 | -2 | -1 |
| ACOXL | 6 | 4 | 7 | 5 | -1 | -1 |
| MDK | 4 | 3 | 5 | 4 | -1 | -1 |
| XRCC6P1 | 6 | 5 | 8 | 6 | -2 | -1 |
| IL2RG | 4 | 3 | 4 | 4 | 0 | -1 |
| IFI16 | 4 | 4 | 6 | 5 | -2 | -1 |
| CCL5 | 7 | 6 | 8 | 7 | -1 | -1 |
| BEND7 | 5 | 5 | 9 | 6 | -4 | -1 |
| IFNL3 | 8 | 5 | 8 | 6 | 0 | -1 |
| SOD3 | 11 | 7 | 15 | 8 | -4 | -1 |
| SPATA3-AS1 | 4 | 3 | 5 | 4 | -1 | -1 |
| LOC101929057 | 9 | 7 | 15 | 8 | -6 | -1 |
| RPS27P29 | 7 | 4 | 9 | 5 | -2 | -1 |
| PPIAP8 | 5 | 5 | 13 | 7 | -8 | -2 |
| IFIT2 | 7 | 6 | 13 | 8 | -6 | -2 |
| MT2A | 4 | 4 | 8 | 6 | -4 | -2 |
| HMGN2P40 | 5 | 4 | 9 | 6 | -4 | -2 |
| HLA-K | 6 | 4 | 8 | 6 | -2 | -2 |
| LOC105377381 | 7 | 4 | 12 | 6 | -5 | -2 |
| APOD | 4 | 4 | 9 | 6 | -5 | -2 |
| RPL32P27 | 6 | 4 | 6 | 6 | 0 | -2 |
| GSDMB | 5 | 4 | 7 | 6 | -2 | -2 |
| RPL6P2 | 8 | 6 | 11 | 8 | -3 | -2 |
| RPS3AP37 | 6 | 4 | 9 | 6 | -3 | -2 |
| C9orf91 | 6 | 5 | 10 | 7 | -4 | -2 |
| RPLP2P5 | 3 | 3 | 7 | 5 | -4 | -2 |
| MTMR9LP | 6 | 6 | 10 | 8 | -4 | -2 |
| NFAM1 | 7 | 5 | 10 | 7 | -3 | -2 |
| IFI6 | 3 | 3 | 8 | 5 | -5 | -2 |
| SLC8B1 | 8 | 7 | 13 | 9 | -5 | -2 |
| MRPL45P1 | 9 | 7 | 11 | 9 | -2 | -2 |
| GAPDHP62 | 4 | 3 | 7 | 5 | -3 | -2 |
| BMS1P11 | 6 | 4 | 6 | 6 | 0 | -2 |
| ISG20 | 4 | 3 | 7 | 5 | -3 | -2 |
| FER | 4 | 4 | 6 | 6 | -2 | -2 |
| IFITM9P | 6 | 5 | 10 | 7 | -4 | -2 |
| OASL | 6 | 5 | 10 | 7 | -4 | -2 |
| CASP1 | 5 | 5 | 11 | 7 | -6 | -2 |
| RPL21P34 | 5 | 4 | 8 | 6 | -3 | -2 |
| RPL21P51 | 5 | 5 | 8 | 7 | -3 | -2 |
| TMED10P1 | 7 | 4 | 9 | 6 | -2 | -2 |
| KANSL1-AS1 | 7 | 5 | 9 | 7 | -2 | -2 |
| C15orf48 | 6 | 4 | 9 | 6 | -3 | -2 |
| ETV7 | 5 | 4 | 6 | 6 | -1 | -2 |
| OAS1 | 8 | 5 | 11 | 7 | -3 | -2 |
| ZBTB41 | 7 | 4 | 10 | 6 | -3 | -2 |
| DSPA2D | 8 | 4 | 9 | 6 | -1 | -2 |
| FCHO1 | 7 | 5 | 8 | 7 | -1 | -2 |
| PML | 6 | 4 | 10 | 6 | -4 | -2 |
| LOC202227 | 5 | 5 | 8 | 7 | -3 | -2 |
| PPIAP33 | 4 | 3 | 9 | 6 | -5 | -3 |
| LOC105372935 | 4 | 4 | 11 | 7 | -7 | -3 |
| UBE2L6 | 4 | 4 | 9 | 7 | -5 | -3 |
| RASSF4 | 4 | 4 | 10 | 7 | -6 | -3 |
| HLA-B | 4 | 3 | 8 | 6 | -4 | -3 |
| LOC728048 | 4 | 4 | 10 | 7 | -6 | -3 |
| IL23A | 8 | 7 | 15 | 10 | -7 | -3 |
| ACOT1 | 4 | 3 | 10 | 6 | -6 | -3 |
| PLA1A | 7 | 5 | 10 | 8 | -3 | -3 |
| LOC441880 | 3 | 3 | 8 | 6 | -5 | -3 |
| INPP5J | 5 | 4 | 9 | 7 | -4 | -3 |
| PTPRN2 | 3 | 3 | 6 | 6 | -3 | -3 |
| NOSTRIN | 5 | 4 | 10 | 7 | -5 | -3 |
| COL9A2 | 5 | 4 | 11 | 7 | -6 | -3 |
| PIGR | 5 | 4 | 10 | 7 | -5 | -3 |
| C5orf56 | 4 | 4 | 9 | 7 | -5 | -3 |
| TMSB10P2 | 6 | 4 | 10 | 7 | -4 | -3 |
| LOC105374775 | 6 | 5 | 12 | 8 | -6 | -3 |
| TAP2 | 5 | 3 | 9 | 6 | -4 | -3 |
| KRT78 | 7 | 5 | 11 | 8 | -4 | -3 |
| PSMB8-AS1 | 5 | 4 | 10 | 7 | -5 | -3 |
| SLC15A3 | 2 | 1 | 5 | 4 | -3 | -3 |
| RPL21P101 | 5 | 5 | 8 | 8 | -3 | -3 |
| RPL7P32 | 4 | 4 | 10 | 7 | -6 | -3 |
| ARHGAP33 | 7 | 5 | 13 | 8 | -6 | -3 |
| CPM | 9 | 7 | 12 | 10 | -3 | -3 |
| TNFSF12-TNFSF13 | 4 | 4 | 10 | 7 | -6 | -3 |
| RMRP | 12 | 7 | 12 | 10 | 0 | -3 |
| LOC100289320 | 3 | 3 | 9 | 6 | -6 | -3 |
| WASH5P | 4 | 4 | 8 | 7 | -4 | -3 |
| ARL4D | 6 | 5 | 8 | 8 | -2 | -3 |
| HSH2D | 4 | 3 | 11 | 6 | -7 | -3 |
| OAS2 | 6 | 3 | 9 | 6 | -3 | -3 |
| USP41 | 5 | 5 | 10 | 8 | -5 | -3 |
| UBE2CP1 | 6 | 5 | 10 | 8 | -4 | -3 |
| ATP10D | 3 | 3 | 7 | 6 | -4 | -3 |
| IFNL1 | 6 | 4 | 9 | 7 | -3 | -3 |
| LOC102723553 | 7 | 5 | 10 | 8 | -3 | -3 |
| PRKACB | 9 | 5 | 13 | 9 | -4 | -4 |
| TRANK1 | 3 | 3 | 9 | 7 | -6 | -4 |
| BLACAT1 | 5 | 4 | 13 | 8 | -8 | -4 |
| STAG3L3 | 6 | 4 | 12 | 8 | -6 | -4 |
| SECTM1 | 6 | 4 | 12 | 8 | -6 | -4 |
| RPL13P13 | 4 | 3 | 12 | 7 | -8 | -4 |
| CTSS | 5 | 4 | 11 | 8 | -6 | -4 |
| CTSO | 6 | 4 | 9 | 8 | -3 | -4 |
| GDPD3 | 6 | 4 | 10 | 8 | -4 | -4 |
| LGALS9DP | 5 | 4 | 10 | 8 | -5 | -4 |
| TYMP | 3 | 3 | 10 | 7 | -7 | -4 |
| KRT13 | 4 | 3 | 8 | 7 | -4 | -4 |
| DHRS2 | 4 | 4 | 11 | 8 | -7 | -4 |
| PSMB9 | 5 | 4 | 11 | 8 | -6 | -4 |
| RPS17P9 | 5 | 4 | 12 | 8 | -7 | -4 |
| RPL21P86 | 7 | 5 | 10 | 9 | -3 | -4 |
| UBA7 | 3 | 3 | 8 | 7 | -5 | -4 |
| BCL2L14 | 4 | 3 | 8 | 7 | -4 | -4 |
| CBWD3 | 6 | 4 | 13 | 8 | -7 | -4 |
| RPS26P29 | 8 | 4 | 12 | 8 | -4 | -4 |
| NLRC5 | 7 | 5 | 14 | 9 | -7 | -4 |
| HDAC9 | 5 | 4 | 12 | 8 | -7 | -4 |
| RASD1 | 9 | 5 | 11 | 9 | -2 | -4 |
| MX1 | 4 | 4 | 12 | 8 | -8 | -4 |
| RSAD2 | 6 | 5 | 13 | 10 | -7 | -5 |
| PLAC8 | 5 | 3 | 10 | 8 | -5 | -5 |
| FN1 | 3 | 3 | 15 | 8 | -12 | -5 |
| LAP3P2 | 4 | 4 | 10 | 9 | -6 | -5 |
| MT2P1 | 6 | 4 | 13 | 9 | -7 | -5 |
| LOC643072 | 5 | 5 | 13 | 10 | -8 | -5 |
| NUPR1 | 4 | 3 | 13 | 8 | -9 | -5 |
| SLC14A1 | 8 | 5 | 13 | 10 | -5 | -5 |
| LOC100419583 | 3 | 3 | 12 | 8 | -9 | -5 |
| ATP13A4 | 5 | 3 | 12 | 8 | -7 | -5 |
| IFIT1 | 3 | 3 | 13 | 9 | -10 | -6 |
| ZSCAN31 | 1 | 1 | 10 | 7 | -9 | -6 |
| FGF11 | 4 | 4 | 13 | 10 | -9 | -6 |
| RTP4 | 4 | 3 | 10 | 9 | -6 | -6 |
| ATP1A3 | 6 | 4 | 12 | 10 | -6 | -6 |
| ACTBP12 | 7 | 4 | 12 | 10 | -5 | -6 |
| VAX2 | 0 | 0 | 6 | 6 | -6 | -6 |
| ATP5J2P5 | 5 | 3 | 13 | 10 | -8 | -7 |
| SCARNA22 | 0 | 0 | 8 | 7 | -8 | -7 |
| SLC25A6 | 0 | 0 | 12 | 8 | -12 | -8 |
