# Supplementary material for: Alteration of tumor suppressor BMP5 in sporadic colorectal cancer: a genomic and transcriptomic profiling based study
Source: Mol Cancer. 2018 Dec 20;17:176. doi: 10.1186/s12943-018-0925-7 (PMC6302470; doi:10.1186/s12943-018-0925-7)
Supplement: Supplementary file 2 — Figure S1. Exome capture and sequencing of 3 sporadic colorectal cancer samples. Figure S2. Detection of BMP5 somatic mutations in initial exome sequencing and expanded deep sequencing samples. Figure S3. Alignment of BMP5 in different species. The nonsynonymous mutation are marked and indicated with arrows, all seven amino acids are highly conserved among different species. Sus: Sus scrofa, Dr.: Zebra fish. Figure S4. Kaplan–Meier analysis of overall survival according to high or low BMP5 expression in six tumor types (Log rank test). Figure S5. Immunofluorescence staining of BMP5 in NCM460 (A) and SW480 (B) cells. Figure S6. Pearson correlation analysis between BMP5 and miR-32, miR-655. The data were analyzed using TCGA CRC tumor samples and stage IV samples. Figure S7. Overexpression and knockdown efficiency validation in HT-29 and SW480 cells. (A) Overexpression efficiency of BMP5 in HT-29 cells. Tumor proliferation marker PCNA was checked. (B) Overexpression and knockdown efficiency of BMP5 in SW480. Tumor proliferation marker PCNA and migration marker (MMP2 and MMP9) were detected. MMP9 showed no significant difference. Figure S8. Cell apoptosis of BMP5 in HT-29 and SW480 cells. The data are showed as mean ± sem. Figure S9. Pearson correlation analysis between BMP5 and EMT markers. (A) Correlation analysis in tumor samples. (B)Correlation of fold change (Log2(Tumor/Normal)) between BMP5 and EMT markers. (C) Correlation between BMP5 and E-cadherin in six tumor types. Figure S10. Coexpression network of control HT-29 (A) and BMP5-expressing HT-29 cells (B). Figure S11. Correlation analysis between BMP5 and SMAD or STAT signaling in lung, breast, esophagus, stomach, liver and pancreatic cancer. (DOC 10825 kb) [file 12943_2018_925_MOESM2_ESM.doc]

**Supplementary Figures**

**
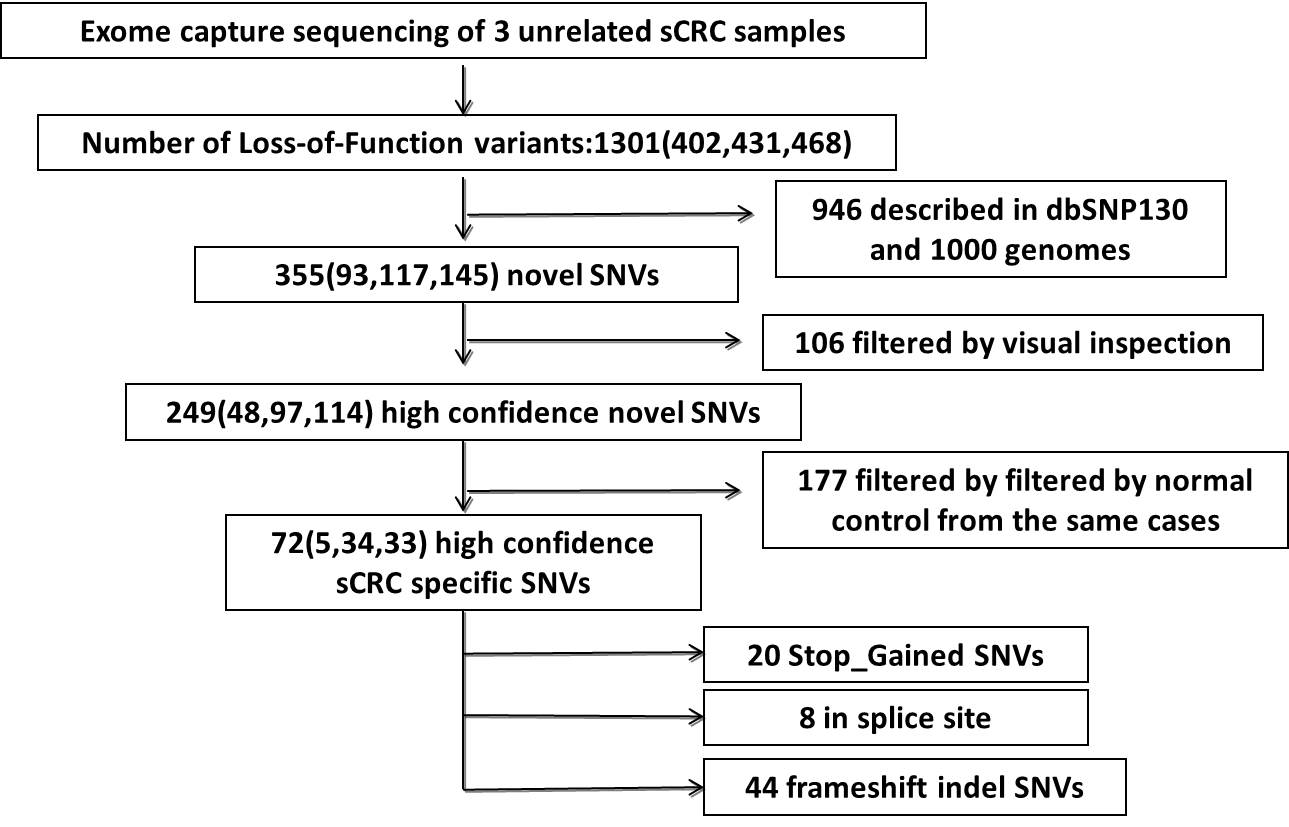
**

**Figure S1.** **Exome capture and sequencing of 3 sporadic colorectal cancer samples**


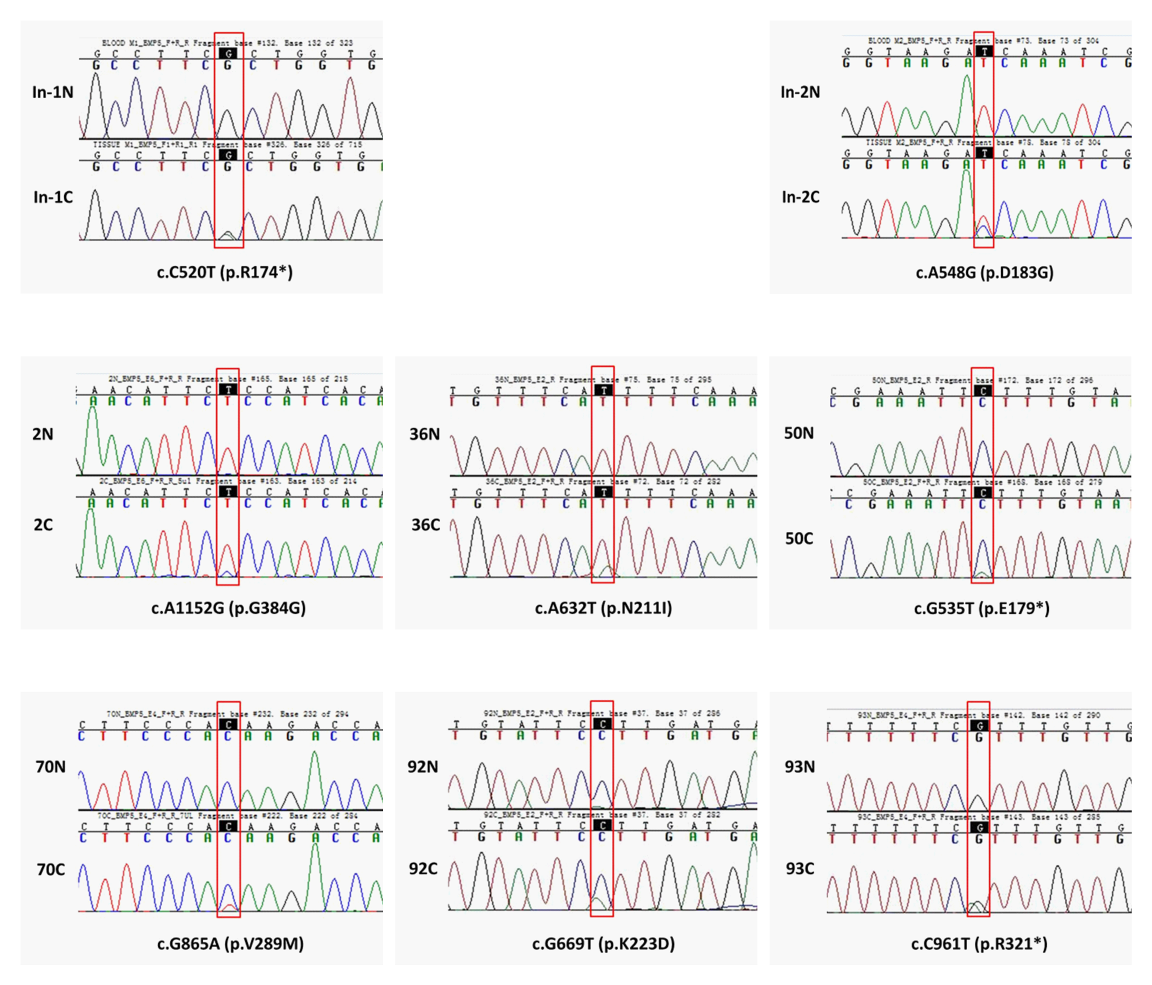


**Figure S2. Detection of *BMP5* somatic mutations in initial exome sequencing and expanded deep sequencing samples**


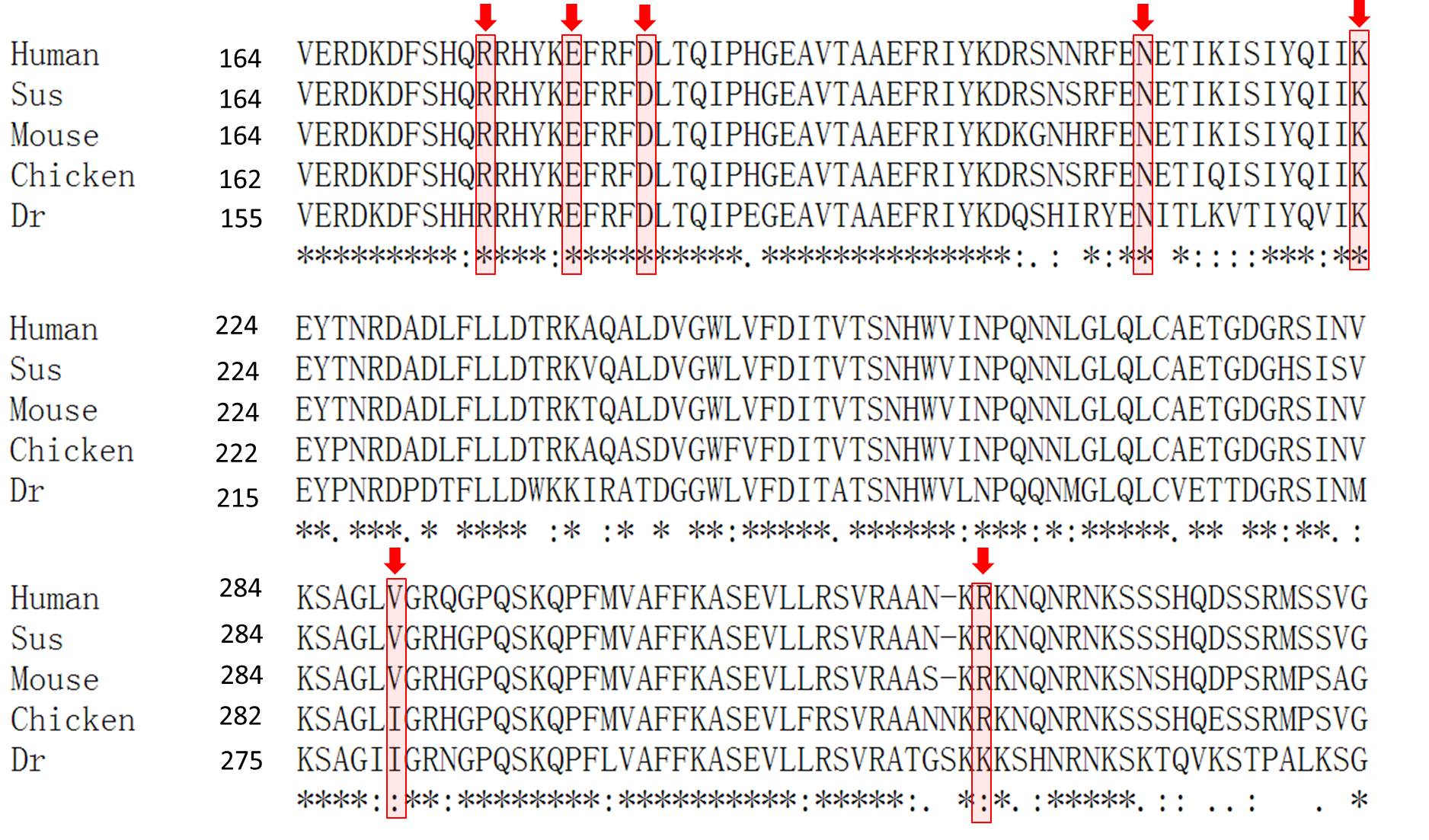


**Figure S3. Alignment of BMP5 in different species.** The nonsynonymous mutation are marked and indicated with arrows, all seven amino acids are highly conserved among different species. Sus: Sus scrofa, Dr: Zebra fish.


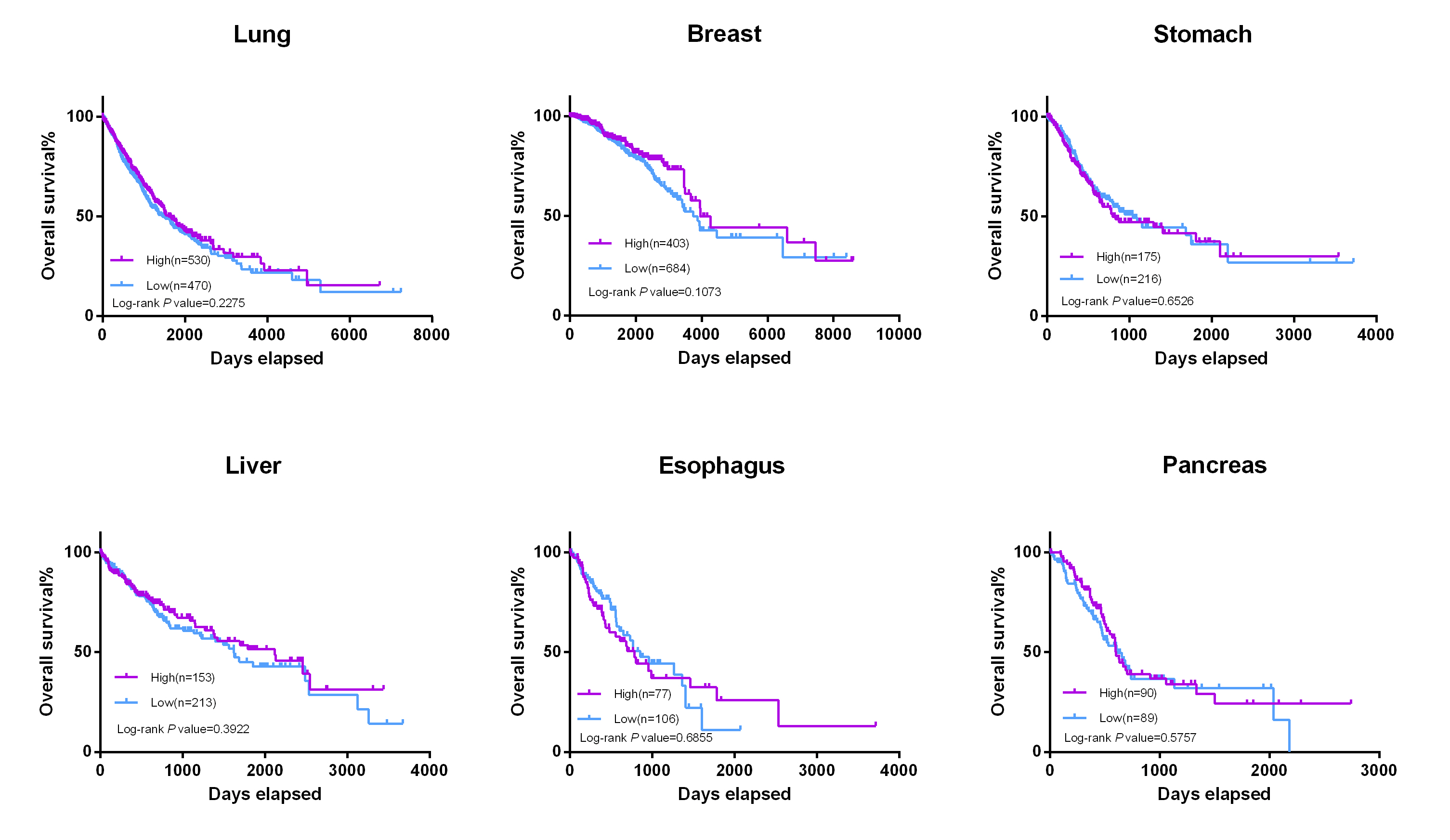


**Figure S4. Kaplan–Meier analysis of overall survival according to high or low BMP5 expression in six tumor types (Log rank test).**

**
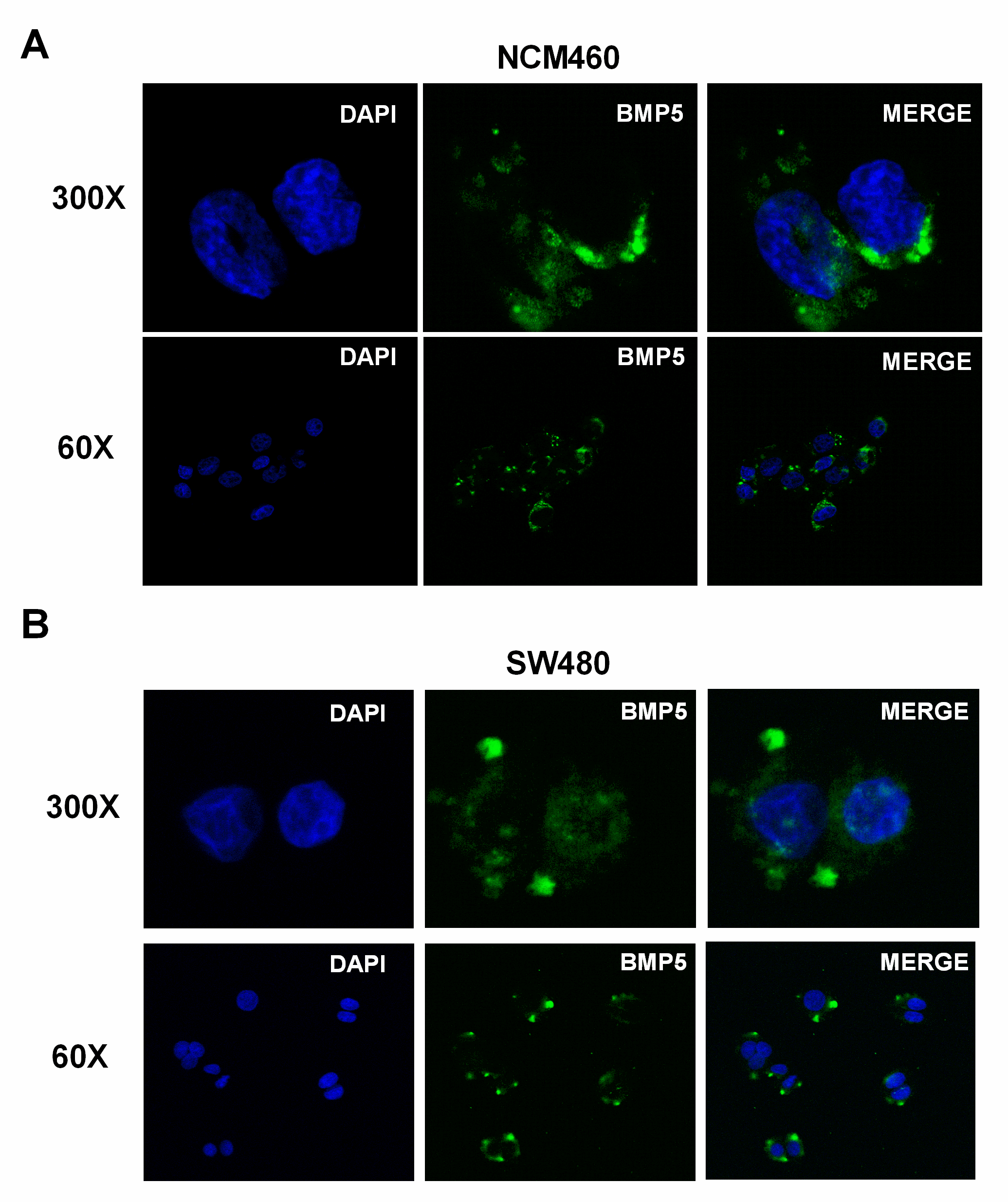
**

**Figure S5. Immunofluorescence staining of BMP5 in NCM460 (A) and SW480 (B) cells.**


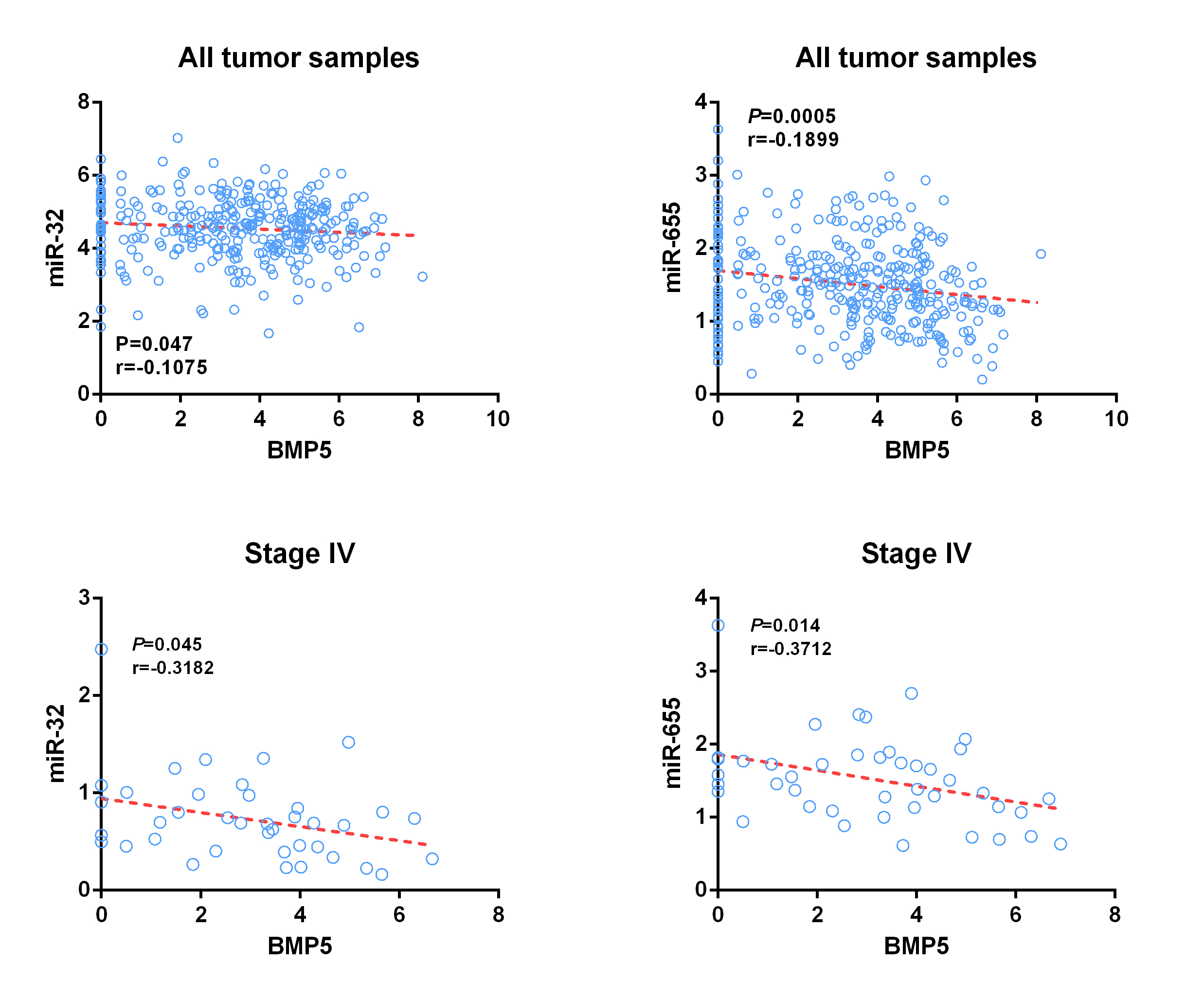


**Figure S6. Pearson correlation analysis between BMP5 and miR-32, miR-655.** The data were analyzed using TCGA CRC tumor samples and stage IV samples.


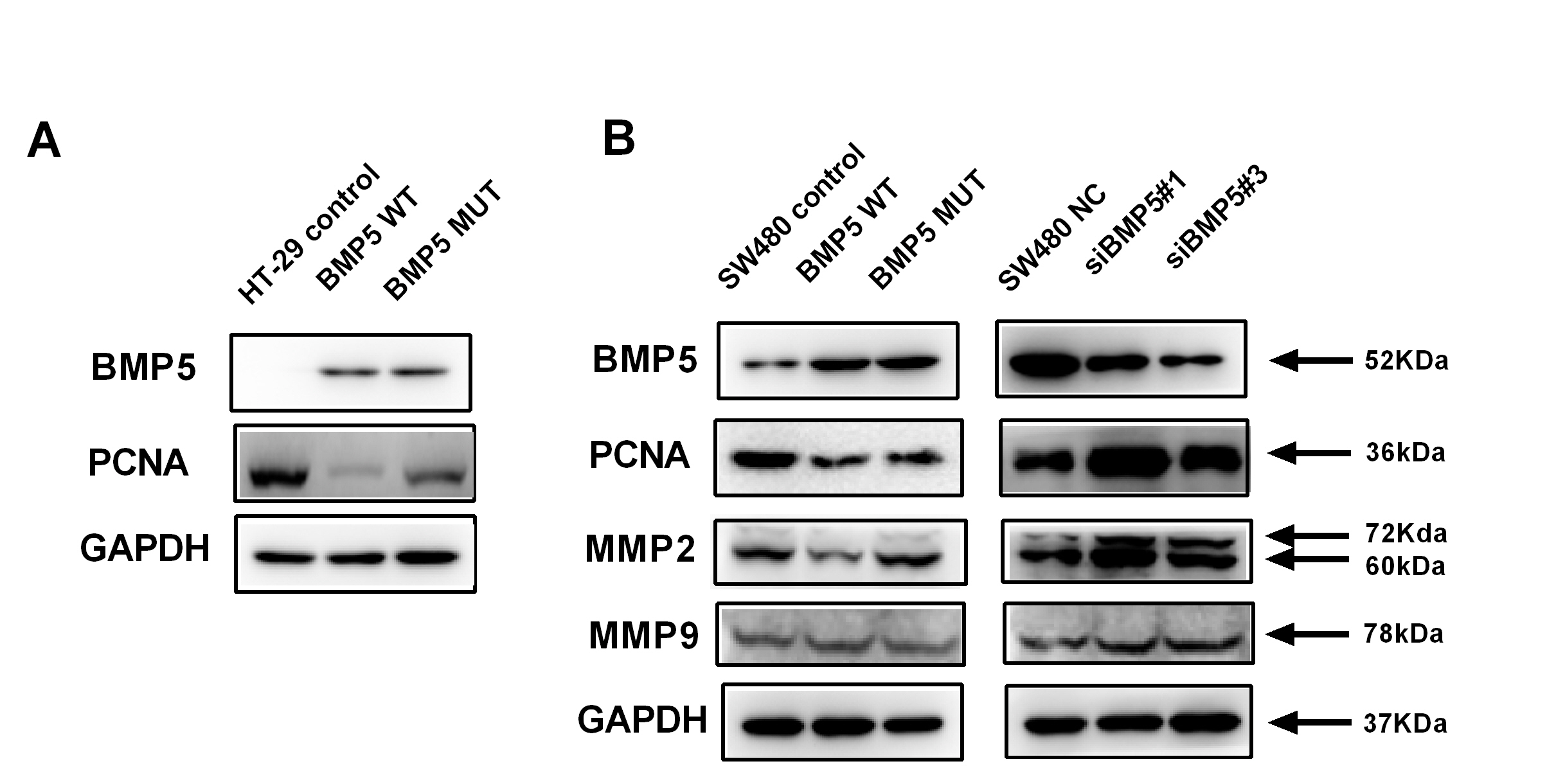


**Figure S7. Overexpression and knockdown efficiency validation in HT-29 and SW480 cells.** (A) Overexpression efficiency of BMP5 in HT-29 cells. Tumor proliferation marker PCNA was checked. (B) Overexpression and knockdown efficiency of BMP5 in SW480. Tumor proliferation marker PCNA and migration marker (MMP2 and MMP9) were detected. MMP9 showed no significant difference.

**
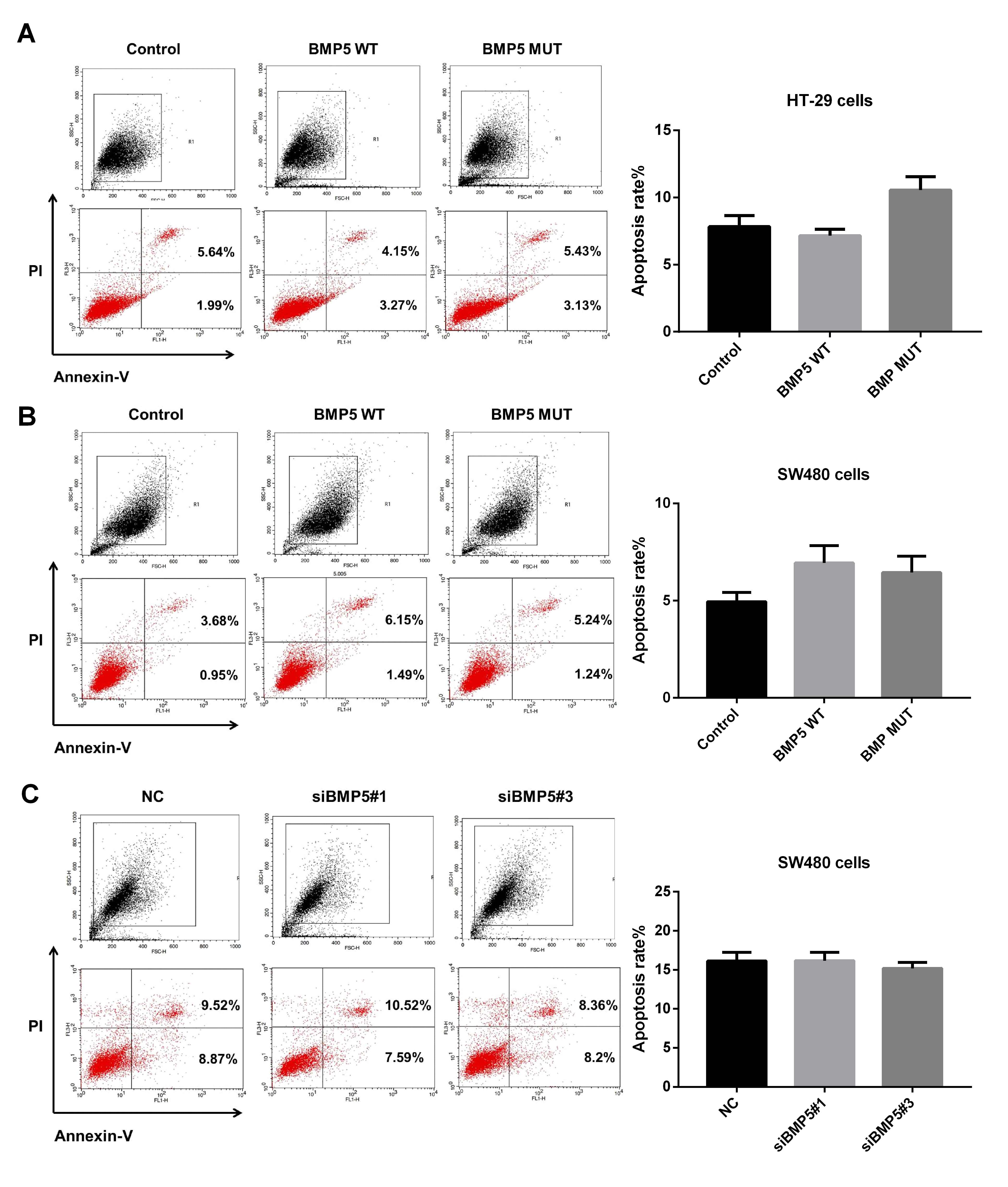
**

**Figure S8. Cell apoptosis of BMP5 in HT-29 and SW480 cells.** The data are showed as mean ± sem.

**
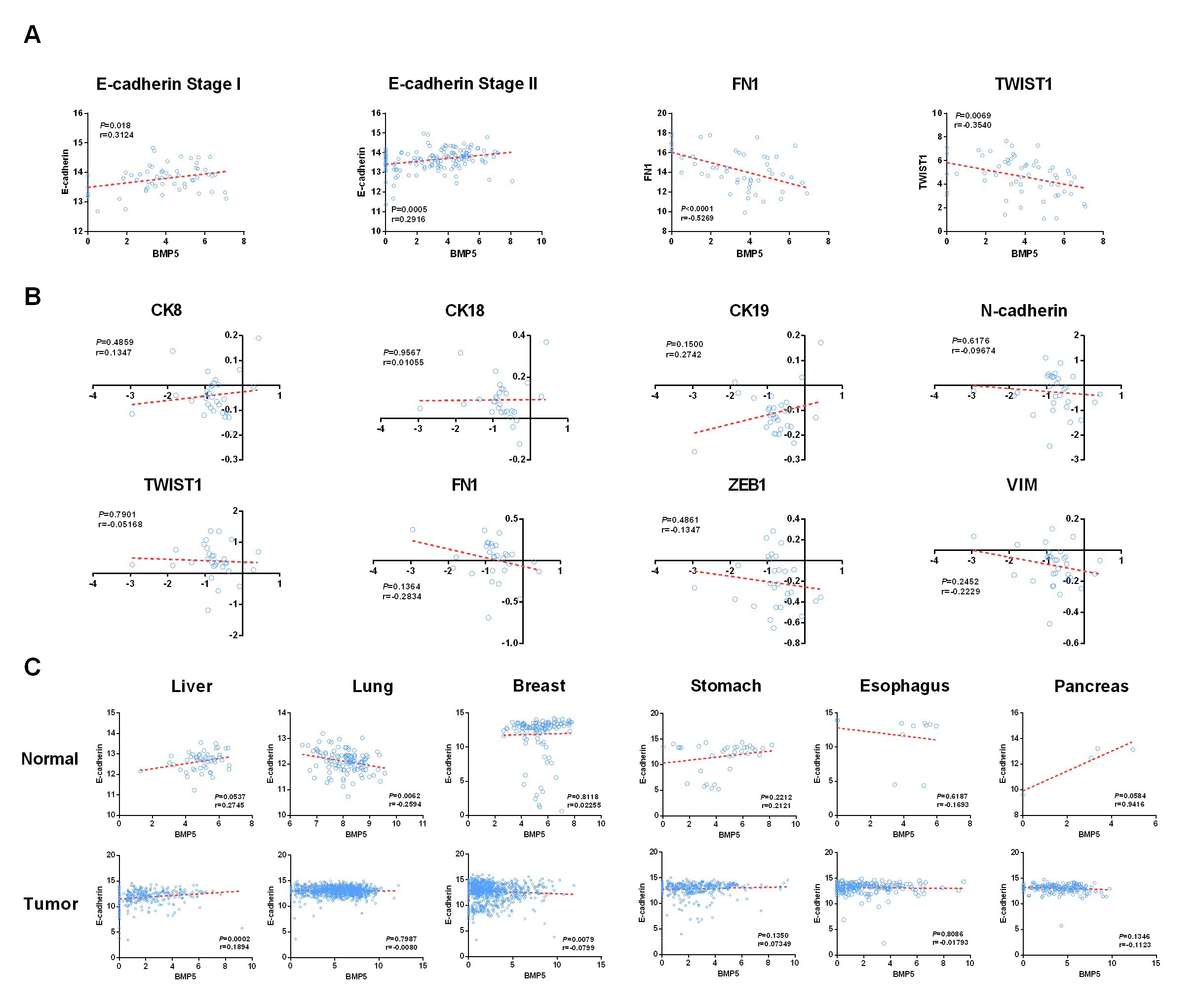
**

**Figure S9. Pearson correlation analysis between BMP5 and EMT markers.** (A) Correlation analysis in tumor samples. (B)Correlation of fold change (Log_2_(Tumor/Normal)) between BMP5 and EMT markers. (C) Correlation between BMP5 and E-cadherin in six tumor types.

**A**


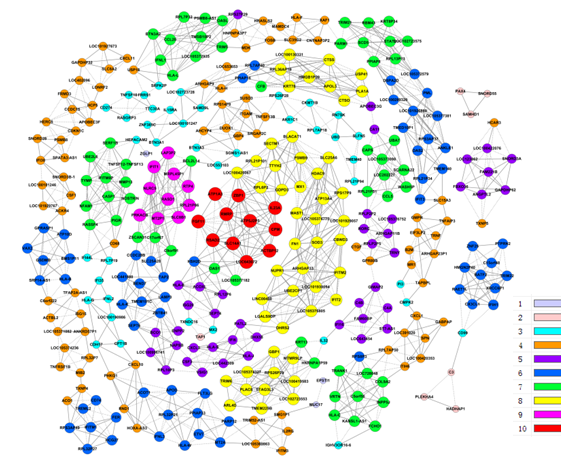


**B**


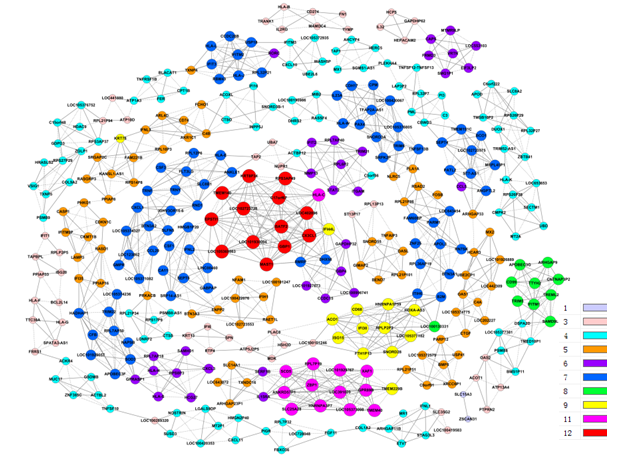


**Figure S10. Coexpression network of control HT-29 (A) and BMP5-expressing HT-29 cells (B).**

**
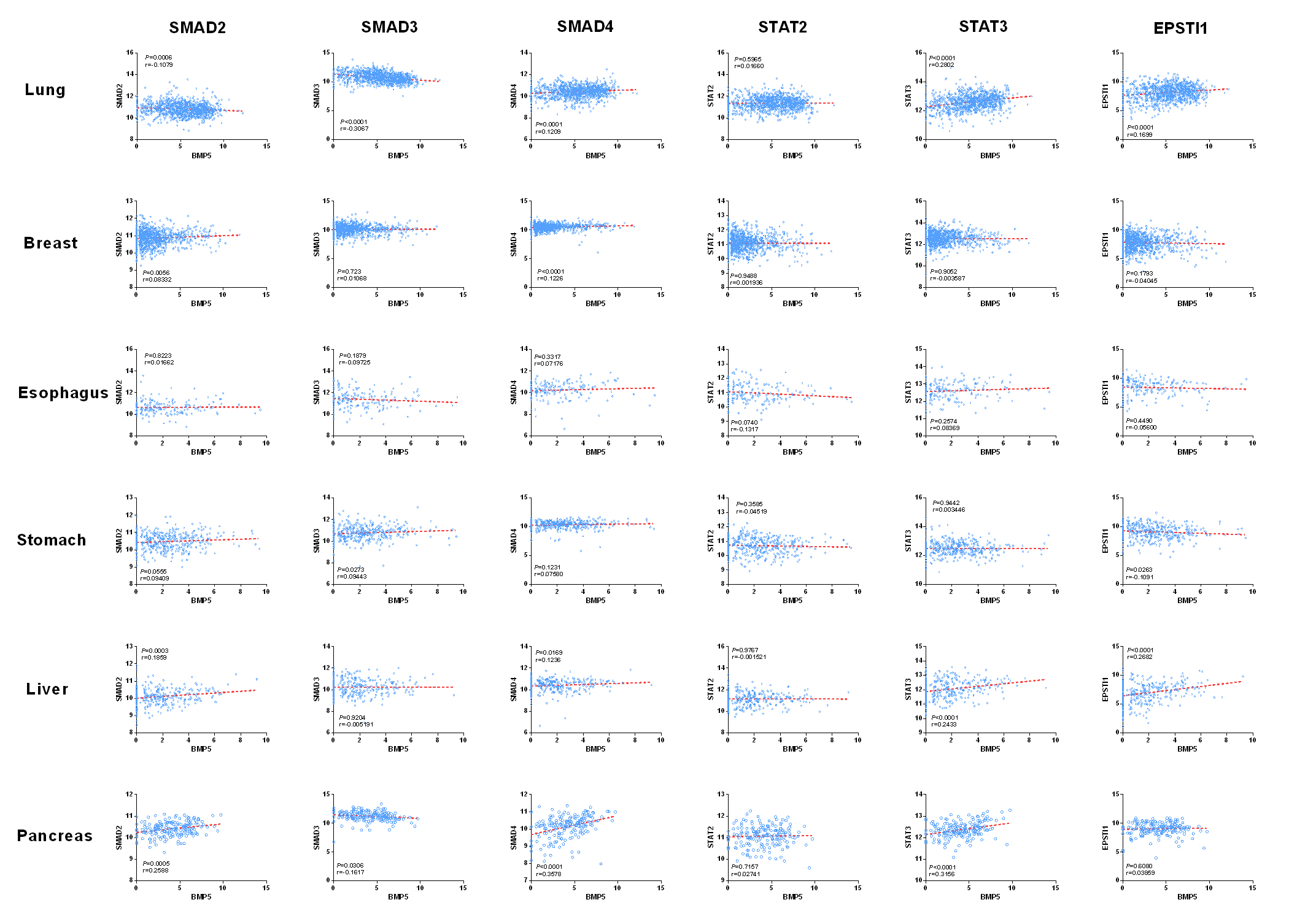
**

**Figure S11.** **Correlation analysis between BMP5 and SMAD or STAT signaling in lung, breast, esophagus, stomach, liver and pancreatic cancer.**
